# Targeting and ultrabroad insight into molecular basis of Resistance-nodulation-cell division efflux pumps

**DOI:** 10.1038/s41598-022-20278-5

**Published:** 2022-09-27

**Authors:** Hooria Seyedhosseini Ghaheh, Mohammad Sadegh Damavandi, Parisa Sadeghi, Ahmad Reza Massah, Taravat Hamidi Asl, Azhar Salari-Jazi, Seyed Hossein Hejazi

**Affiliations:** 1 Department of Pharmaceutical Biotechnology, School of Pharmacy and Pharmaceutical Sciences, Isfahan University of Medical Sciences, Isfahan, Iran; 2 Department of Microbiology, School of Medicine, Isfahan University of Medical Sciences, Isfahan, Iran; 3 Department of Drug Development and Innovation, Behban Pharmed Lotus, Tehran, Iran; 4 Department of Chemistry, Shahreza Branch, Islamic Azad University, P.O. Box 311-86145, Shahreza, Isfahan, Iran; 5 Skin Diseases and Leishmaniasis Research Center, Department of Parasitology and Mycology, School of Medicine, Isfahan University of Medical Sciences, Isfahan, Iran

**Keywords:** Computational biology and bioinformatics, Drug discovery, Microbiology, Structural biology

## Abstract

Resistance-nodulation-cell devision (RND) efflux pump variants have attracted a great deal of attention for efflux of many antibiotic classes, which leads to multidrug-resistant bacteria. The present study aimed to discover the interaction between the RND efflux pumps and antibiotics, find the conserved and hot spot residues, and use this information to target the most frequent RND efflux pumps. Protein sequence and 3D conformational alignments, pharmacophore modeling, molecular docking, and molecular dynamics simulation were used in the first level for discovering the function of the residues in interaction with antibiotics. In the second level, pharmacophore-based screening, structural-based screening, multistep docking, GRID MIF, pharmacokinetic modeling, fragment molecular orbital, and MD simulation were utilized alongside the former level information to find the most proper inhibitors. Five conserved residues, containing Ala209, Tyr404, Leu415, Asp416, and Ala417, as well as their counterparts in other OMPs were evaluated as the crucial conserved residues. MD simulation confirmed that a number of these residues had a key role in the performance of the efflux antibiotics; therefore, some of them were hot spot residues. Fourteen ligands were selected, four of which interacted with all the crucial conserved residues. NPC100251 was the fittest OMP inhibitor after pharmacokinetic computations. The second-level MD simulation and FMO supported the efficacy of the NPC100251. It was exhibited that perhaps OMPs worked as the intelligent and programable protein. NPC100251 was the strongest OMPs inhibitor, and may be a potential therapeutic candidate for MDR infections.

## Introduction

Multidrug-resistant (MDR) bacteria have turned into a significant public health threat. There are major concerns regarding the rise of several gram-negative bacteria, with limited treatment options, for which just a few antibiotics are being developed^1^. One of the most essential resistant components for these bacteria is a set of efflux pumps. Efflux pumps efficiently excrete or diminish the broad range of antibiotics intracellular concentration, generating a considerable resistance. Gram-negative three-layer envelopes, consisting of the inner membrane, periplasmic space, and outer membrane, are the host of tripartite systems of RND multidrug efflux pumps^2,3^. RND efflux pumps generate an extended spectrum of resistance against diverse structural and functional antibiotics, such as β-lactams, fourth-generation cephalosporins, aminoglycosides, trimethoprim-sulfonamides, fluoroquinolones, tetracycline, erythromycin, or other types of antibiotics. They also create MDR bacteria^4^. These systems not only demonstrate a wide range of antibiotic substrates, but also extrude quorum-sensing signal molecules, toxic substances, dyes, biocides, detergents, and antiseptics as their substrates^5,6^.

The development of MDR strains is attributed chiefly to efflux mechanisms. The three parts of these efflux pumps include inner membrane proteins (IMPs) or transporters, outer membrane proteins (OMPs) or channels and outer membrane factor (OMF), and periplasmic membrane fusion proteins (MFPs) or periplasmic adaptor protein (PAP)^7^. The inner part of the efflux pump ensnares antibiotics, which are then transported to the periplasmic section, and eventually, OMP expels antibiotics. These three proteins work together to produce a channel that permits antibiotics to transit from the cytoplasm to the extracellular environment, and their function is crucial to extrude substrate from the cell. Accordingly, every protein of this system can be a target to block efflux pump performance and restore antibiotic power in order to combat MDR bacteria^8^. As a result, possible efflux pump inhibitors should be prioritized to develop a combination of antibiotic therapies and design new antibiotic classes to block efflux pumps. Reverse evolution occurs in the bacterial resistance to the antibiotics rise era, and antibiotics power will be restored via efflux pump potential blockers; therefore, a lower dosage of antibiotics is prescribed so that their adverse effects could be minimized.

There is a large amount of data concerning the X-ray diffraction structures, mutagenesis, normal mode analysis, in silico dynamics, and salt bridge analysis, which have contributed to the discovery of a viable channel gating mechanism^4,9,10^. Research in this field also gave some more data about the performance of residues in TolC^4^.

However, there was a lack of such a comprehensive perspective about the residues engaged in drug efflux, on which we shed light in the present study. Our research concentrated on the most frequent OMPs among RND efflux pumps, including OprM, OprN, OprJ, OprA, and TolC, in order to discover more about the dynamics of the OMFs residues. On the other hand, we believed that some missing points existed concerning the analog residues in the structures of OMFs; thus, this prompted us to employ a huge computational approach for exploring them.

In addition, it was decided to target the most common OMFs in light of these outcomes. To this end, this research has two parts: investigating the OMP of RND efflux pump structures and functions, and targeting the OMP of RND efflux pumps.

In the first part, alignment, 3D conformational alignment, Pharmacophore modeling, and pharmacophore alignment were employed for identifying the conserved residues among five OMFs; consequently, molecular docking and molecular dynamic simulation aimed to display the OMPs dynamic behavior against antibiotics. On the other hand, the molecular basis of the hot spot residues' activity against antibiotics was analyzed via molecular dynamics simulation. The correlation among these outcomes indicated that the conserved residues were hot spots and played a vital role in the efflux performance.

In the second part, pharmacophore-based screening among the conserved hot spot residues was performed in order to screen the ligand that could block all types of OMPs. Multistep virtual screening, GRID MIF, molecular dynamic simulation, and fragment molecular orbital computation were used for discovering the best fit ligand against the conserved residues. Compounds were also screened using pharmacokinetic calculation.

## Methods

### Investigation of OMP of RND efflux pump structures and functions

#### Protein and ligand preparation

The X-ray diffraction of OprM, OprN, OprJ, OprA, and TolC (PDB IDs of 5AZS, 5AZP, 3D5K, and 1EK9, respectively) structures were downloaded from the Protein Data Bank (https://www.rcsb.org/pdb). These proteins were homotrimer, and every monomer had approximately 450 amino acids. In the PDB structures, water molecules were deleted, and hydrogen atoms were added at pH 7 via Discovery Studio software 2.5 (DS, Accerlys Inc, San Diego). The Discovery Studio simulation module was employed to minimize proteins and ligands energy by conjugating the gradient approach and CHARMM force field until the energy gradient fell to below 0.1 cal ${A}^{^\circ -1}$^1,11^.

### Proteins sequences alignment

The sequences of OprM, OprN, OprJ, OprA, and TolC were aligned in the CLC Genomics Workbench version 20.0.4 (Qiagen A/S, Vedbæk, Denmark) program. In this alignment, the conserved residues were discovered. They should include two essential provisions: the first one contained residues conserved among the stated proteins while the second one contained the exposed residues to the inner section of the OMP, which were able to directly interact with the antibiotics^1^.

### Homology modeling

Homology modeling was performed to create the OprA proteins since X-ray crystallography of this protein did not present in the PDB database. OprA protein amino acid sequences were obtained from the NCBI sequence database (http://www.ncbi.nlm.nih.gov) (accession no: ABN92387.1, 516 aa). OprM had more sequence similarity to the OprA proteins; therefore, OprM was applied as a template for homology modeling in the MOE version 2019.0102 software. The modeled structure was validated in the MOE program^12^.

### 3D conformational alignment

This sort of analysis attempts to pinpoint residues' counterparts in the structure of certain proteins that may not be conserved in the protein sequence alignment, but are homolog in 3D structure. 3D conformational alignment was performed to explore the conserved residues' spatial orientation and sequence location via Chimera version 1.16 software among OprM, OprN, OprJ, OprA, and TolC^13^. To advance to the next phase, the conserved residues with relative spatial location in five OPMs were determined and verified.

### Pharmacophore modeling

In order to pinpoint the conserved residues with the two aforementioned criteria (provisions), 3D conformational alignment was used. Moreover, pharmacophore modeling was utilized for verifying the accuracy of the results from earlier stages. Therefore, if the functional groups' pharmacophore of various screened residues overlap, they would be precisely conserved. In this stage, pharmacophore modeling among the conserved residues was applied to discover the functional group pharmacophores in every OMP; subsequently, the modeled pharmacophores were aligned. The alignment would assist us to recognize common pharmacophores and validate the conservatory of screened residues. Pharmacophore modeling and their alignment were done with LigandScout^1,14,15^.

### Molecular docking

An antibiotic library was collected in this step, including tetracycline, meropenem, chloramphenicol, levofloxacin, and azithromycin, which was downloaded from the ChEMBL database. These antibiotics, as the substrates, were perfectly extruded by the chosen RND efflux pumps. The antibiotics were docked in OMPs by the AutoDock Vina 1.1 module of LigandScout 4.3.. The performed grid map, with grid spacing of 1.0 Å, was set at 23 × 23 × 23 points for x, y, and z dimensions for OprM, OprN, OprJ, OprA, and TolC. The center grid box locations were positioned at − 17.821, − 01.471, and − 93.082 for OprM; − 89.543, 15.997, and − 58.088 for OprN; − 35.295, 0.743, and − 21.443 for OprJ; − 19.042, − 01.532, and − 83.108 for OprA; , and 22.653, 76.952, and 59.063 for TolC, at the x, y and z axes, respectively. The Lamarckian genetic algorithm's default settings were utilized, and the number of runs for profile docking was set at 40. The lowest docking affinity complexes for each of the mentioned antibiotic were prepared for MD simulation^14–16^.

### Molecular dynamics simulation

Through the use of MD simulation, we assessed the behavior of OMPs analog residues with substrate antibiotics. The OMP-antibiotic complexes were prepared for 20 ns MD simulation with 2 fs time step using Desmond 2021-2 Linux (Desmond, Schrödinger, LLC, New York, NY, USA). The system builder panel was performed to prepare an orthorhombic simulation box with the TIP3P explicit water model. The system builder panel generated an orthorhombic simulation box with the TIP3P explicit water model and POPC bilayer membrane. The protein surface and boundary of the simulation box's edge were separated with buffer region with a minimum distance of 10 Å. To neutralize the system, with regard to the total charge of the system, Na^+^ and ${\text{CL}}^{-}$ counterions were injected and 150 mM NaCl was applied to maintain the isosmotic salt environment. The system was minimized with 20,000 iterations and a convergence threshold of 1 kcal/mol/Å. Prior to MD simulation, the default relaxation parameters of Martyna–Tobias–Klein barostat and Nose–Hoover Chain thermostat Martyna thermostat were performed to sustain the pressure and temperature, respectively. The minimized system was exposed to a 20 ns MD simulation at 300 K and 1.013 bars utilizing the NPT (Normal Pressure and Temperature) ensemble. MAESTRO (Maestro, Schrödinger, LLC, New York, NY, USA) visually evaluated 3D structures and trajectories. Desmond's Simulation Interaction Diagram module analyzed RMSD, RMSF, and protein–ligand contact (PL-Contact and LP-Contact)^17^.

### Targeting of the OMP of RND efflux pumps

#### Pharmacophore-based screening

The alignment over the modeled pharmacophores was conducive to the determination of the most applicable common pharmacophores. Pharmacophore-based screening was carried out to evaluate the overlapping ligands with those common pharmacophores. Therefore, those ligands could block all types of efflux. The common pharmacophores was applied as a template to discover hit compounds from the NPASS database through LigandScout^18^. The hit compounds were docked in OMPs, using the AutoDock Vina 1.1 module of LigandScout 4.3. The docked hit compounds should gain the proper docking score for all OMPs to pass to the next step^14^.

#### Structural screening

The passed hit compound was applied screen the NPASS database by the infiniSee 2.0.0 program based on the resemblance to NPC226108 with a similarity rate of 75–100, which resulted in a new database^19^.

### Molecular docking

The AutoDock Vina 1.1 module of LigandScout 4.3 was used to dock the newly screened database in the OprM, OprN, OprJ, OprA, and TolC through the aforementioned coordination and algorithm. LigPlot^+^ v.2.2 and Discovery Studio Visualizer version 4.1 software created the interaction profiles between the ligand–protein complexes. The compounds with lower docking affinity that interacted with at least three key residues of OMFs were chosen to be explored; however, they should have interacted with the conserved residues in all OMFs to pass to the next pharmacokinetic calculation step^14–16^.

### GRID MIF

GRID MIF calculation was carried out to track down the extraordinarily low negative docking affinity in ligands. Furthermore, GRID can identify the hydrophobic binding regions essential to design high-affinity ligands and effectively determine polar group sites responsible for ligand selectivity.

GRID is a tried-and-true method for establishing energetically favorable interaction locations on known molecular structures. The intensity and direction of active sites of molecular interactions were determined with molecular interaction fields (MIFs) produced through the GRID force field of FLAP version 2.2.1 software. Molecular interaction fields are 3D energy profiles of the possible interactions between the ligand and protein probes. Protein MIF probes consisted of the N1 probe (amide nitrogen displaying hydrogen bond donor groups with energy values of − 3 kcal/mol), the O probe (carbonyl oxygen exhibiting hydrogen bond acceptor groups with energy values of − 3 kcal/mol), the CRY (It determines mixed hydrophobic/lipophilic with energy values of − 1 kcal/mol), and the DRY probe describing hydrophobic interactions.

The grid spacing, pocket point outside the ligand molecule, and resolution were set to 0.5, 7, and 0.5 Angstrom, respectively. The overlap of ligands of the polar and functional groups with these O or N probes (with the mentioned energy values) would display the interaction associated with the probes energy rate. Additionally, matching with CRY and DRY probes is counted at − 1.0 kcal/mol^1,20,21^.

### In silico pharmacokinetics, pharmacodynamics, and toxicity studies using ADMET predictor

Various physicochemical characteristics were computed for in silico assessment of the research compounds against the standard pharmacokinetic parameters, such as ADME, and their projected toxicities were determined using ADMET Predictor version 9.0 software (Simulations Plus Inc., USA). In a part of this research, the quantitative assessment of drug-like characteristics was computed, such as pKa (negative logarithmic measure of the acid dissociation constant), permeability, lipophilicity, solubility, plasma protein binding, absorption, blood–brain barrier penetration, transporters, dermal and ocular penetration, metabolism, and drug-drug interaction.

Four compounds, namely NPC98538, NPC100251, NPC112380, and NPC4733010, were selected from the docking step since they could interact with the conserved residues in all OMPs. The safety of the compounds is a critical factor in the development of successful medication. The effects of compounds on liver-associated enzymes, such as alkaline phosphatase (ALP), gamma-glutamyltransferase (GGT), aspartate transaminase (AST), alanine transaminase (ALT), and lactate dehydrogenase (LDH) enzymes, were anticipated. In addition, some other properties, such as neurotoxicity, androgen receptor toxicity, allergenicity, mutagenicity, and developmental toxicity, were calculated. The ADMET assessment aimed to determine the dose ranges and define how potential chemicals interact with the human body.

### FMO and PIEDA

Quantum-mechanics calculations of the fragment molecular orbital (FMO) approach were employed to assess paired interaction energy decomposition analyses (PIEDAs) among ligands and proteins.

Kato et al. and Sliwa et al. described the FMO technique^22,23^. In brief, 3D protonation, C- and N-termini capping, and energy minimization with AMBER10: EHT force field were utilized for OprM, OprN, OprJ, OprA, and TolC proteins through MOE program^24^. Facio version 23.1.5 software generated the input file using automated technique fragmentation. In the GAMESS version 2021 R2 Patch 2 software, the FMO was calculated with RHF method and a basis set of 6-31G*^25^. The completed FMO computation file was analyzed in order to assess pair interaction energy decomposition analysis through the use of Facio program. Exchange repulsion (EX), electrostatic (ES), charge transfer with mixed term (CT mix), and total pair interaction (Etot) contributions, as four energy components, were evaluated. The high rate of PIEDA interaction would confirm the efficacy of NPC100251 against RND efflux pump.

In this step, two distinct ligands were chosen to rigorously compare and visualize the interaction between OMPs and their ligands more profoundly. If the critical residues in OMPs produced considerable PIEDA participation with various ligands, these findings confimed the importance of these residues with certainty. On the other hand, the similar quantum energy contribution of crucial conserved residues demonstrated their effect on the efflux functions of all OMPs and proved that these residues were hot spots. Additionally, the PIEDA level could evaluate the ligand's potency against OMPs.

### Molecular dynamics simulation

The MD simulation method was used for NPC100251-OMFs as mentioned previously. RMSD and RMSF were calculated. Molecular Mechanics-Generalized Born Surface Area (MM-GBSA) is an efficient procedure to ascertain binding-free energy and docking precision. The binding-free energy of all the complexes was computated with thermal_mmgbsa.py script in the Schrödinger package the equation below:

$$\Delta {\text{G}}_{{{\text{binding}}}} = {\text{G}}_{{{\text{complex}}}} - {\text{G}}_{{{\text{protein}}}} - {\text{G}}_{{{\text{ligand}}}}$$

The binding-free energy was calculated using the last 10 ns of MD simulation trajectories.

The obtained outcomes were compared with antibiotics-OMPs' MD results to investigate the compound's efficacy in inhibiting the RND efflux pumps.

## Results

### Investigation in OMP of RND efflux pump structures and functions

#### Proteins sequences alignment

Efflux pump OMP sequences alignment revealed some conserved sequences. Based on the CLC Genomics Workbench alignment, a 40.34% identity was discovered among five OMPs types; accordingly, 40% of these five OMF sequences were homolog. However, since the molecular basis of the interaction among antibiotics and OMPs was considered, therefore, among this 40.34% identity, only the residues with the two mentioned provisions were selected. Table 1 and Figure S1 illustrate these sequences and their analogs. Among the four efflux pumps of OprM, OprN, OprJ, and OprA, nine amino acids, namely Ala209, Arg403, Tyr404, Leu412, Leu415, Asp416, Ala417, Arg419, and Ser420, were conserved. Meanwhile, among OMP of OprM, OprN, OprJ, OprA, and TolC, five amino acids, including Ala209, Tyr404, Leu415, Asp416, and Ala417, were conserved. These five residues were equivalent and located on the inner side of all OMPs (red and bold residues in Table 1). Thus, they progressed to the following steps.

**Table 1:** Crucial amino acids and their analogous amino acids in different types of OMPs.

Types of OMPs	Crucial amino acids								
OprM	Ala209	Arg403	Tyr404	Leu412	Leu415	Asp416	Ala417	Arg419	Ser420
OprN	Ala201	Arg396	Tyr397	Leu405	Leu408	Asp409	Ala410	Arg412	
OprJ	Ala204	Arg399	Tyr400	Leu408	Leu411	Asp412	Ala413	Arg415	Ser416
OprA	Ala264	Arg458	Tyr459	Leu467	Leu470	Asp471	Ala472	Arg474	Ser475
TolC	Ala257		Tyr362		Leu373	Asp374	Ala375		

### Homology modeling

To construct the OprA, homology modeling was adopted. The RMSD (Root-mean-square deviation), CA RMSD, atom clashes, and packing score were computed with the optimum values of 0, 0, 0, and 2.19, respectively. The Ramachandran plot was employed to estimate the model adequacy. The MOE program calculated the RMSD, CA RMSD, atom clashes, packing score, and as well as Ramachandran plot. The amino acids proportion in the favored or core regions was 96%; hence, OprA was properly modeled (Figure S2).

### 3D conformational alignment

**Figure 1:**
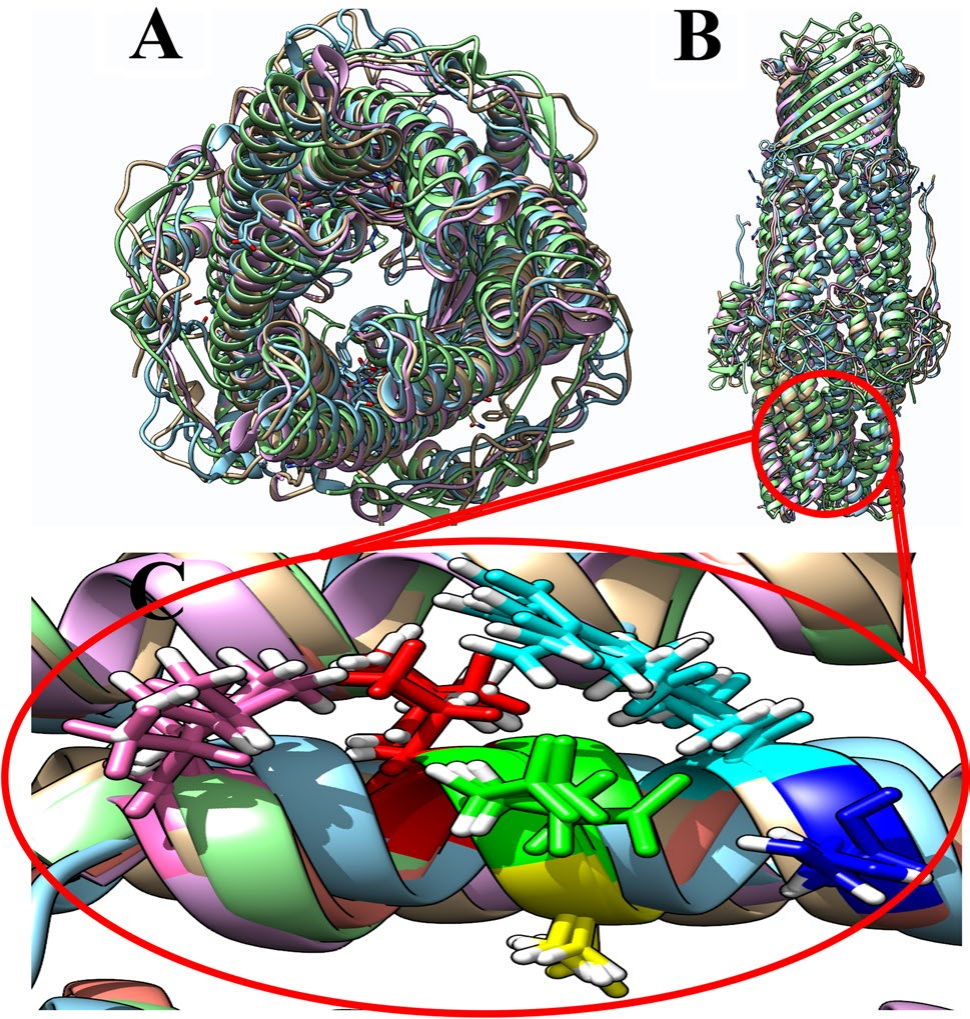
(**A**) and (**B**) 3D conformational alignment of the five class OMP efflux pumps. (**C**) The crucial conserve residue in OPMs. These amino acids pass from the protein sequence alignment level. Leu, Leu, Arg, Asp, Ser, and Ala residues are colored hot pink, red, cyan, green, navy blue, and yellow, respectively.

Amino acids in the five OMP subclasses were analogous in 3D conformational alignment. The conserved amino acids are shown in Fig. 1. The 3D alignment procedure revealed that the equivalent residues in spatial orientation and sequences location were very close. The five conserved residues, including Ala209, Tyr404, Leu415, Asp416, and Ala417 in OprM; Ala201, Tyr397, Leu408, Asp409, and Ala410 in OprN; Ala204, Tyr400, Leu411, Asp412, and Ala413 in OprJ; Ala264, Tyr459, Leu470, Asp471, and Ala472 in OprA; along with Ala257, Tyr362, Leu373, Asp374, and Ala375 in TolC, almost overlapped in the functional groups.

### Pharmacophore modeling

Figure S3 represents pharmacophore modeling for the five conserved residues in each OMP. The potential hydrogen bondings and possible hydrophobic interactions in the five OMFs were modeled to generate the pharmacophore. The alignment of the modeled pharmacophore revealed consistent ratios in angles and distances across the conserved residues' functional groups.

### Antibiotic docking and molecular dynamics simulation

The molecular docking helps us discover the possibilities of ligand conformation and interaction in complex with receptor; however, it is complicated to predict the most likely possibilities. MD simulation is a method for evaluating the stability and dynamics of a ligand-receptor complex under physiological environments. Backbone RMSF and RMSD of MD trajectories, with regard to their initial conformations, showed the proteins' variations and behavior during the MD simulation time^26^. Calculation of protein–ligand contacts indicated the significance of the residues in ligand interactions. In OprA, residues of Glu478, Asp471, Arg474, and Ser475; while for OprJ, residues of Glu214, Asp412, Arg415, Ser416, Asn420 and Glu214; whereas for OprM, amino acids of Gln222, Gln386, Asp416, Arg419, Ser420; whilst for OprN, residues of Arg204, Asp409 and Arg412; and while for TolC, amino acids of Asp374, Thr377 and Thr378 had considerable contribution in different types of interactions than other residues. These interactions consisted of H-bond, hydrophobic, ionic interactions, and water bridges. Furthermore, Lue415 had negligible interaction values with OprM (Figure S4, S5, S6, S7, and S8).

Figure S9 presented the most striking observation to emerge from the RMSD data comparison, illustrating that OMPs-antibiotic complexes had lower fluctuation than OMPs-free ligand as if OMPs were smart proteins. The discussed outcomes were supported by RMSF plots which displayed a wider fluctuation range in some protein loops when there was an antibiotic in the proteins (Figure S10).

The fluctuation and flexibility of the loop-related residues in the antibiotic-OMF complexes mood showed a remarkably higher RMSF for Lue99 in OprM; Ala96 in OprJ; Asp158 in OprA; and Ser262 in TolC in comparison with the ligand-free OMFs. These residues and their loops demonstrated further flexibility in the outer membrane proteins without antibiotics. All these residues were exactly behind the exit gate loop of OMPs, which exhibited that this gate had higher motility. Furthermore, this outcome vouched for swing motion of these gates, which may begin by the mentioned residues. It also confirmed more variation of the OMP-antibiotic complexes in comparison with ligand-free OMPs (Figure S11). More motility and flexibility may effectively aim at antibiotics efflux.

Table S1 demonstrates the average and standard deviation of backbone RMSD for the last 10 ns. The average of the last 10 ns confirmed that the fluctuation of the proteins would increase once antibiotics were within the channel.

### Targeting of the OMP of RND efflux pumps

#### Pharmacophore-based screening

**Figure 2:**
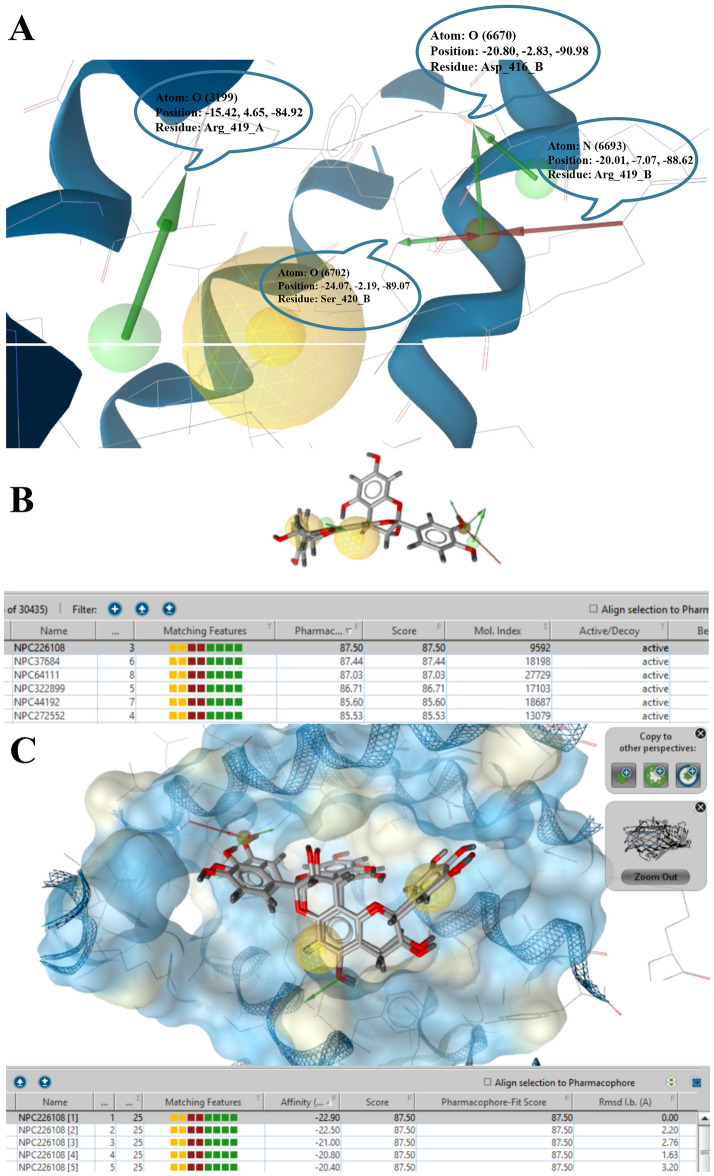
(**A**) Best functional groups of vital conserve residues were selected to make an inclusive pharmacophore. O atom of Arg419, O atom of Asp416, N atom of Arg419 and O atom of Ser420 were chosen to create common pharmacophores. (**B)** Pharmacophore-based screening over the NPASS database was performed in ldb format, and the best-screened molecule was selected based on the pharmacophore fit score. NPC226108 was selected. (**C**) Docking on the best-selected molecule, the docking result demonstrated that the best docking molecule with the lowest energy affinity overlapped with the previously selected pharmacophore.

Figure 2A displays the considered functional groups where common pharmacophores were generated. These pharmacophores were then applied for pharmacophore-based screening. The NPASS converted database was employed for identifying the hit compound, matching the most effective pharmacophores (Fig. 2). NPC226108 was considered as the top hit since it achieved the highest pharmacophore-fit score. This compound was subsequently docked with OMPs to ensure the correctness of the pharmacophore screening. The average docking affinity and Pharmacophore-Fit Score gained were − 22.90 kcal/mol and 87.50, respectively. Therefore, NPC226108 was selected as a pattern for the subsequent stage.

#### Structural screening

The structural screening was utilized for discovering hit compounds with the NPASSv1.0 database (http://bidd2.nus.edu.sg/NPASS/) via infiniSee 2.0.0 software. Based on its similarity to NPC226108, structural screening over the NPASS database resulted in 750 compounds.

### Molecular docking

**Figure 3:**
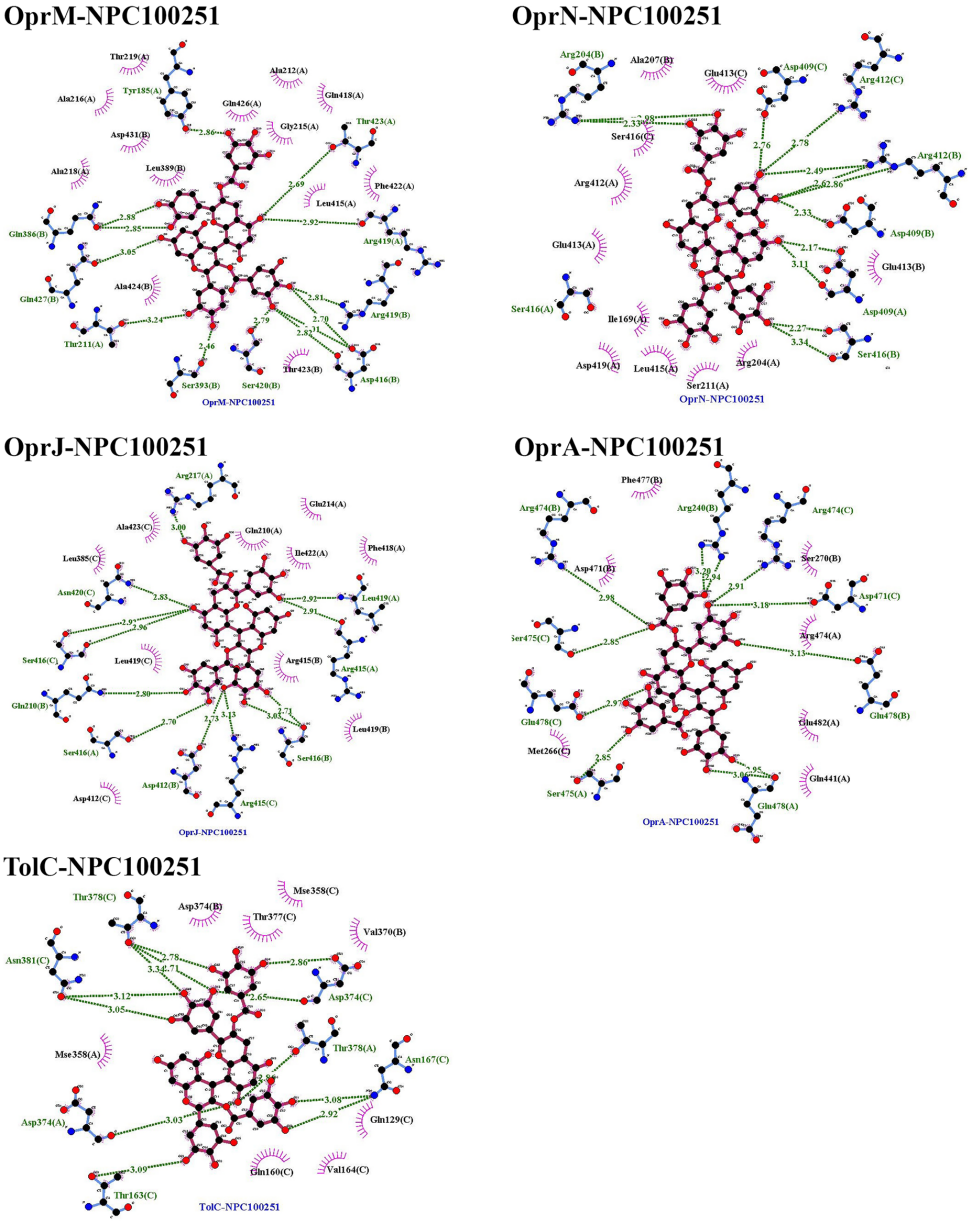
The predicted complex OMPs-NPC100251. The location of the NPs inside the OMPs,hydrogen bonds, and hydrophobic interactions were illustrated. There were 60 complexes that occupied large space in the article so, only NPC100251-complexes with wide interaction were placed in the Figure. Although, all of 60 complexes were assessed for interaction with crucial conserved residues.

Among the 750 collected compounds, 14 achieved a stronger level of affinity binding in the docking process. The gained binding energy of these 14 natural products (NPs) is represented in Table 2. Figure 3 demonstrates hydrogen bonds and hydrophobic interactions for a number of complexes.

**Table 2:** The best binding energy(kcal/mol) obtained from Autodock Vina.

	Binding energy (kcal/mol)				
	OprM	OprN	OprJ	OprA	TolC
NPC300657	− 26	− 25.70	− 23.80	− 24.30	− 22.80
NPC28440	− 22.30	− 22.60	− 21.40	− 22.70	− 20.50
NPC18185	− 21.82	− 21.60	− 21.40	− 22.20	− 22.60
NPC472454	− 22.50	− 23.30	− 20.50	− 22.80	− 21.50
NPC224851	− 21.70	− 19.80	− 20.50	− 21.80	− 19.30
NPC318119	− 24.40	− 23.40	− 20.60	− 21.90	− 25.10
NPC107627	− 22.40	− 20.60	− 21.40	− 21.80	− 20.10
NPC24339	− 21.00	− 20.40	− 21.40	− 20.10	− 18.90
NPC246658	− 20.80	− 21.60	− 18.50	− 18.80	− 19.90
NPC318432	− 21.30	− 19.70	− 19.90	− 20.60	− 20.40
NPC98538	− 27.20	− 24.20	− 22.00	− 22.90	− 25.20
NPC100251	− 27.60	− 24.60	− 24.20	− 23.60	− 25.70
NPC112380	− 25.90	− 24.00	− 26.30	− 24.50	− 24.30
NPC473010	− 21.10	− 21.20	− 21.30	− 20.60	− 20.70

The average of the binding energy of selected compounds was − 21 kcal/mol.

Table 3 depicts the hydrogen bonding, hydrophobic interactions of NPC complexes, and the distance between the ligands and the protein atoms. The interaction of the ligand with the crucial conserved residues is exhibited in Table 4. Among the 14 compounds, only NPC98538, NPC100251, NPC112380, and NPC473010 had wider hydrophobic and hydrogen interactions with conserved residues; thus, they were selected and passed to the next step. NPC100251 had hydrogen and hydrophobic interactions with the most conserved residues in all the OMPs (Fig. 3).

**Table 3:** Hydrogen and hydrophobic interactions of the complexes and distance among ligands and protein.

Complex	Hydrogen bonds		Hydrophobic interaction	
	Residue	Distance (Å)	Amino acids	Distance (Å)
OprM-NPC100251	Thr423(A), Arg419(A), Arg419(B), Asp416(B), Ser420(B), Ser393(B), Thr211(A), Gln427(B), Gln386(B) and Tyr185(A)	2.46–3.24	Ala212(A), Gln426(A), Gly215(A), Gln418(A), Leu415(A), Phe422(A), Thr423(A), Ala424(B), Leu389(B), Ala218(B), Asp431(B), Ala216(B) and Thr219(A)	3.35–4.28
OprN-NPC100251	Asp409(C), Arg412(C), Arg412(B), Asp409(B), Asp409(A), Ser416(B), Ser416(A) and Arg420(B)	2.17–3.34	Glu413(B), Arg204(A), Ser211(A), Leu415(A), Asp419(A), Ile169(A), Glu413(A), Arg412(A), Ser416(C), Ala207(B) and Glu413(C)	3.35–4.02
OprJ-NPC100251	Arg217(A), Leu419(A), Arg415(A), Ser416(B), Arg415(C), Asp412(B), Ser416(A), Gln210(B), Ser416(C) and Asn420(C)	2.70–3.13	Gln210(A), Glu214(A), Ile422(A), Phe418(A), Arg415(A), Leu419(B), Asp412(C), Leu419(C), Leu385(C) and Ala423(C)	3.4–4.54
OprA-NPC100251	Arg420(B), Arg474(C), Asp471(B), Glu478(B), Glu478(A), Ser475(A), Glu478(C) and Arg474(B)	2.85–3.20	Ser270(B), Arg474(A), Glu482(A), Gln441(B), Met266(C), Asp471 and Phe477(B),	3.44–3.86
TolC-NPC100251	Asp374(C), Thr378(A), Asn167(C), Thr163(C), Asp374(A), Asn381(C) and Thr378(C)	2.65–3.34	Val370(B), Gln129(C), Val164(C), Gln160(C), Mse358(A), Asp374(B), Thr377(C) and Mse358(C)	2.88–4.4

Due to the large amounts of data, only some of the better docking complexes were presented in this table.

**Table 4:**
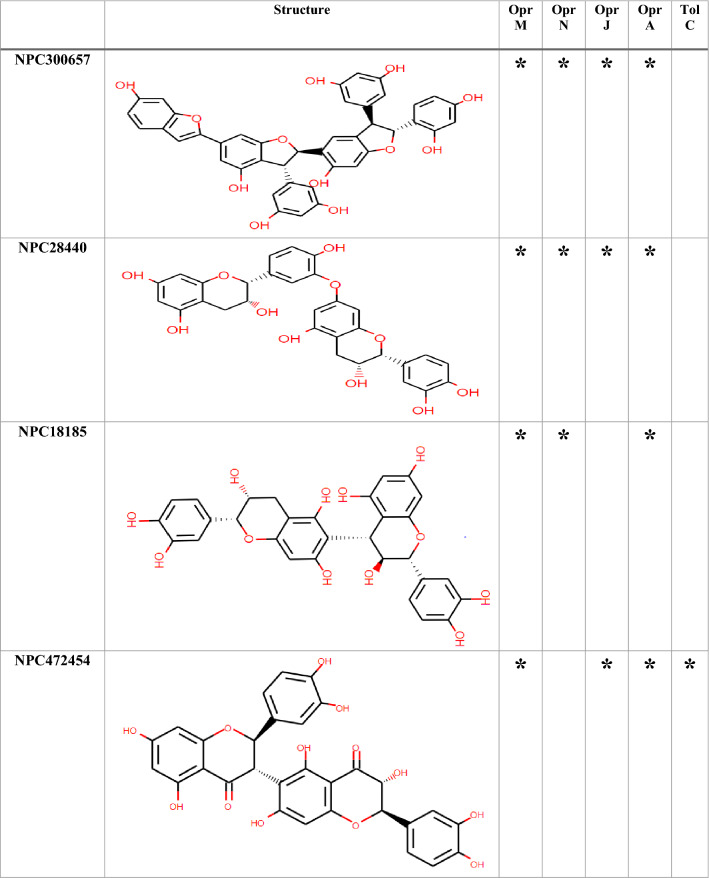

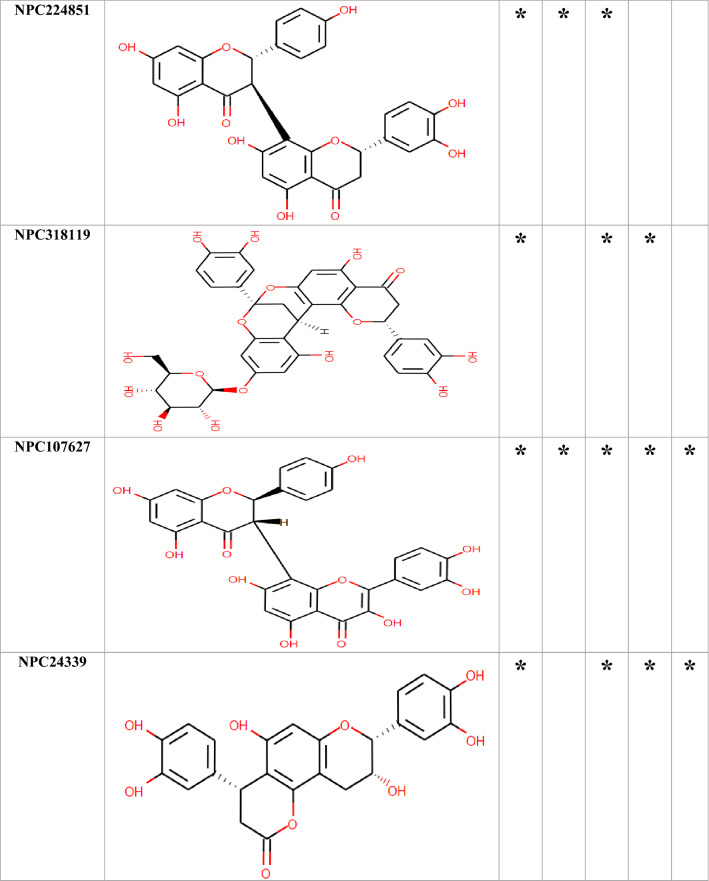

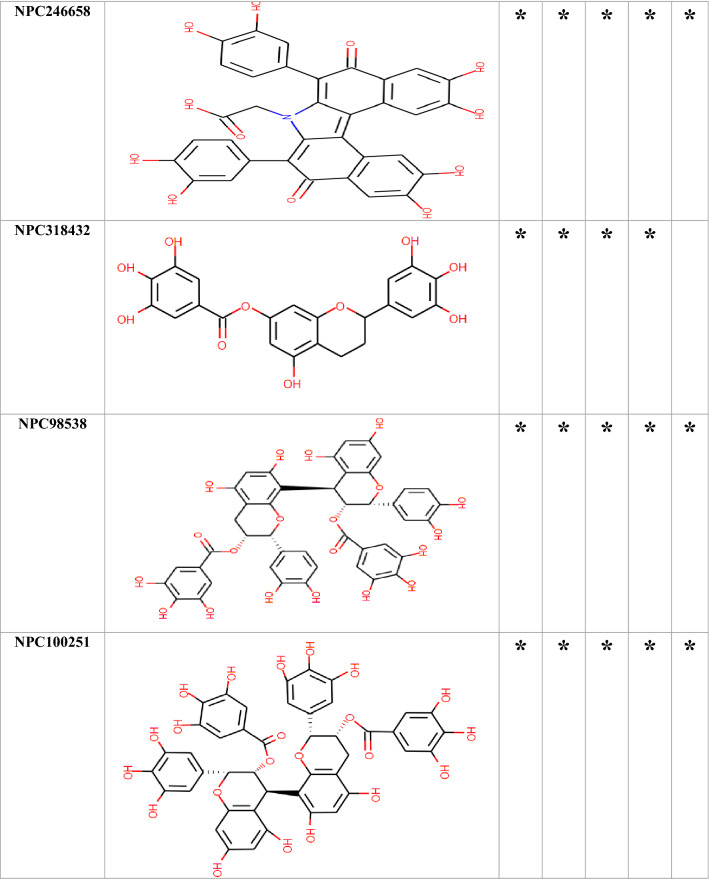

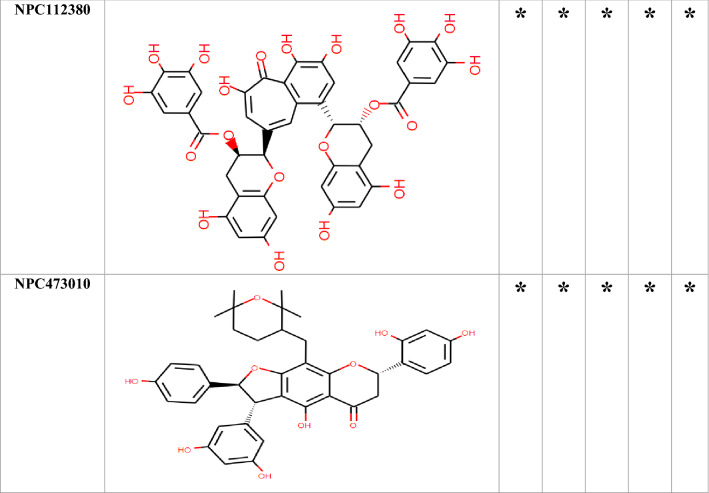
Natural compounds that interact with vital analog residues are highlighted with a star.

According to the interaction with the hot spot conserved residues and low score energy affinity binding, these compounds were selected for the next step. Only four compounds, including NPC98538, NPC100251, NPC112380, and NPC473010, interacted with hot spot conserved residues in OprM, OprN, OprJ, OprA and TolC.

### GRID MIF

The NPC100251 ligand comprised six and 13 O atoms that coincided with the O and N OprM probes in MIF, respectively. Nonetheless, only 13 hydrogen bonds were discovered. Six and eight O atoms of the NPC100251 ligand overlapped with O and N OprN probes in MIF, respectively. However, it formed 13 hydrogen bonds. Note that the five and 12 O atoms of the NPC100251 ligand strongly matched the O and N TolC probes in the MIF, respectively, although it made 12 interactions with the protein in these positions. The DRY probes that characterize the hydrophobic interactions overlapped well with two or three aromatic rings in all the ligands. The CRY probes exhibited very similar interactions to those of DRY. The O, N, and cry probes had energy ranges of 3, 3, and 1 kcal/mol, respectively.

**Figure 4:**
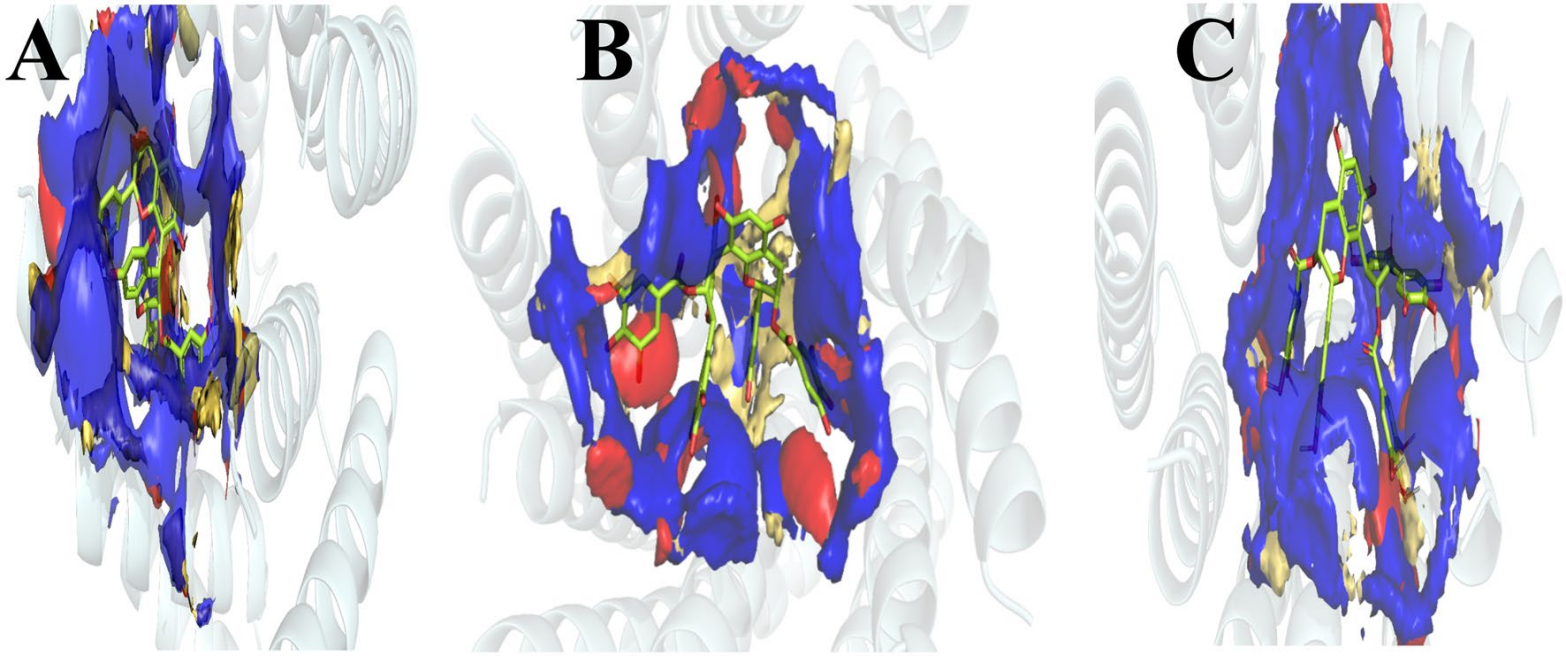
A. NPC10251-OprM. B. NPC100251-OprN C. NPC100251-TolC. GRID MIFs were applied for probes O (red, − 3 kcal/mol), N1 (blue, − 3 kcal/mol), and DRY (brown, − 1 kcal/mol). MIF energy values of 0.0 kcal/mol to − 2.5 kcal/mol are inclined to demonstrate nonpolar interactions, values of < − 2.5 kcal/mol are liable to reveal hydrogen bonding, and larger negative values begin to demonstrate a greater charge interaction. In the figure, most ligand functional groups are correlated with the MIF energy of − 3 kcal/mol in the O or N GRID MIF. Aromatic rings were recruited in DRY and CRY with an MIF energy of − 1 kcal/mol MIF. ΔΔ G binding = ΔΔG hydrogen bond + ΔΔG hydrophobic + ΔΔG vdw + ΔΔG electrostatic It appears as although the hydrogen bond's large number, the hydrophobic bond's very low energy value, and van der Waals (around the hydrogen bond's energy level) generate those docking scores. GRID MIF assists in discovering why the docking scores are very negative. The very low negative energy of the o atoms overlaps with those of the O and N probes, which are not hydrogen bonds; however, they are generated by far more negative values than hydrogen binding.

In all the ligands, O atoms had several contacts with the O and N probes MIF. These contacts were two folds more abundant than hydrogen bonds. Those overlapping O atoms made some hydrophobic interactions with a negative value near the hydrogen bond. The summation of all the hydrogen bonds and hydrophobic and lipophilic interactions produced such a low-affinity range (Fig. 4).

### In silico pharmacokinetics compliance evaluation

The ADMET Predictor version 9.0 software evaluated pharmacokinetic aspects in ADMET terms (absorption, distribution, metabolism, elimination, and toxicity) for four compounds, including NPC98538, NPC100251, NPC112380, and NPC473010, under the following nicknames: NC1, NC2, NC3, and NC4, respectively. Physicochemical and biopharmaceutical properties were used to figure out the ADMET parameters for the studied compounds.

The solubility characteristics of several steps, particularly native water solubility (Table S3), fasting state intestinal fluid, fed state intestinal fluid, and fasted state stomach fluid were determined and expressed quantitatively (Table S4). The MLogP (Moriguchi model of octanol–water partition coefficient) values, octanol–water partition coefficient, octanol–water distribution coefficient, and molecular diffusion coefficient in the water logarithm of the air–water partition coefficient were computed (Table S5). The degree of ionization (pKa), which considerably affects the solubility and permeability, is shown in Table S3. Except for NPC112380 and NPC473010, the two other compounds exhibited solubility and optimum permeability range. The compounds revealed scant BBB‌ penetration.

Lipophilicity refers to a compound's capacity to dissolve in a lipophilic (nonaqueous) environment. Numerous pharmacological drug characteristics influencing ADMET are coordinated to subtend permeability, absorption, solubility, metabolism, distribution, plasma protein binding, elimination, and toxicity. Tables S5 and S6 exhibit various parameters contributing to solubility and permeability, respectively.

Drug compounds confront various membrane boundaries, viz., gastrointestinal epithelial cells, hepatocyte membrane, glomerulus, blood capillary wall, restrictive organ barriers (blood–brain barrier), and target cells. The calculated permeability predictions make it possible to aid the interpretation of the ADMET findings and cell-based bioassays. Among various types of permeability, we could mention permeability over human skin, the MDCK COS permeability, BBB permeability (logBB), jejunal effective permeability, Peff, and rabbit cornea were predicted (Table S5).

Drug bioavailability and drug-drug interactions are both strongly influenced by metabolism.

Metabolism plays a significant role in drug bioavailability and drug-drug interactions. Cytochrome P450 enzymes (CYPs) are undoubtedly the largest prominent family of Phase I metabolizing enzymes responding to the preponderance of drug Phase I metabolic transformations or biotransformation (Table S7).

Moreover, phase II metabolism was investigated on the studied compounds through UGT (uridine 5′-diphosphate-glucuronosyltransferase family) enzyme activity, which attaches glucuronic acid to small molecules and transforms them into a water-soluble shape. This step potentially resulted in the easy elimination of some drugs and their efflux to the outside of body cells.

Table S7 demonstrates that some compounds may be operated as substrates for UGT1A1, 1A3, 1A6, 1A8, 1A9, 1A10, 2B7, and 2B15 whereas UGT1A4 had no substrate and UGT2B7 had only one, being NPC318432. Possible clearance mechanisms are demonstrated in Table S9. None of the compounds had renal clearance, and they were eliminated by hepatic metabolism. Natural compounds' effect on the P-glycoprotein efflux is mentioned in Table S8.

The final four compounds were CYP enzyme inhibitors, which possibly augment some drug plasma concentrations metabolized through CYPs. Accordingly, there might be a potential of drug-drug interaction with these NPs. Moreover, the inhibitory effect of the compounds on the P-gp efflux pumps and organic anion transporting polypeptide 1B1 (OATP1B1) most likely reinforced drug-drug interaction possibilities. Table S9 shows that NC1, NC2, NC3, and NC4 compounds were P-glycoprotein substrates and inhibitors. Acording to the CYP_HLM_Clint, CYP_RLM_Clint, S + CL_Metab, and S + CL_Mech conclusions, represented in Table S10, substance clearance would be most apparently accomplished in the liver.

### Predicted toxicology of the identified lead

NPC98538, NPC100251, NPC112380, and NPC473010 were evaluated to predict the toxicity, allergenicity and mutagenicity in this step. Table 5 lists all the predicted toxicity consequences. There was no evidence on hERG (human ether-a-go-go-related gene) suppression in in silico toxicity; hence, potentially, there would not be an adverse cardiac complication. Additionally, there was no proof of drug-induced phospholipidosis (phospholipid accumulation inside cells), which was associated with deleterious clinical outcomes, such as QT prolongation, myopathy, hepatotoxicity, nephrotoxicity, and pulmonary dysfunction.

**Table 5:** The estimated toxicity risk factors for long-term or high-dose usage of the anticipated active top hit are detailed.

Identifier		NPC98538	NPC100251	NPC112380	NPC473010
**Estrogen receptor (rats)**		Toxic	Toxic	Toxic	Toxic
**Estro_RBA**		2.987	3.004	0.026	0.281
**Androgen receptor toxicity**		Nontoxic	Nontoxic	Nontoxic	Nontoxic
**Andro_RBA**		Nontoxic	Nontoxic	Nontoxic	Nontoxic
**Allergenic skin sensitization (mice)**		Sensitizer	None	None	None
**Allergenic respiratory sensitization in the rat**		Sensitizer	None	None	None
**Fathead minnow lethal toxicity after 96 h of exposure (mg/L)**		0.74	6.824	0.368	1.01
**Tetrahymena pyriformis growth inhibition toxicity (mmol/L)**		2.769	2.816	2.92	2.497
**Biodegradation**		No	No	No	No
**Likelihood of the hERG potassium channel inhibition in human**		No	No	No	No
**Affinity toward hERG K + channel and potential for cardiac toxicity (mol/L)**		5.361	5.553	4.523	5.263
**LD50 for lethal rat acute toxicity (mg/kg)**		427.979	566.325	356.839	57.326
**Tumorigenic dose rat (mg/kg/day)**		2113.603	2542.269	2128.89	1257.639
**Tumorigenic dose mice (mg/kg/day)**		143,596.809	171,930.305	144,474.558	12,973.946
**Triggering the mutagenic chromosomal aberrations**		Toxic	NonToxic	Toxic	Non toxic
**Causing phospholipidosis**		Nontoxic	Nontoxic	Nontoxic	Nontoxic
**Reproductive/developmental toxicity**		Toxic	Nontoxic	Nontoxic	Nontoxic
**Hepatotoxicity**	Levels of ALP enzyme	Elevated	Normal	Normal	Elevated
	Levels of GGT enzyme	Normal	Normal	Normal	Normal
	Levels of LDH enzyme	Normal	Normal	Normal	Normal
	Levels of AST enzyme	Elevated	Normal	Elevated	Elevated
	Levels of ALT enzyme	Normal	Normal	Normal	Normal
**Mutagenicity (pure compound)**	TA97 and/or TA1537 strains of *S. typhimurium*	Negative	Negative	Negative	Negative
	TA98 strain of *S. typhimurium*	Negative	Negative	Negative	Negative
	TA100 strain of *S. typhimurium*	Negative	Negative	Negative	Negative
	*S. typhimurium* and/or WP2 uvrA strain of *E. coli*	Negative	Negative	Negative	Negative
	TA1535 strain of *S. typhimurium*	Negative	Negative	Negative	Negative
**Mutagenicity (microsomal rat liver metabolites)**	TA97 and/or TA1537 strains of *S. typhimurium*	Negative	Negative	Positive	Negative
	TA98 strain of *S. typhimurium*	Negative	Negative	Negative	Negative
	TA100 strain of *S. typhimurium*	Negative	Negative	Negative	Negative
	TA102 strain of *S. typhimurium*	Positive	Negative	Negative	Negative
	TA1535 strain of *S. typhimurium*	Negative	Negative	Negative	Negative

NPC100251 and NPC112380 were found to be nontoxic in the potential reproductive toxicity. Drug-induced hepatotoxicity originates from elevated levels of AST, ALT, ALP, and LDH enzymes, as well as Ser_AlkPhos or Ser_GGT, leading to acute and chronic liver diseases. Only NPC100251 had a standard value of these enzymes, and the rest had an elevated level at least for one enzyme. The compounds were not toxic to androgen and estrogen receptors; therefore, perhaps they did not diminish sperm concentration. Except for NPC24339, the other three compounds were not skin- or respiratory-sensitizing agents.

The mutagenicity via the Ames test on various *Salmonella typhimurium* strains indicated that the three compounds (except NPC100251) were mutagenic for strain; however, NPC100251 had no mutagenic effect in all the assays in this field.

NPC100251, as the best compound (among NPC98538, NPC100251, NPC112380, and NPC473010), was screened using ADMET Predictor version 9.0 software. The other compounds were omitted after determining undesirable pharmacokinetic features. Therefore, NPC100251 was prepared for molecular dynamics simulation.

### FMO and PIEDA

**Figure 5:**
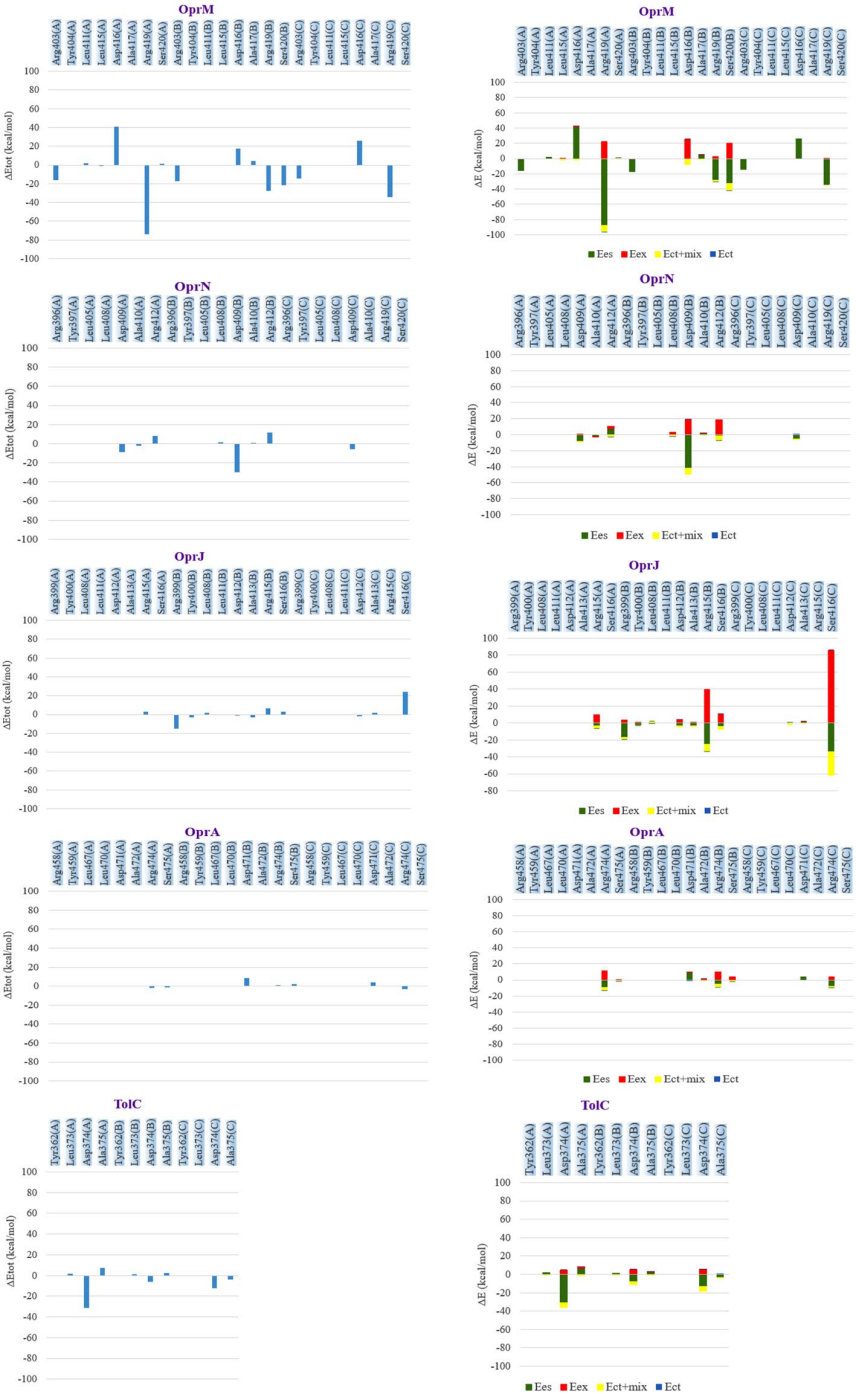
FMO PIEDAs calculation after docking with NPC 226,108. Left hand; profile interaction in PyMOL. Middle bar; ΔEtot (kcal/mol) for every residue. The right-hand bar graph depicts the PIEs and PIEDAs of the crucial OMP residues with NPC226108. Green, red, dark blue, yellow, and light blue colors are reflected in the electrostatic, exchange repulsion, charge transfer, Ect + mix, and E total terms, respectively.

**Figure 6:**
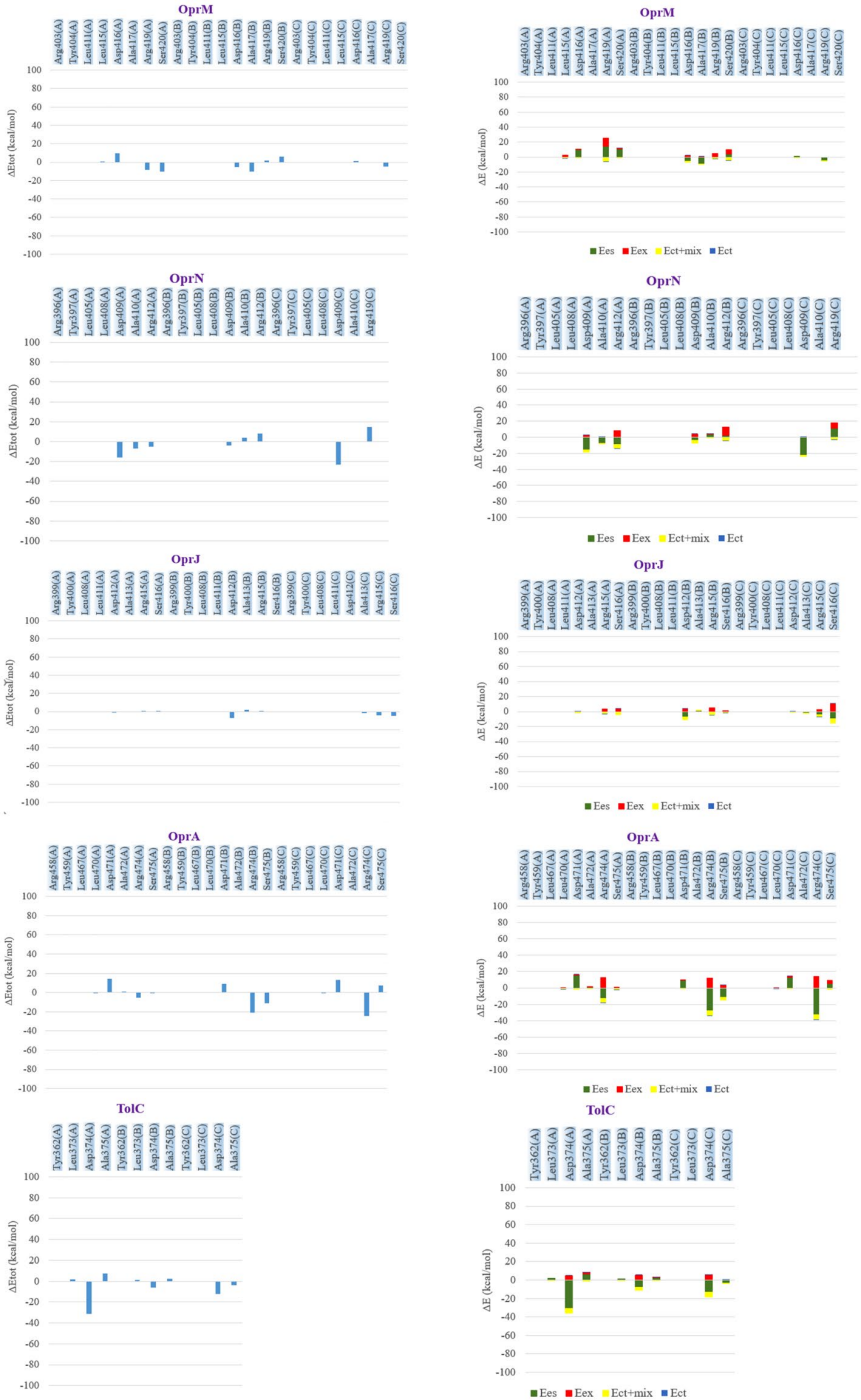
FMO PIEDA computation in complex of NPC100251-OMPs. The right-hand bar concept reveals the PIEs and PIEDAs of the OMP vital residues with NPC100251 (second set). The electrostatic, exchange repulsion, charge transfer, Ect + mix, and E total terms are green, red, dark blue, yellow, and light blue, respectively. Similar residues were involved and resembled results with the first set gained.

NPC100251, as a final compound, was considered for investigation of inhibitory effects on OMFs. The PIEDA-related findings are depicted in Figs. 5 and 6. The residues participating in the interaction with ligands supported the results of the preceding stages. For OprM (complexed with NPC226108), Arg403 (A), Lue411 (A), Lue415 (A), Asp416 (A), Arg419 (A), Ser420 (A), Asp416 (B), Ala417 (B), Arg419 (B), Ser420 (B), Arg403 (C), Asp416 (C), and Arg419 (C) had a more significant PIEDA contribution to ligand interaction than the other residues. The OprN amino acids, Asp409 (A), Ala410 (A), Arg412 (A), Lue408 (B), Asp409 (B), Ala410 (B), Arg412 (A), and Asp409 (C) exhibited a robust affinity with NPC226108. In OprJ, NPC226108 was associated with Arg415 (A), Arg399 (B), Tyr400 (B), Lue408 (B), Asp412 (B), Ala413 (B), Arg415 (B), Ser416 (B), Asp412 (C), Ala413 (C), and Ser416 (C). NPC226108 had a remarkable correlation with Arg474 (A), Ser475 (A), Asp471 (B), Ala472 (B), Arg474 (B), Ser475 (B), Asp471 (C), and Arg474 (C) in OprA. TolC amino acids, namely Lue373 (A), Asp374 (A), Ala375 (A), Lue373 (B), Asp374 (C), and Ala375 (C), demonstrated a substantial interaction with NPC226108. The second set of FMO PIEDA quantum calculations was performed on the NPC100251-OMP complexes in order to determine the docking precision and validate the vital conserved residue functionality. FMO computations perfectly illustrated that NPC100251 had a significant ES amount and a perceptible CT amount, suggesting strong attractive hydrogen bonds between the residues and inhibitors; hence, NPC100251 was a strong inhibitor for OMPs. Both FMO sets displayed the performance of the crucial conserved residues.

### MD simulation for NPC100251

Figure S9 exhibits backbone RMSD plot in the presence of antibiotics and NPC100251. These graphs confirm that NPC100251 can considerably reduce the motility of the protein and make a strong complex.

RMSF in Figure S10 displays that the OMPs residues have less fluctuation in OMPs-NPC100251 than OMPs-antibiotics and ligand-free OMPs formed protein conformational stability. The small standard deviations and average of RMSD confirmed that OMFs structure was stabilized in a complex with NPC100251; as a result, this compound could block OMPs of RND efflux pumps.

Protein–ligand interaction graphs in Figures S4, S5, S6, S7, and S8 demonstrate the high-level interaction (especial hydrogen bond) of the NPC100251 with hotspot residues in all the three monomers of the five OMPs.

MM-GBSA binding free energy of all the complexes is shown in Table S2. The lowest binding energy belonged to NPC100251 in comparison to the antibiotics-OMFs complexes. The negative values of MM-GBSA binding free energy implied that the production of the stable complexes was spontaneous. These results also confirmed that NPC100251 could perfectly bind to OMPs, thereby being able to efficiently block them.

## Discussion

The invention and commercialization of an efflux pump inhibitor, which has resulted in the rehabilitation and reinforcement of antibiotic effectiveness and potency against this resistant type, would profoundly impact human health and generate substantial socioeconomic advantages^8^. These constraints and fundamental necessities were the motivation to mine natural resources as a novel lead efflux pump inhibitor. Additionally, effective blocking of the RND efflux pumps necessitated finding more information about their residues' structure and functional performance. Recent years have seen an enormous growth in the interest in study about different types of RND efflux pumps. One of the most enduring questions for which research has had to seek answers is how residues of the outer membrane protein in an efflux pump contributed their role in the performance of these proteins. Furthermore, another question that needs to be answered is whether OMPs have equivalent conserved residues with similar performances. However, a few researchers have addressed these questions and there have been no controlled computational studies that have simultaneously investigated the interaction of five types of OMP with their substrate antibiotics. The first level of this study, investigation of OMP of RND efflux pump structures and functions, was performed to identify residues significance in the interaction with antibiotics. Proteins sequence multiple alignments over OMP of RND efflux pumps were conducive to locating some residues (Table 1) that were conserved among different species of OMPs. The stringent 3D conformational alignment verified that the following five screened residues (with the two aforementioned provisions) were equivalent in spatial orientation and location, including Ala209, Tyr404, Leu415, Asp416, and Ala417 in OprM; Ala201, Tyr397, Leu408, Asp409, and Ala410 in OprN; Ala204, Tyr400, Leu411, Asp412, and Ala413 in OprJ; Ala264, Tyr459, Leu470, Asp471, and Ala472 in OprA; as well as Ala257, Tyr362, Leu373, Asp374, and Ala375 in TolC. Afterwards, common pharmacophores among OMPs were modeled over the functional groups of these amino acids. The modeled pharmacophores overlapped with all OMPs, which verified the prior alignment steps results. The first-level docking and MD simulation were carried out to investigate the interaction between the conserved residues and antibiotics. Among the crucial conserved residues in MD simulation, the followings had a key role in interaction with azithromycin, levofloxacin, meropenem, chloramphenicol, and tetracycline: Asp471 and Arg474 in OprA; Glu214, Asp412, Arg415, Ser416 and Asn420 in OprJ; Gln222, Gln386, Asp416, Arg419, and Ser420 in OprM; Arg204, Asp409, and Arg412 in OprN; Asp374, Thr377, and Thr378 in TolC. Hence, based on the molecular dynamics simulation, only some conserved residues had kay performances interacting with antibiotics; thus, these residues were both hot spots and conserved residues. The hot spot conserved residues included Asp471 and Arg474 in OprA; Asp412, Arg415, and Ser416 in OprJ; Asp416, Arg419, and Ser420 in OprM; Arg204, Asp409, and Arg412 in OprN; as well as Asp374 in TolC. The above-mentioned hot spot residues were situated in the C-terminal domain, suggesting that this is a probably evolutionary ancestral sequence. These outcomes seem to be consistent with those of other papers^4^ which found Asp374 in TolC and Asp416 in OprM. Discused papers also verified that these residues were hot spots and that their deletion via mutation could decrease the efflux performance. The key role of the Asp409 and Arg412 as the hot spot residues at the periplasmic gate of OprN were also shown^9^.

Another unanticipated result was the higher range of fluctuation in backbone protein RMSD in the OPMs-antibiotics complex compared to that of the ligand-free protein. This outcome was contrary to our expectations since, in the usual mood, the presence of ligands in protein would decrease the motility and fluctuation of protein^26^. On the other hand, it semmed as if OMPs were aware of antibiotics' presence, thereby fluctuating strongly to throw out the ligands. The RMSF-associated findings verified more flexibility in some of the OMPs loops in the OMPs-antibiotic complex moods. The residues of Lue99 in OprM, Ala96 in OprJ, Asp158 in OprA, and Ser262 in TolC behind the exit gate demonstrated more RMSF, which resulted in higher swing motility of the exit gate in OMPs-antibiotic complex moods in comparison with that of the counterpart residues in other ligands-free OMPs. More motility and flexibility of OMPs’ loop and sequences may effectively aim to extrude antibiotics.

The second level of the present study, targeting the OMP of RND efflux pumps, concentrated on targeting the most common RND efflux pumps with the aim of gaining data.

In contrast to this work, a few number of studies have investigated inhibitors of the inner membrane proteins of efflux pumps. However, they did not engage quantum mechanics calculations alongside MD simulation^27–30^. These papers have focused on compounds efficacy on a single type of efflux pump and they did not perform comprehensive targeting for the RND efflux pump family^28,30–35^. In the first level, hotspot residues, critical conserved residues, and their functions that served a prominent part in OMPs' performance were discovered. NPC226108 was screened in the second level through pharmacophore-based screening. The multistep docking process aimed to screen the compounds with very low negative affinity at the second level. According to the GRID MIF evaluation of the ligands, several interactions with a remarkably low negative value occurred that overlapped with the OMPs’ MIF probes. The MIF analysis revealed the majority of the ligand polar groups were engaged in the interactions, ligand lipophilic groups were positioned in the proteins lipophilic space, there was optimal steric matching between the ligand and the receptor, and clashing interactions were not produced. All of the above-mentioned matters explain why natural compounds had very low negative values in docking. Pharmacokinetic properties facilitated the screening process. Among the four compounds passing in the pharmacokinetic level prediction, NPC98538 and NPC100251 demonstrated the optimized range of solubility and permeability. Since BBB penetration was low, none of the selected compounds were proper for brain infection. According to toxicology predictions, phospholipidosis and hERG toxicity was not observed. NPC100251 and NPC112380 did not have reproductive toxicity and only NPC100251 displayed standard levels for hepatic enzymes, including levels of AST, ALT, ALP, and LDH enzymes, along with Ser_AlkPhos or Ser_GGT. Moreover, in the Ames mutagenicity test, NPC100251 did not have a mutagenic effect on different types of *Salmonella typhimurium* strains. Altogether, NPC100251 was selected for MD simulation owing to its desirable pharmacokinetic properties.

FMO outcomes displayed that NPC100251 could perfectly bind to the hot spot conserved residues. These results were also consistent with those of previous steps and verified the mentioned residues impact. Additionally, the first set of involved residues implicated similar energy affinity with the second set FMO PIEDA. The crucial conserved residues in PIEDA significantly contributed higher level of energies than the other residues in outer membrane proteins.

The FMO observations completely validated the same template in the efflux pumps for PIEDA interaction with ligand. This technique demonstrated that several Arg, Leu, and Asp amino acids contributed vital role to ligand interactions. Two sets of FMO calculations with two different ligands, being NPC226108 and NPC100251, were employed to determine the accuracy of docking and critical residues selection. This option illustrated that the hot spots of several OMPs had the same quantum behavior when exposed to various antibiotics and ligand types. The same correlation was observed by comparing the equal conserved residues in PIEDA calculations of other OMPs. These conclusions supported the initial alignment, 3D conformational alignment, and pharmacophore modeling findings. The MD simulation of OMPs-NPC100251 (OprM, OprN, OprJ, OprA, and TolC) indicated that NPC100251 was a proper ligand for blocking the OMPs. NPC100251 compound formed strong and stable complexes with OMPs according to RMSD and RMSF graphs. MM-GBSA binding free energy was remarkable in NPC100251 and was lower than the other OMPs-antibiotic complexes. P-L contact graph during the MD simulation time scale validated that NPC100251 simultaneously formed strong hydrogen binds with hot spot residues (especially hot spot of Asp and Arg in A, B, and C chains) of all three monomers of OPMs. Thus, NPC100251 potentially competed with substrate antibiotics to bind with OMPs and block the efflux of antibiotics. This compound could be therefore proposed as a possible lead for developing future therapeutic candidates.

## Conclusion

The crucial conserved residues as well as hot spot residues were investigated to mine more information about their function in the outer membrane proteins of RND efflux pumps. Highly conserved residues in five subclasses of outer membrane proteins were discovered, whose roles in efflux performance were investigated. Furthermore, the residues responsible for swing motility of the exit gate were discovered. Interestingly and beyond the expectation, it was exhibited that perhaps OMPs of RND efflux pumps worked as the intelligent and programable protein. These outcomes were performed to identify a compound that could inhibit the majority of RND efflux pumps, restore antibiotic strength, and recursively evolve this type of resistance in gram-negative bacteria. FMO PIEDA quantum calculation was conducted on such a large system as the most intricate computation. To the best of our knowledge, this is the first study using FMO for scrutinizing the OMPs of RND efflux pumps. NPC100251 was found to be the strongest OMPs inhibitor among the 14 NPs, and may be a potential therapeutic candidate for MDR infections. The highly conserved residues and their pharmacophore can be subject to further study as a potentially effective target.

According to the obtained findings herein, there is hope to raise awareness about the full scope of virtual screening for the acquisition of hit compounds with highly efficient biological activity. Natural products were chosen owing to their accessibility, whose extraction from their sources was commonly more cost–effective than synthesis of small molecules.

## Supplementary Information

Supplementary Information.

## Data Availability

The authors confirm that the data supporting the findings of this study are available within the manuscript [and/or] its supplementary materials.
