# Supplementary material for: Targeting and ultrabroad insight into molecular basis of Resistance-nodulation-cell division efflux pumps
Source: Sci Rep. 2022 Sep 27;12:16130. doi: 10.1038/s41598-022-20278-5 (PMC9515154; doi:10.1038/s41598-022-20278-5)
Supplement: Supplementary file 1 — Supplementary Information. [file 41598_2022_20278_MOESM1_ESM.docx]

**Supplementary**

Targeting and ultrabroad insight into molecular basis of Resistance-nodulation-cell division efflux pumps

Hooria Seyedhosseini Ghaheh^1†^, Mohammad Sadegh Damavandi^2†^, Parisa Sadeghi^2, 3^, Ahmad Reza Massah ^4^, Taravat Hamidi Asl ^5^, Azhar Salari-Jazi* ^3^, Sayed Hossein Hejazi*^5^

^1^ Department of Pharmaceutical Biotechnology, School of Pharmacy and Pharmaceutical Sciences, Isfahan University of Medical Sciences, Isfahan, Iran.

^2^ Department of Microbiology, School of Medicine, Isfahan University of Medical Sciences, Isfahan, Iran.

^3^ Department of Drug Development and Innovation, Behban Pharmed Lotus, Tehran, Iran.

^4^ Department of Chemistry, Shahreza Branch, Islamic Azad University, Isfahan, 86145-311, Iran.

^5^ Skin Diseases and Leishmaniosis Research Center, Department of Parasitology and Mycology, School of Medicine, Isfahan University of Medical Sciences, Isfahan, Iran.

^†^ These authors have contributed equally to this work and share first authorship.

*** Corresponding authors**;

Prof., Sayed Hossein Hejazi

Skin Diseases and Leishmaniosis Research Center, Department of Parasitology and Mycology, School of Medicine, Isfahan University of Medical Sciences, Isfahan, Iran

Tel; 00983213292522

E-mail: [hejazi@med.mui.ac.ir](mailto:hejazi@med.mui.ac.ir)

Dr. Azhar Salari-Jazi

Department of Drug Development and Innovation, Behban Pharmed Lotus, Tehran, Iran.

[Azhar_sallari@yahoo.com](mailto:Azhar_sallari@yahoo.com)


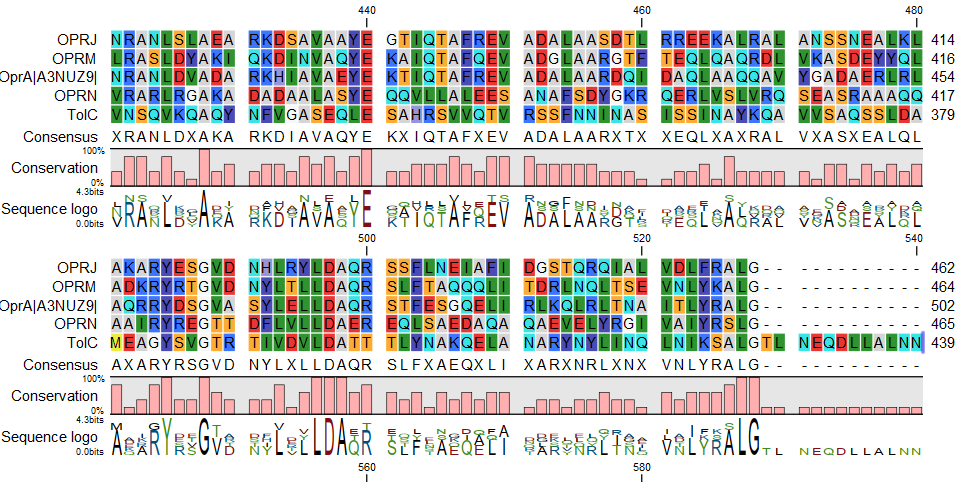


Figure S1-Alignment across all types of the OPMs of efflux pumps.


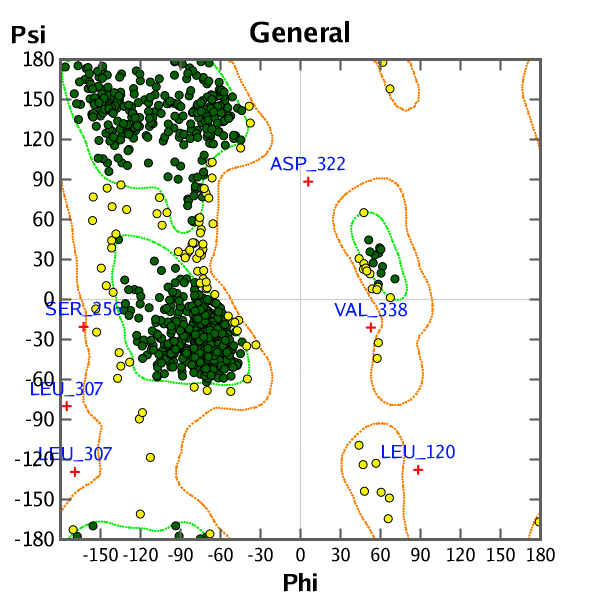


Figure S2-Modeled structures’ quality Evaluating revealed that frequencies of 96, 3.67 and 0.33 percent were attained for favored, allowed and outlier zones, respectively.

| 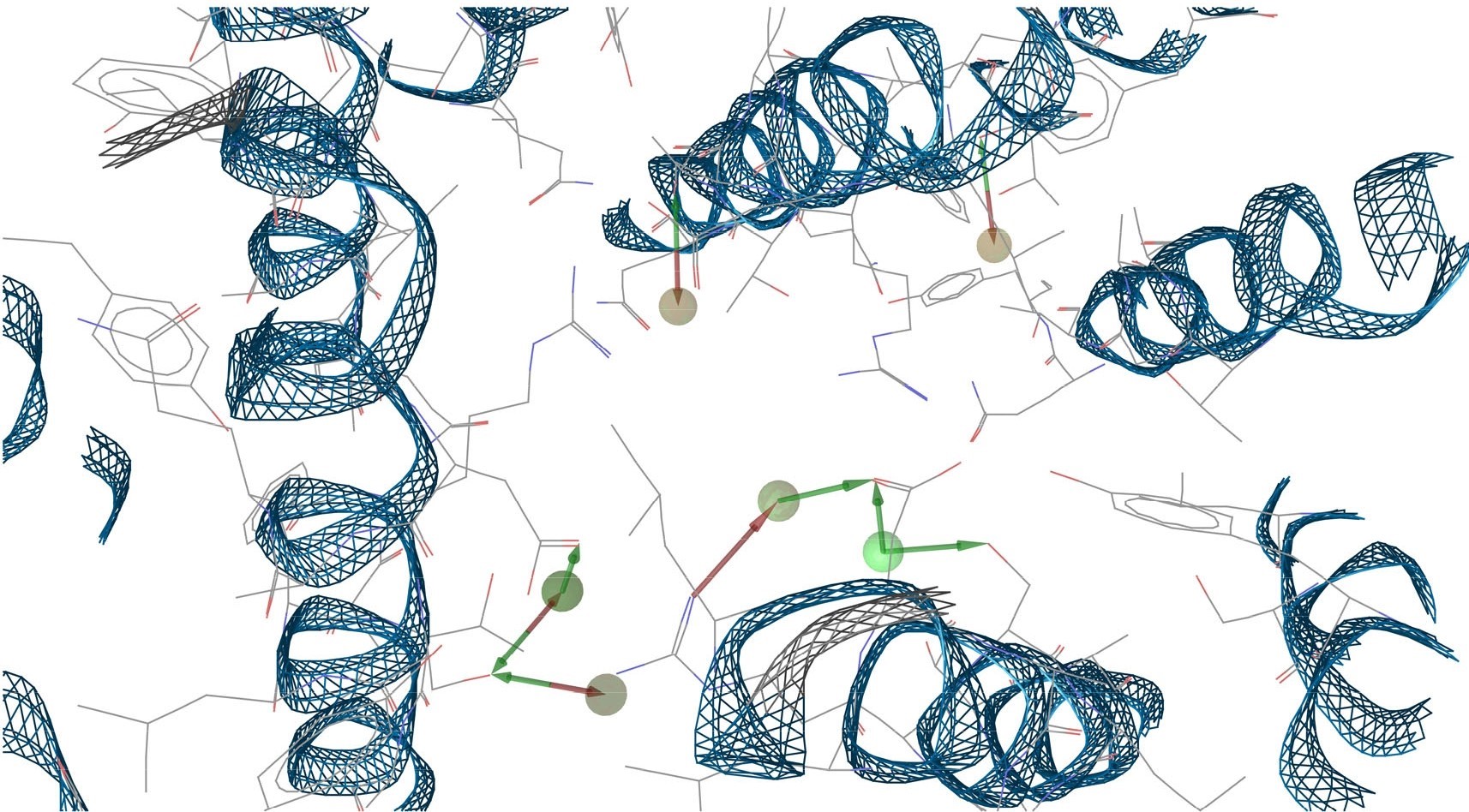 |
| --- |
| 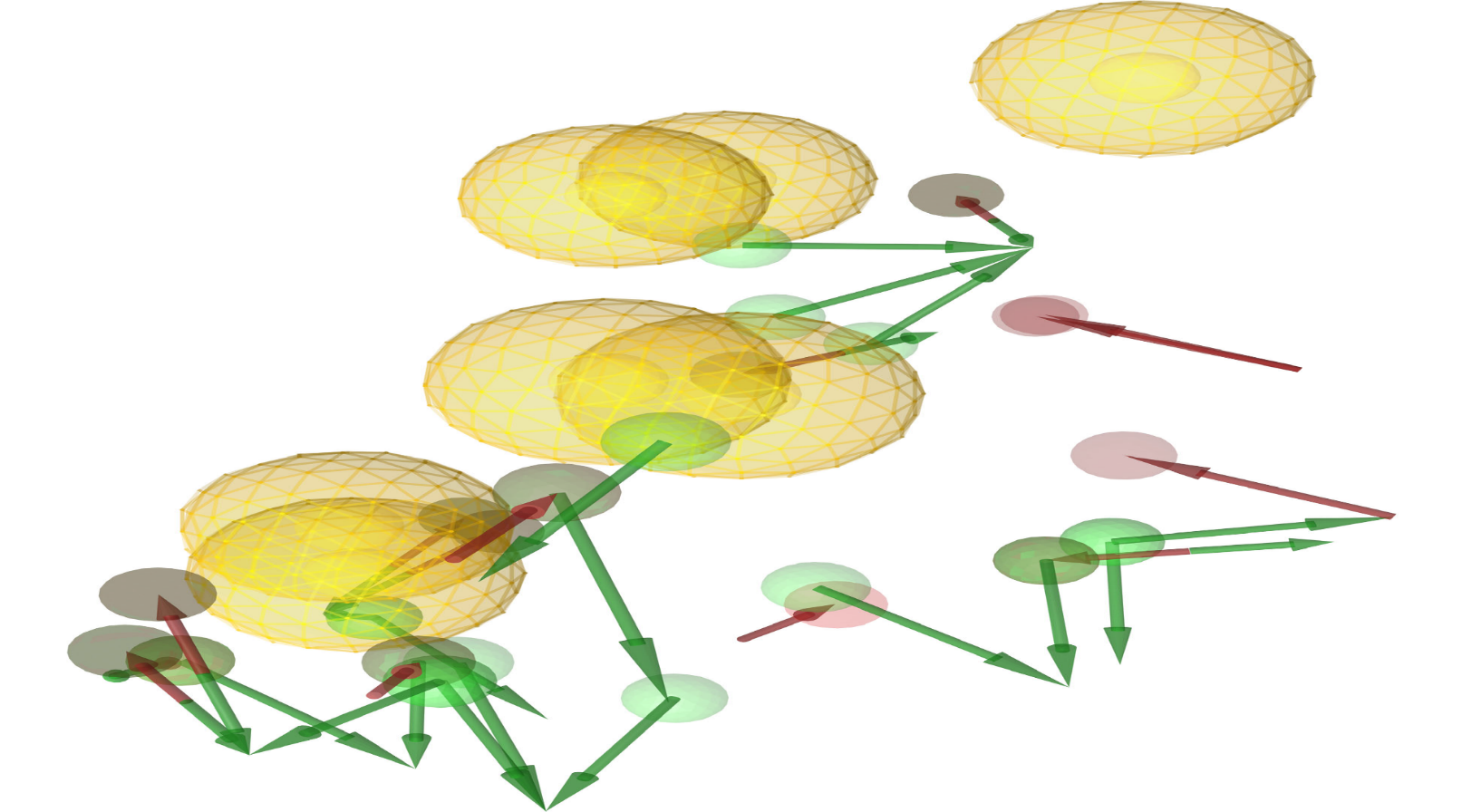 |

Figure S3. A; pharmacophore modeling among conserved residues, B; alignment among modeled pharmacophores to find common pharmacophore.

| 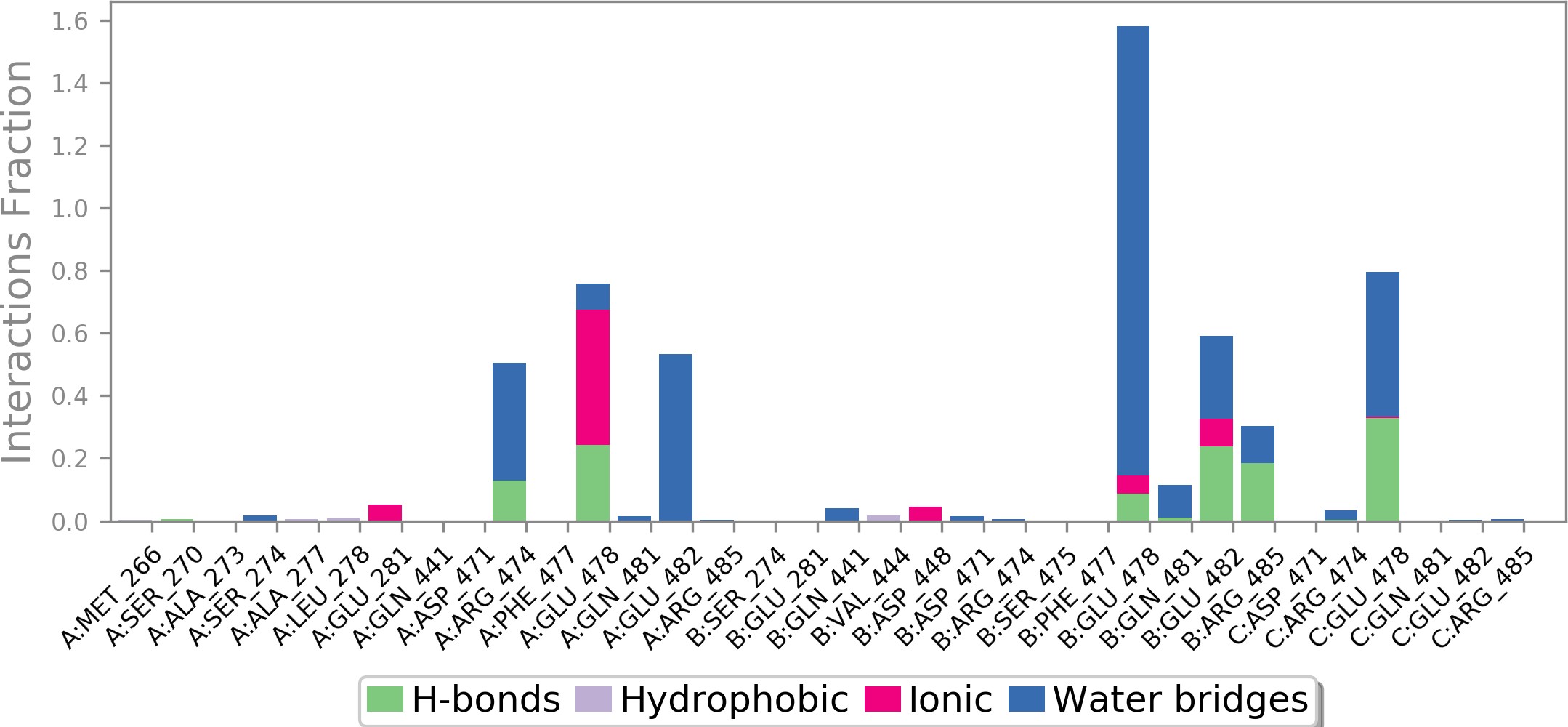 |
| --- |
| 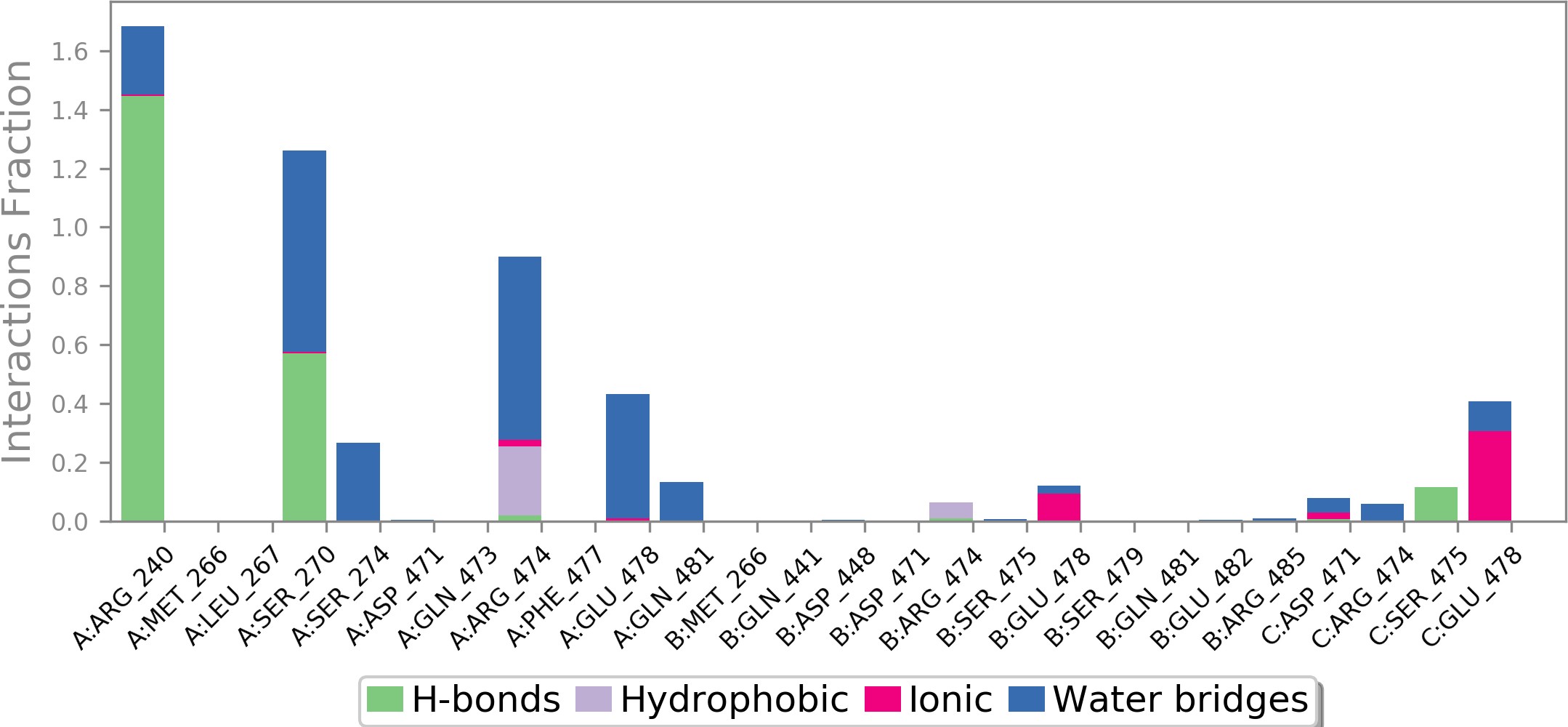 |
| 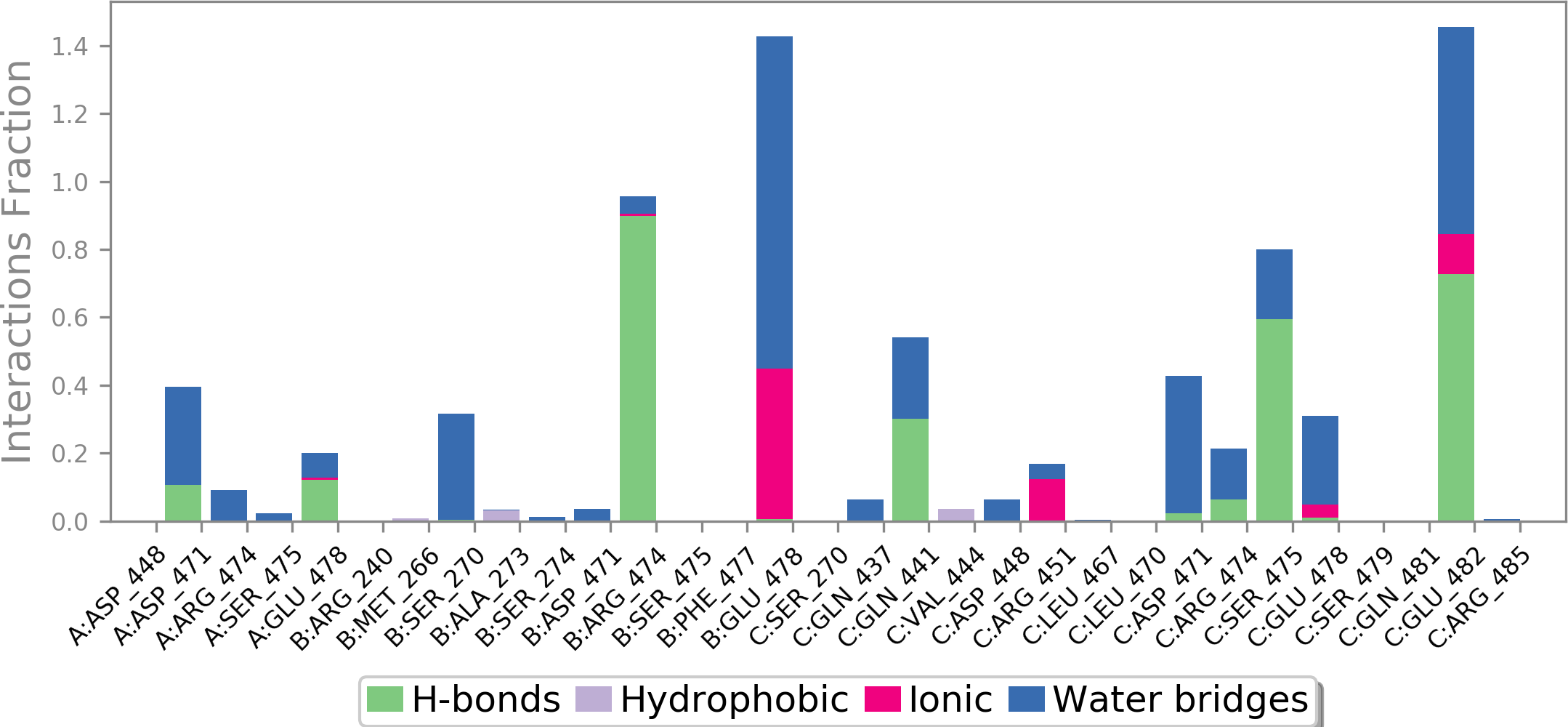 |
| 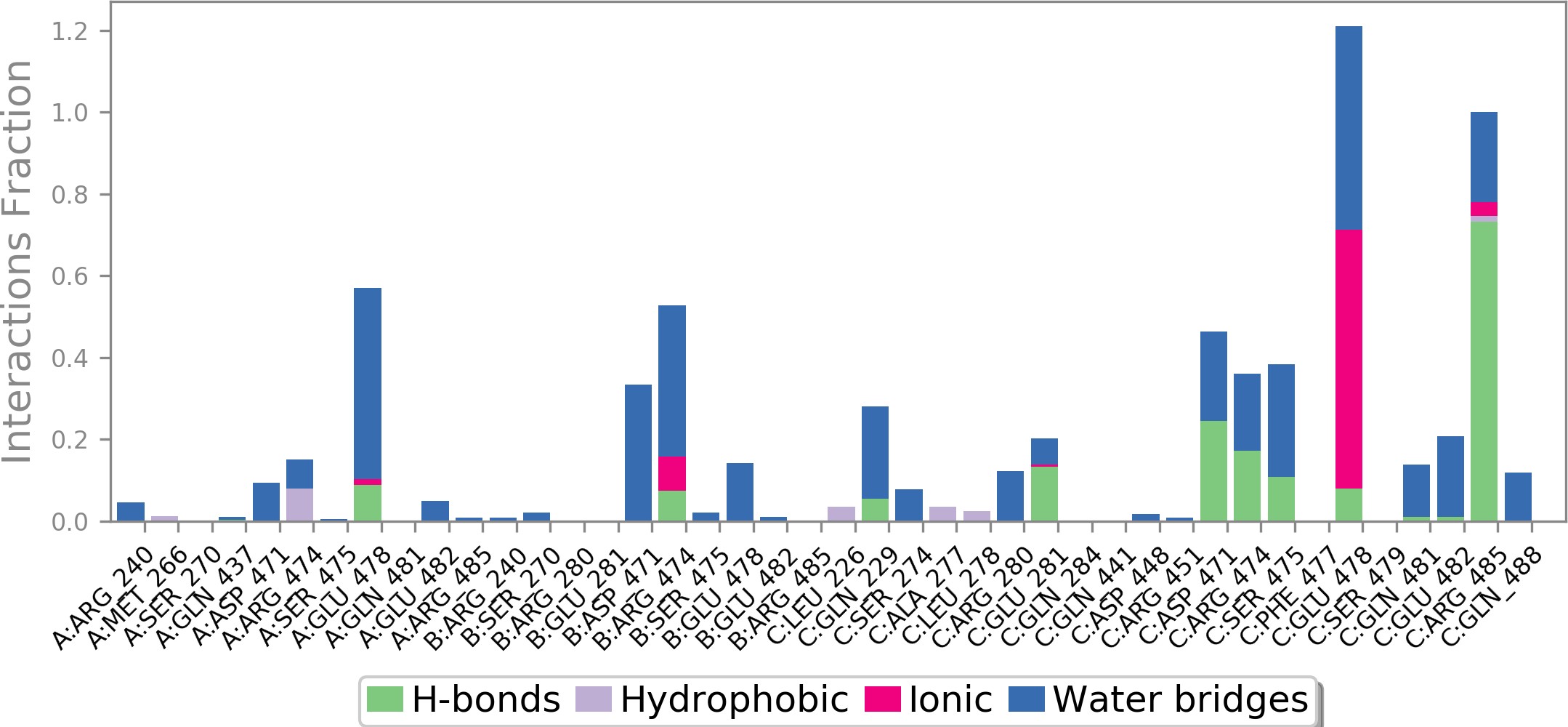 |
| 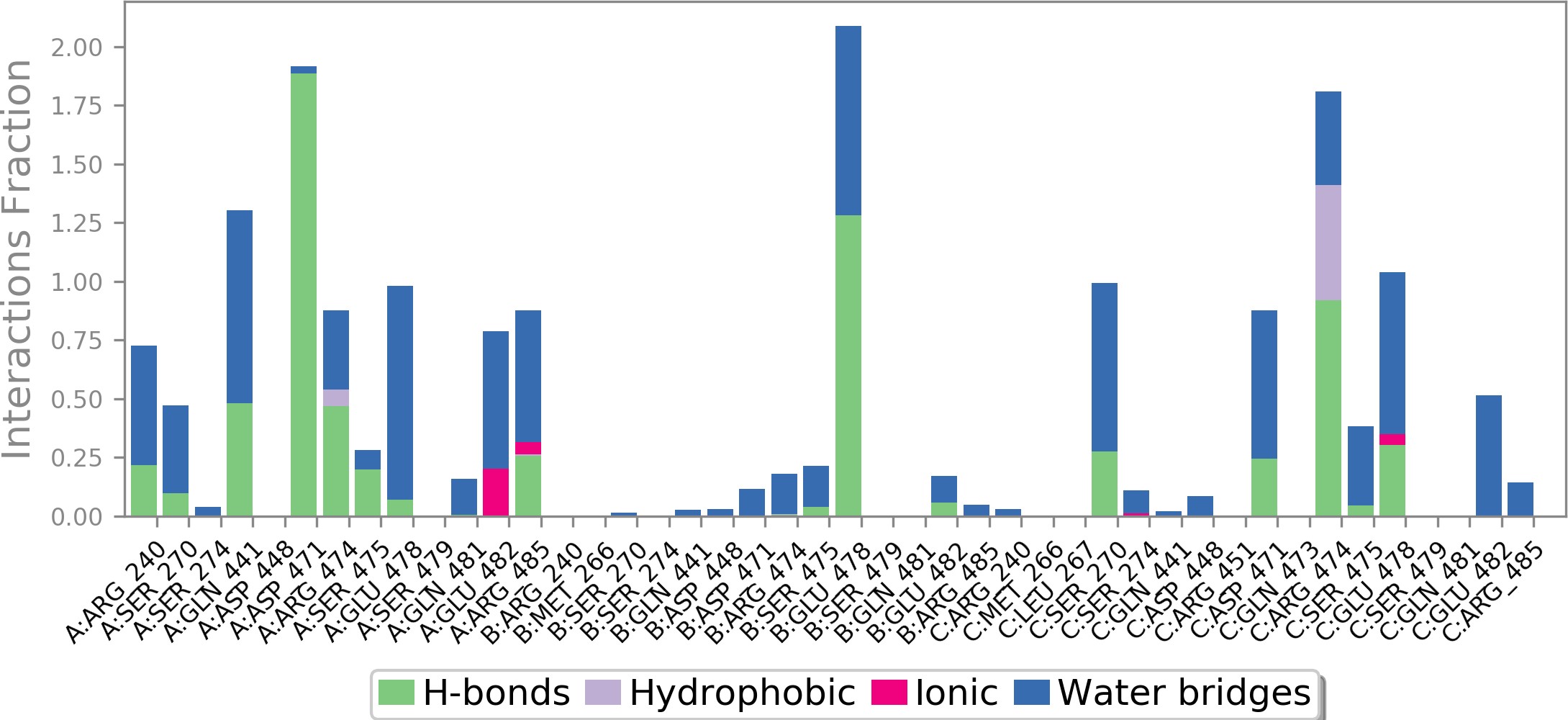 |

Figure S4. protein-ligand interaction of OprA-antibiotics.

| 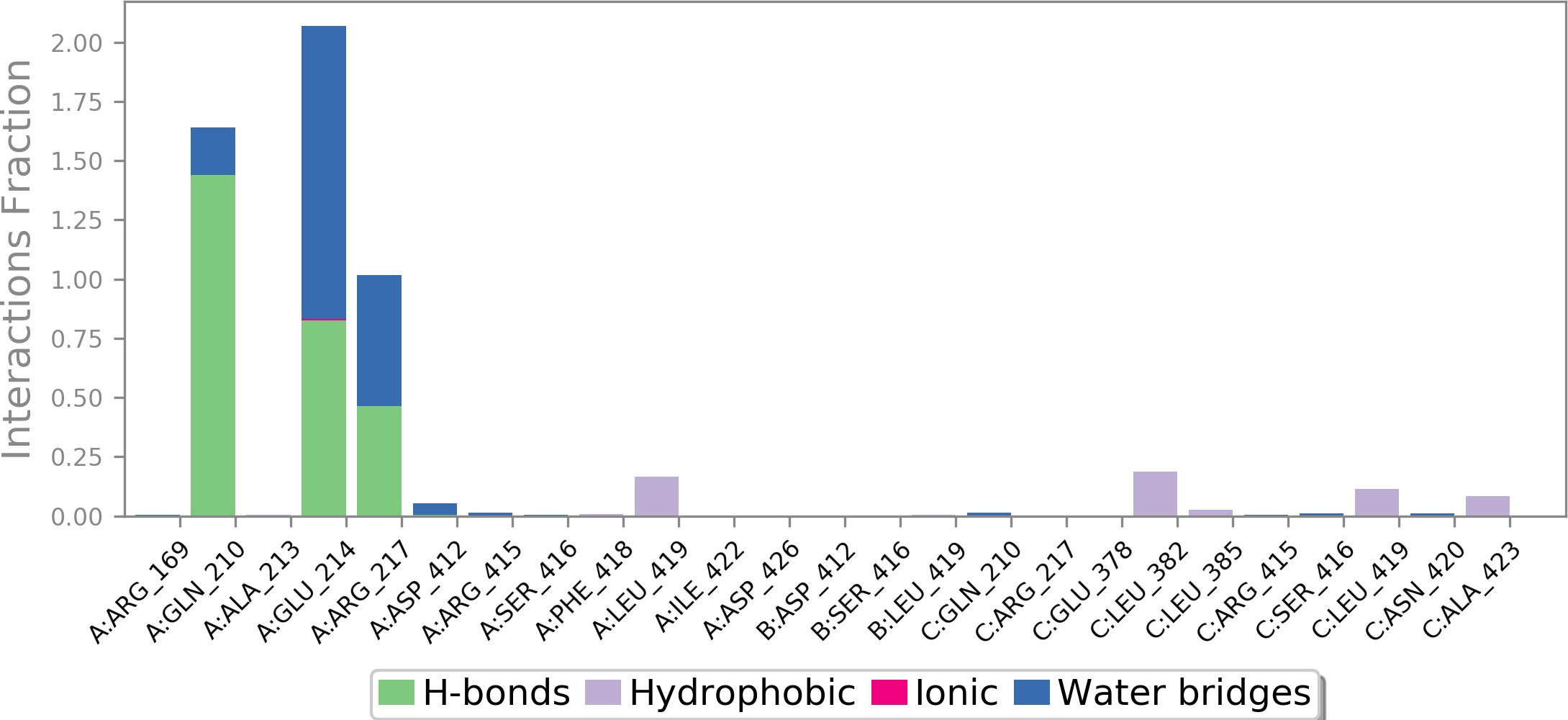 |
| --- |
| 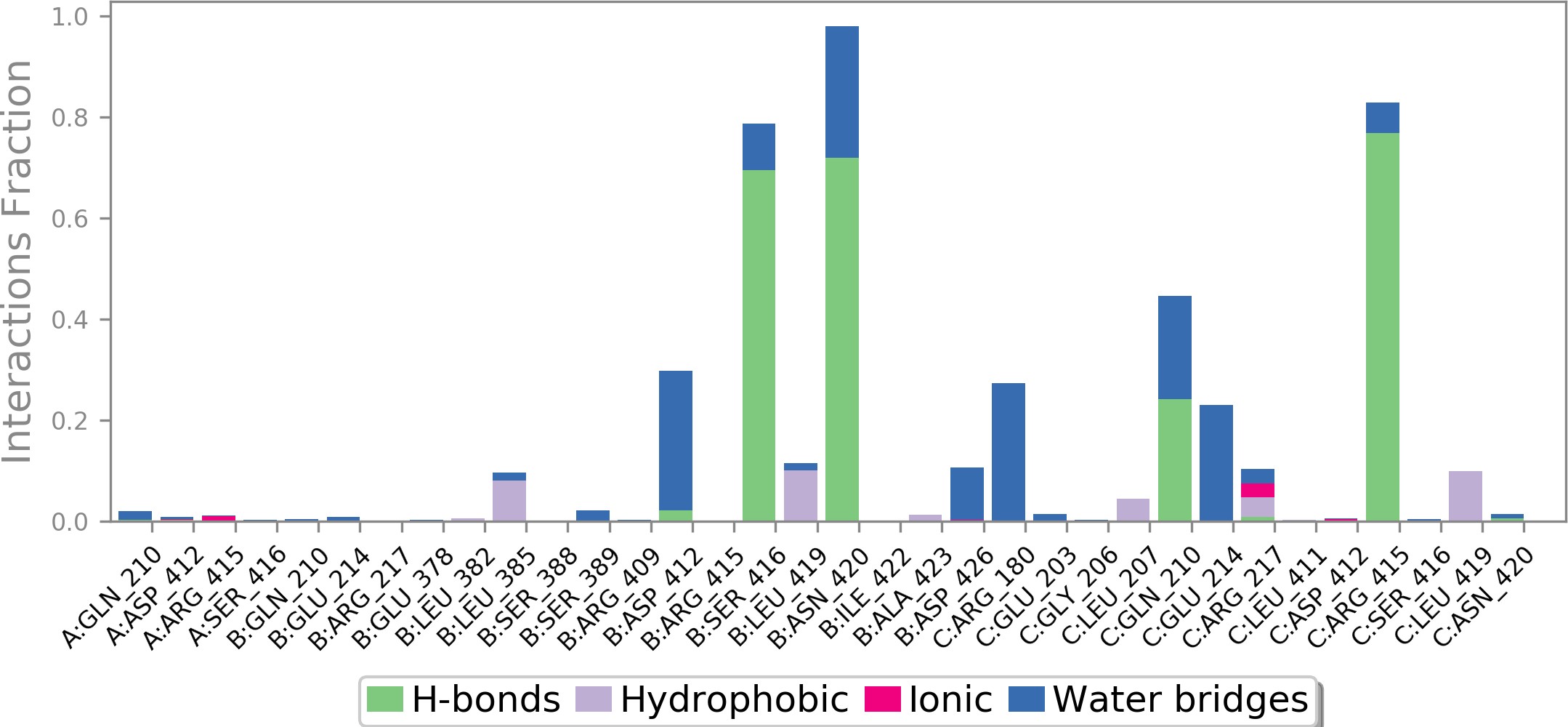 |
| 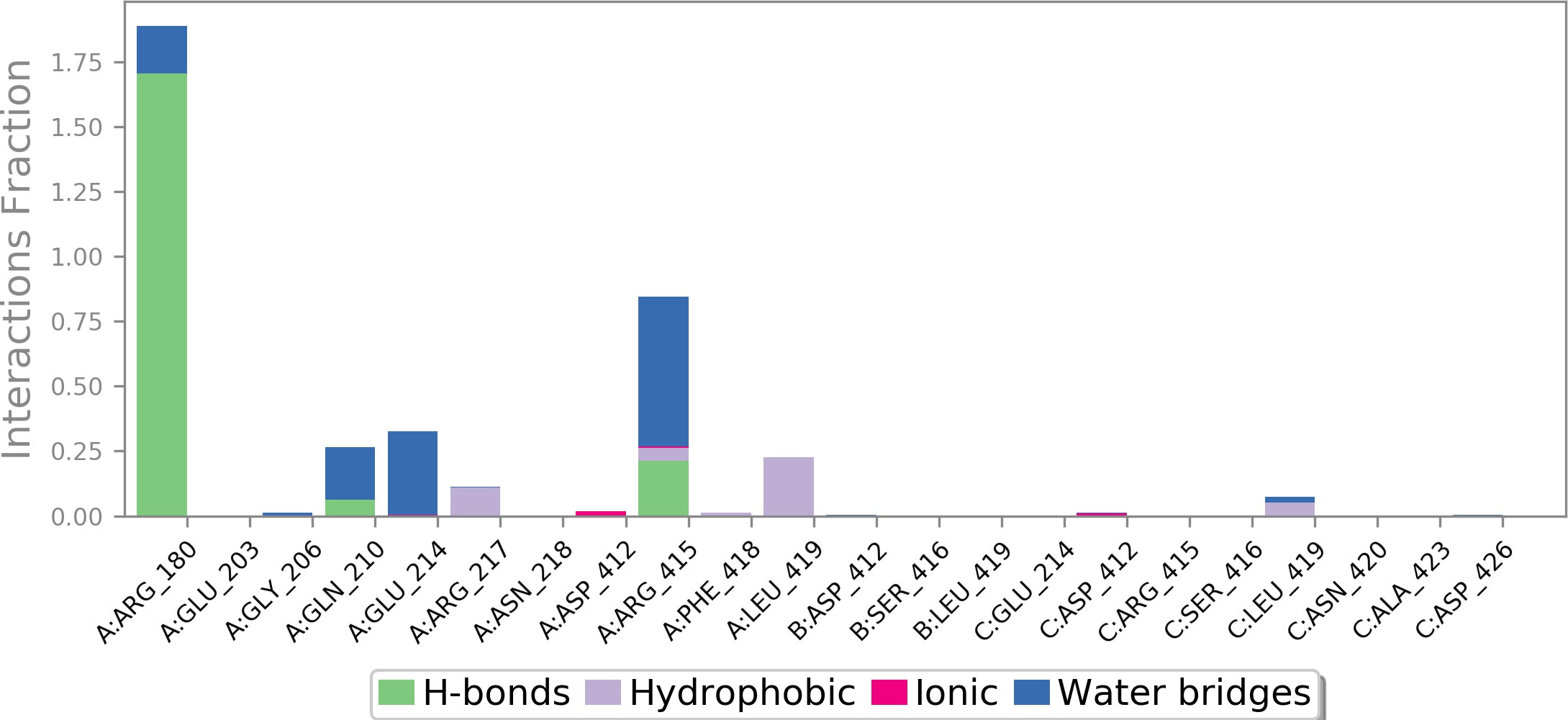 |
| 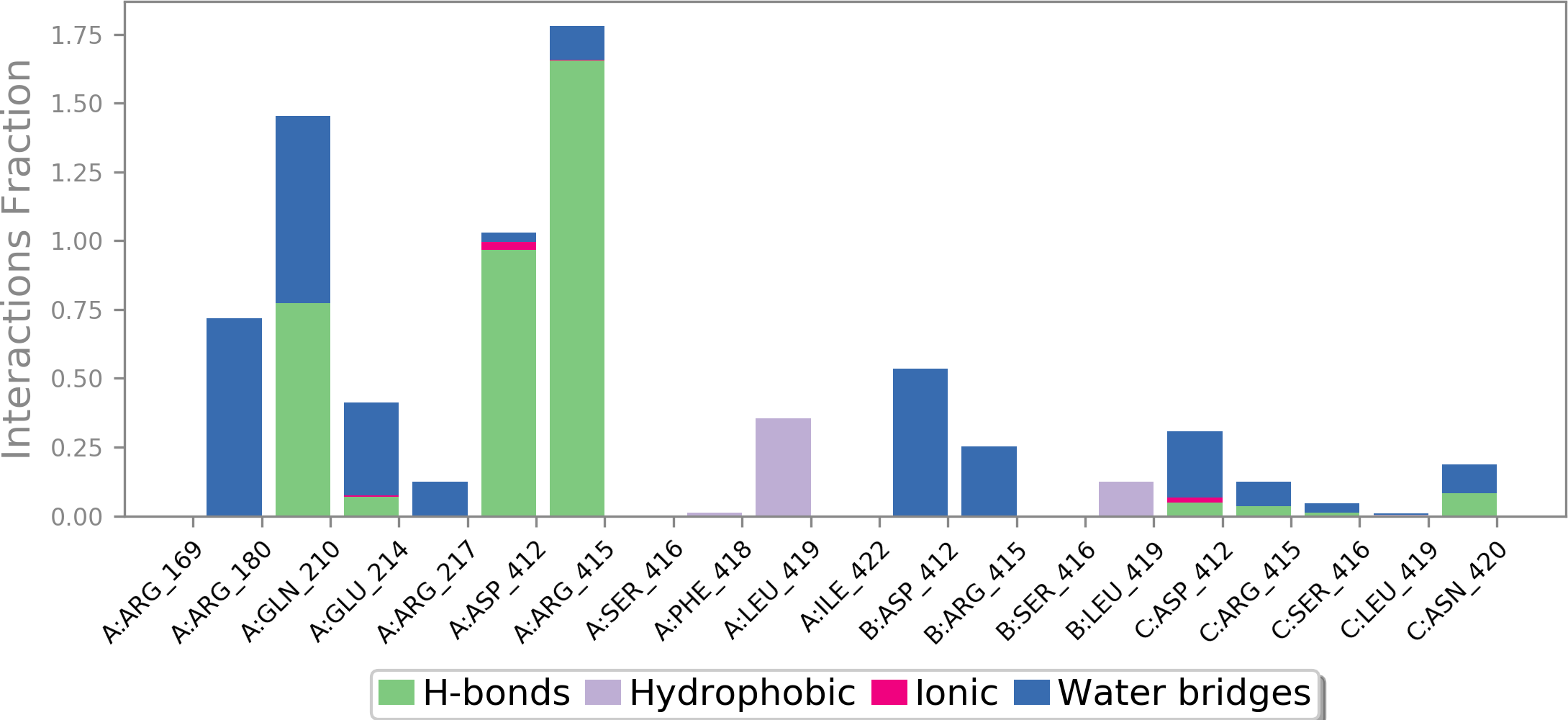 |
| 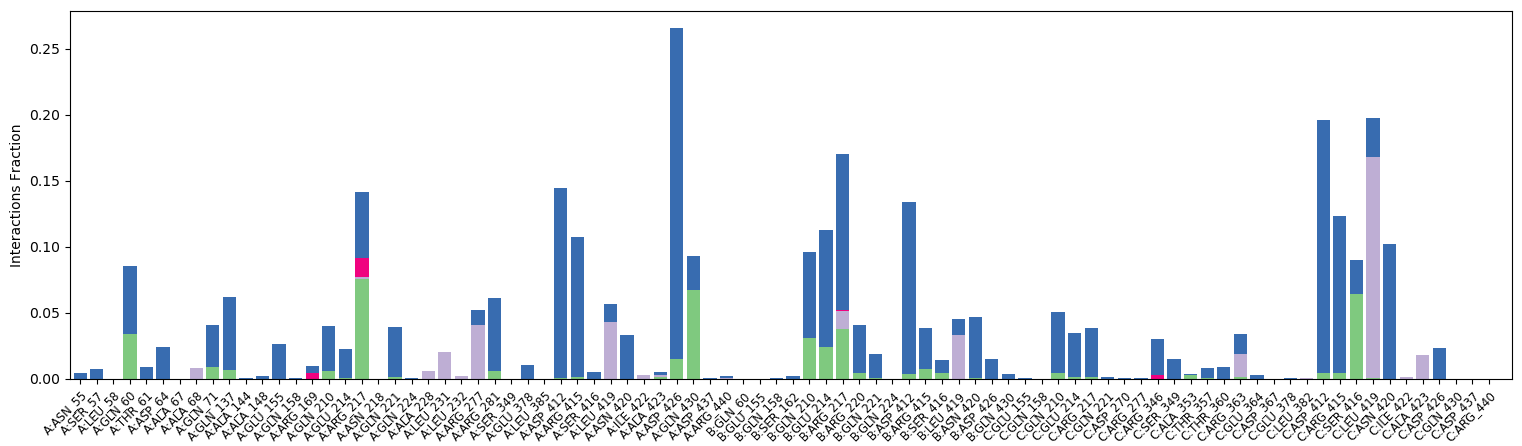 |
| 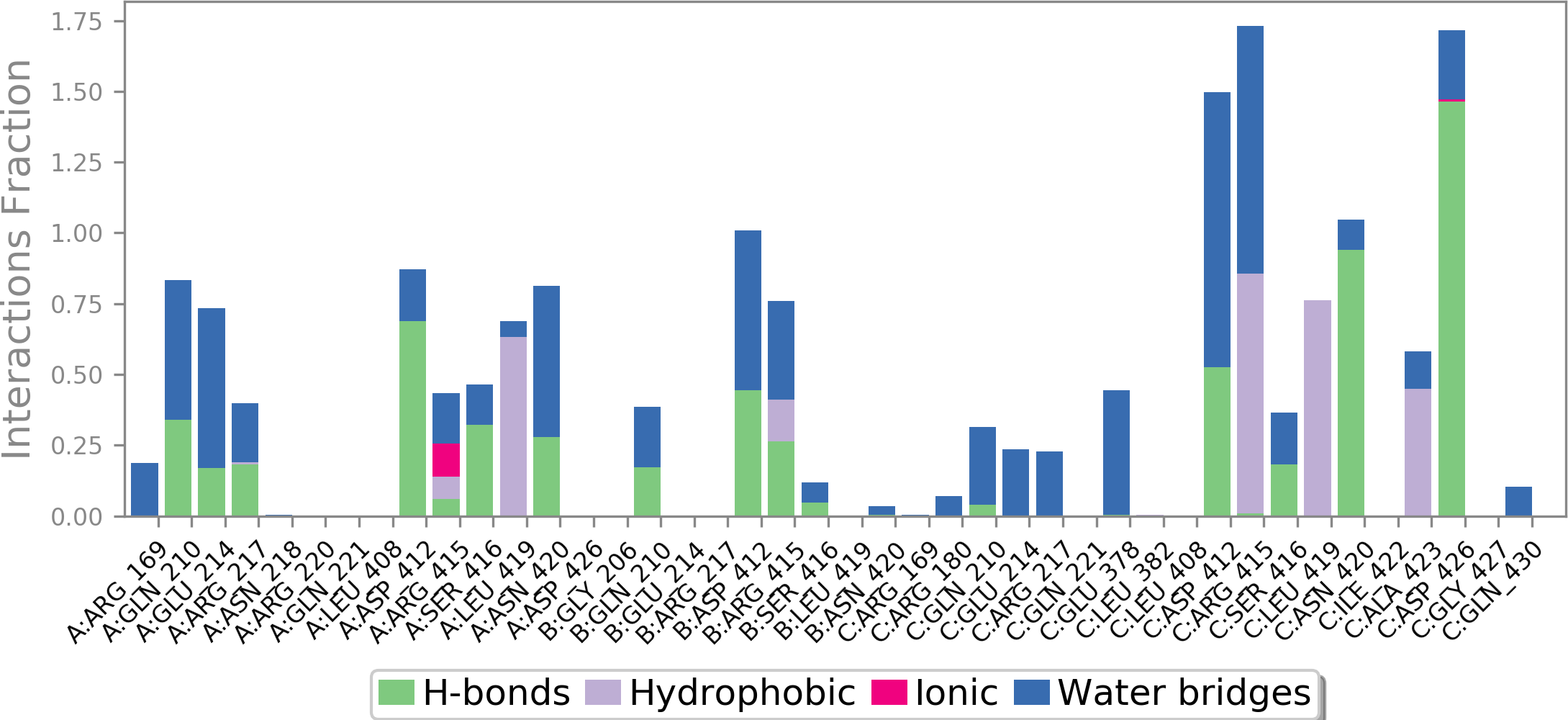 |

Figure S5. protein-ligand interaction of OprJ-antibiotic.

| 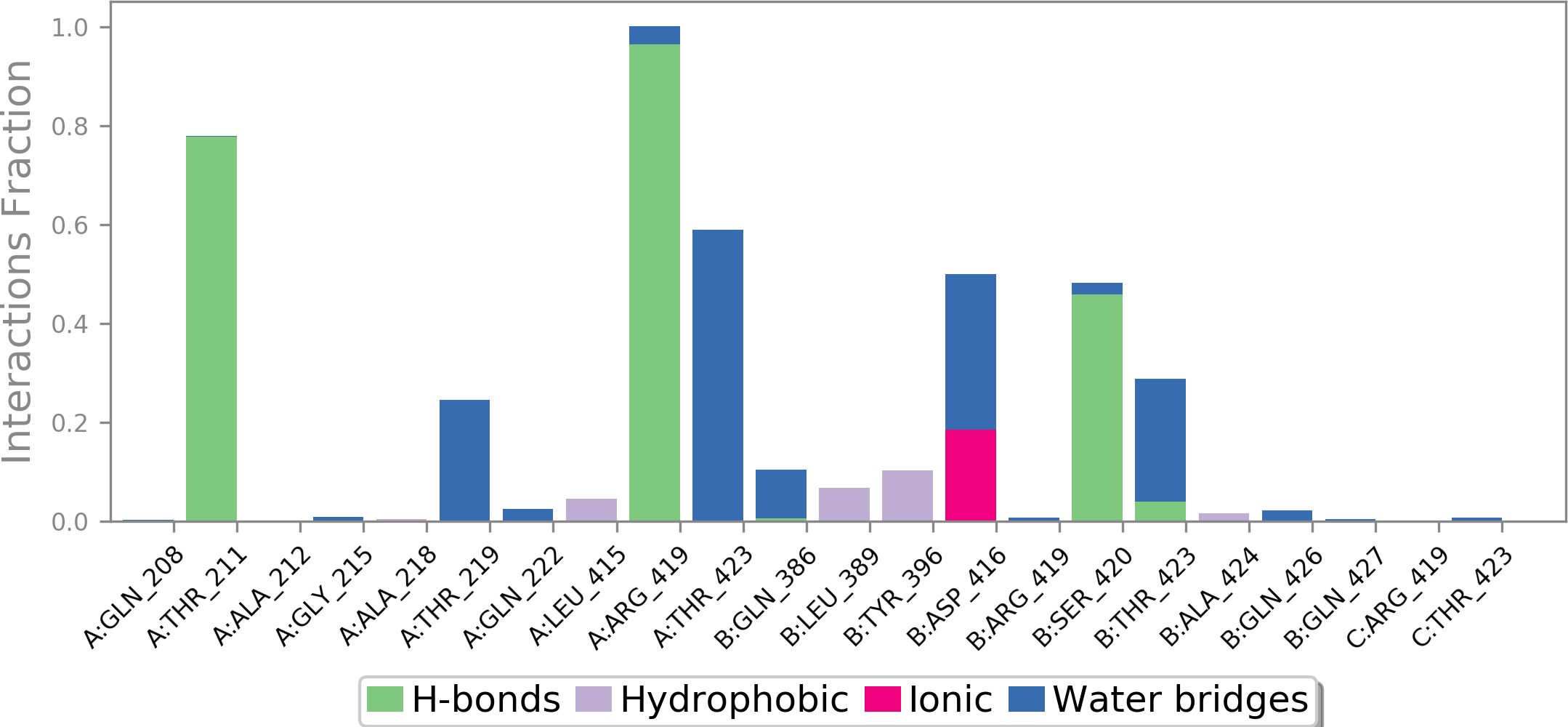 |
| --- |
| 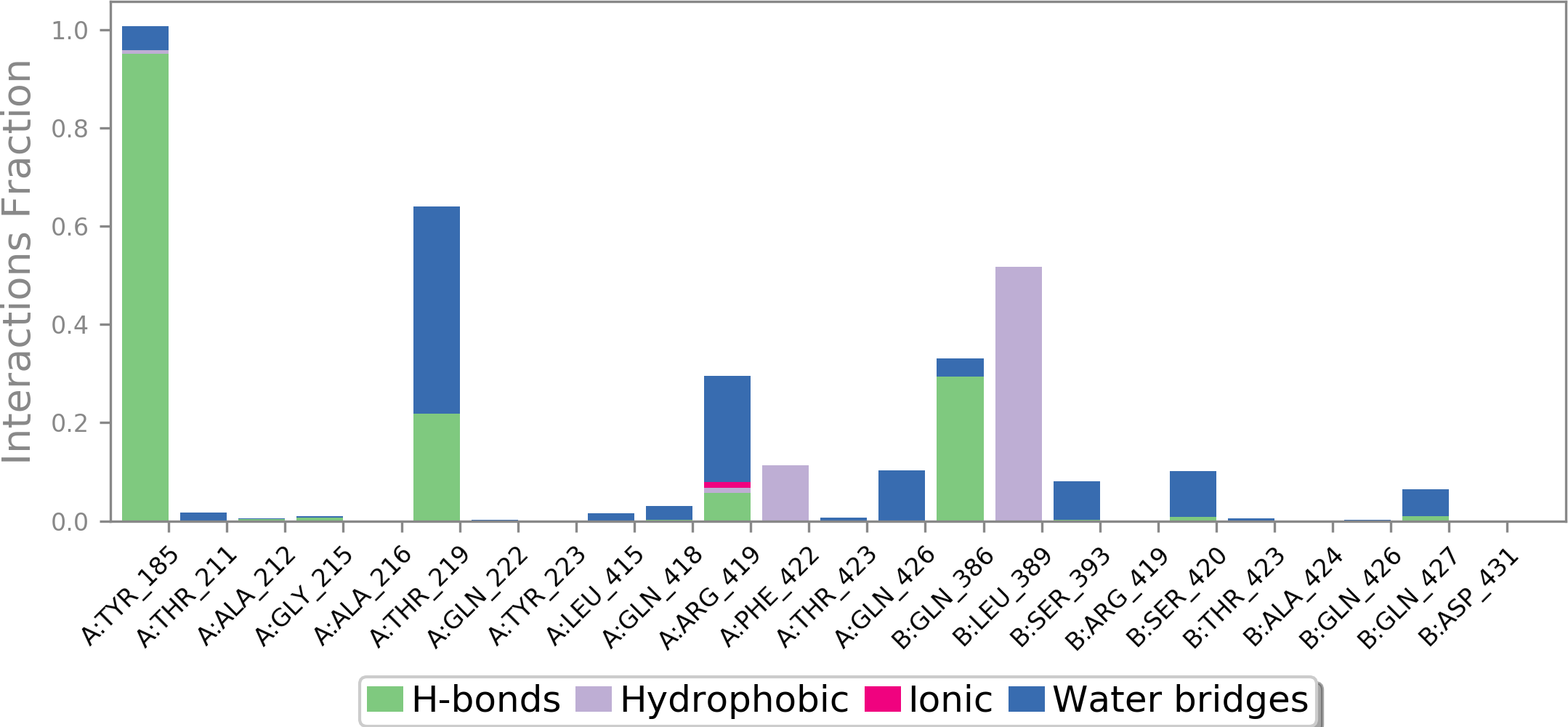 |
| 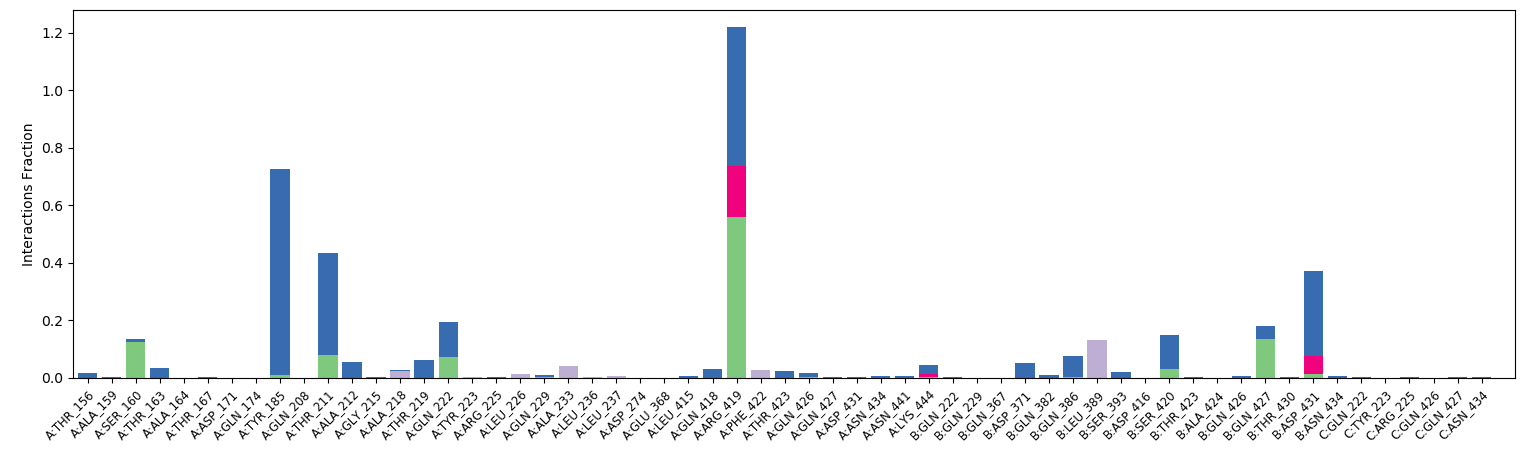 |
| 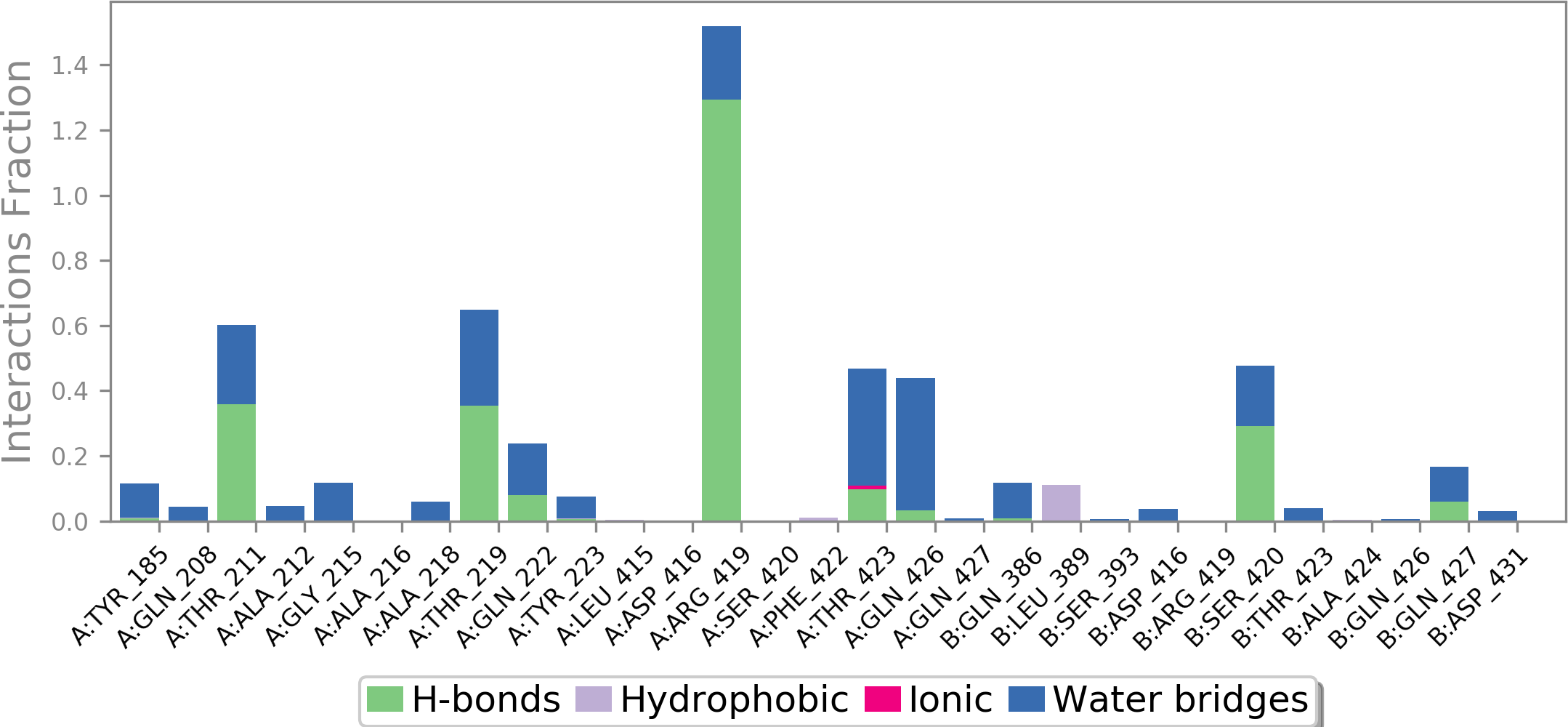 |
| 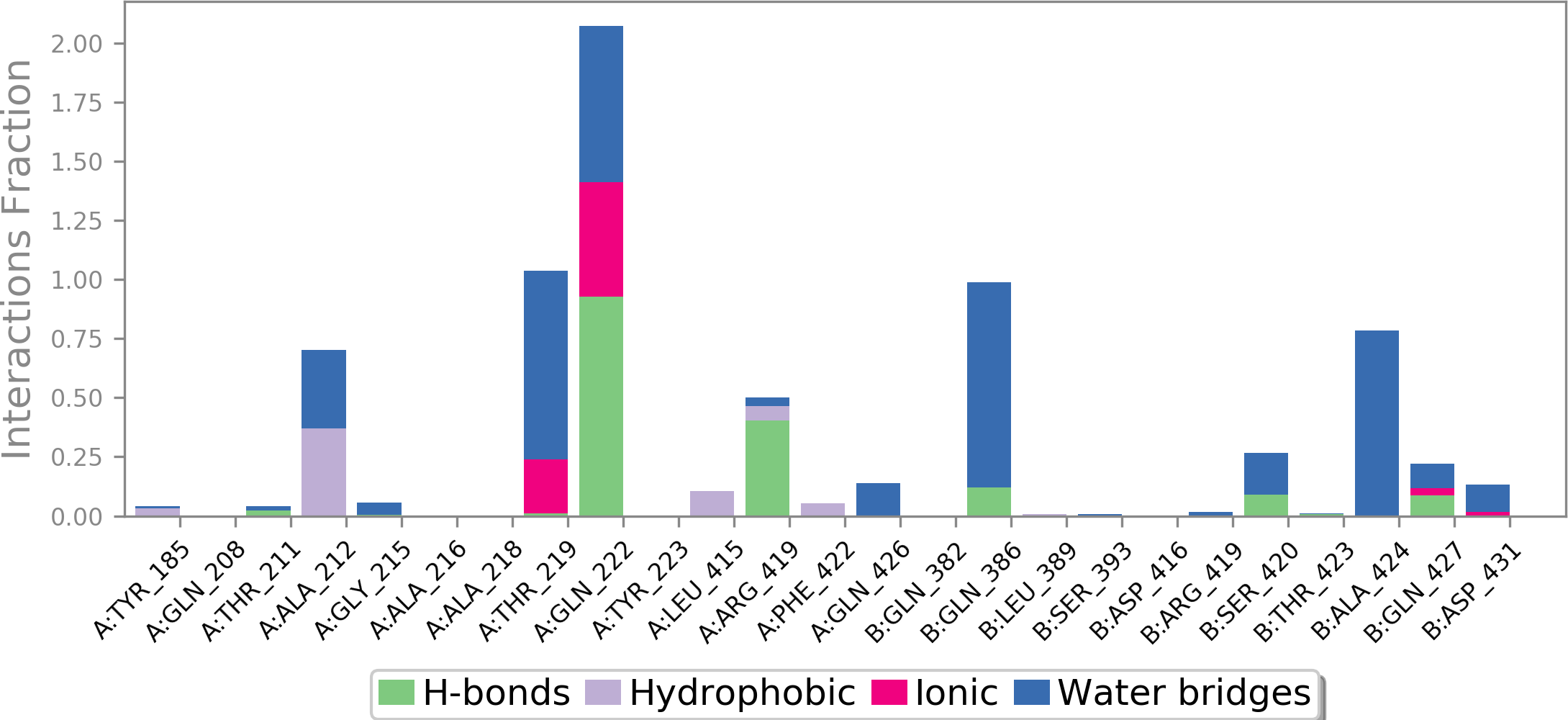 |
| 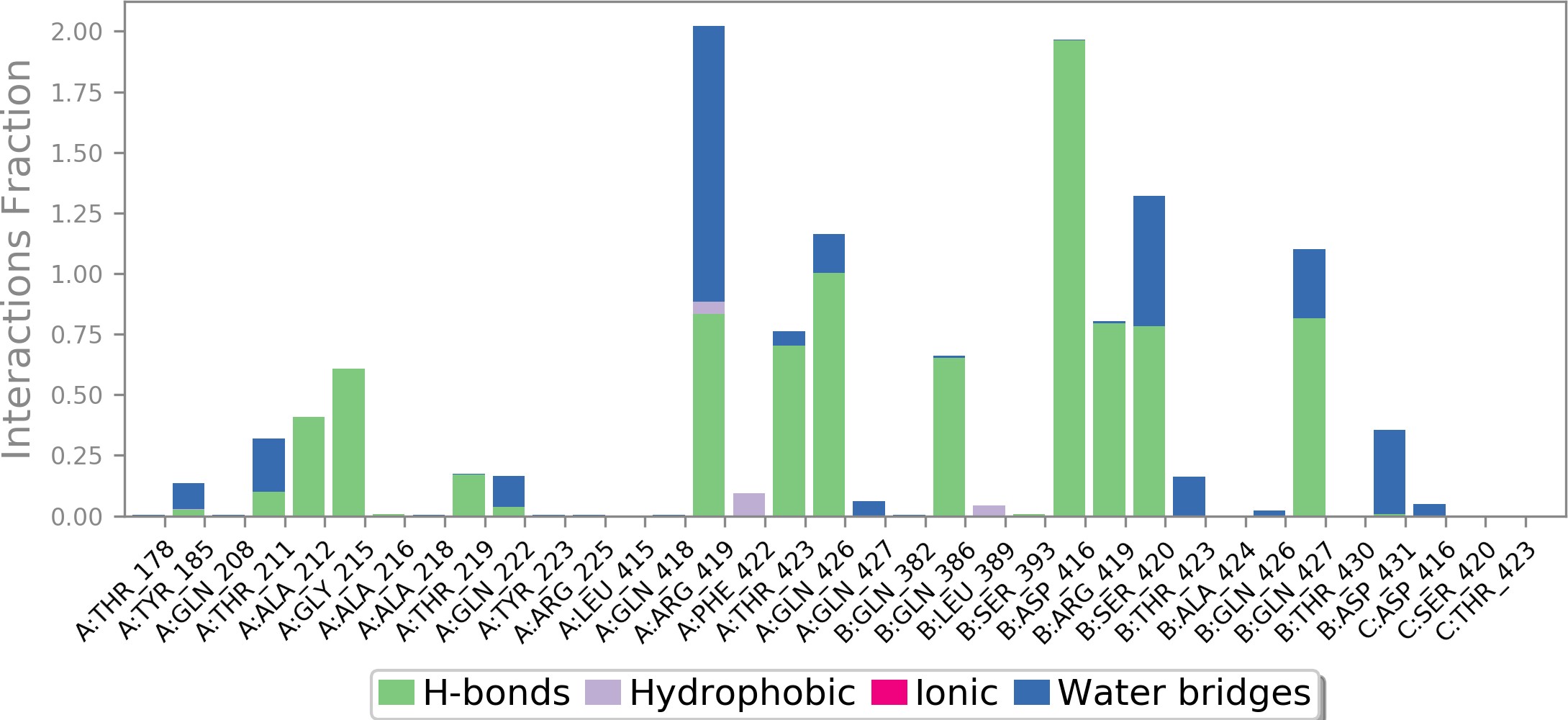 |

Figure S6. protein-ligand interaction of OprM-antibiotic.

| 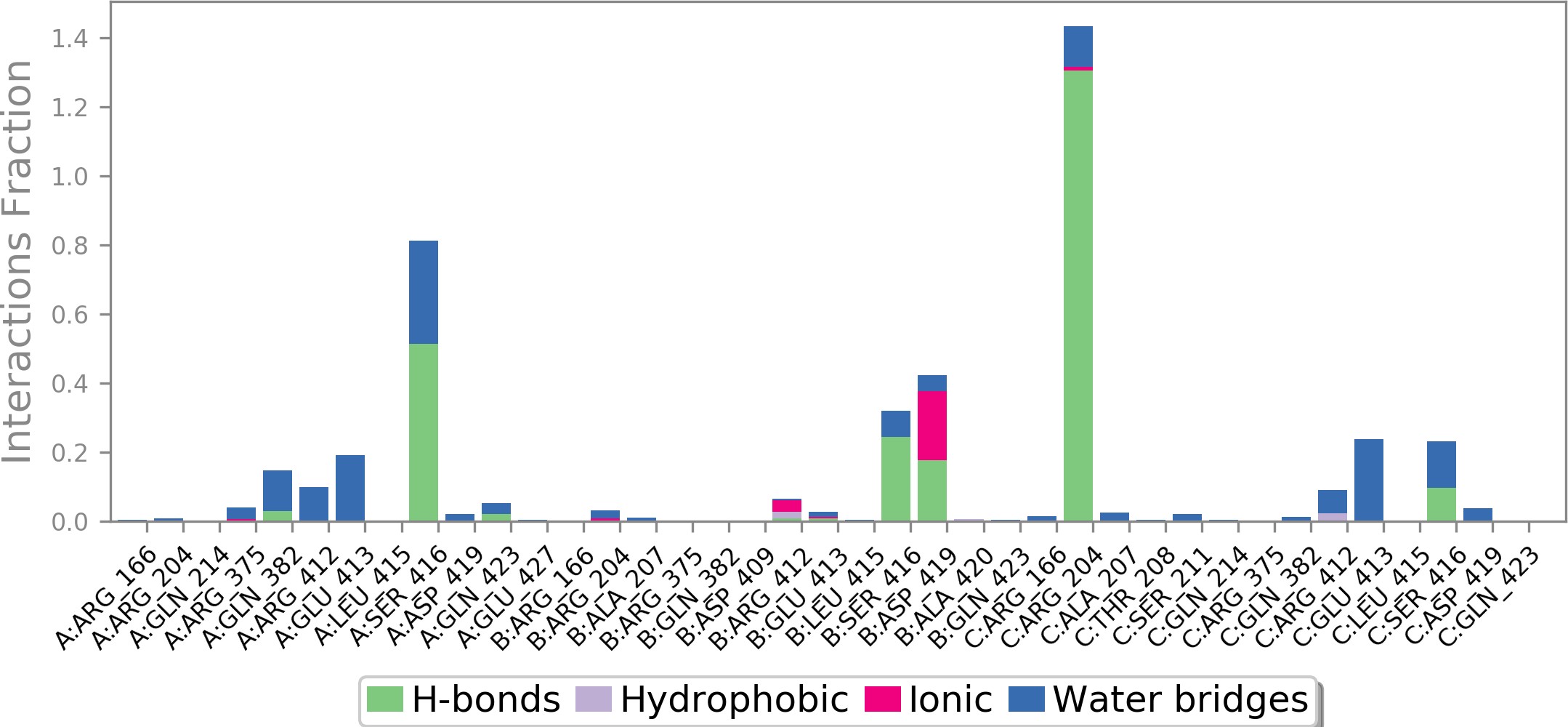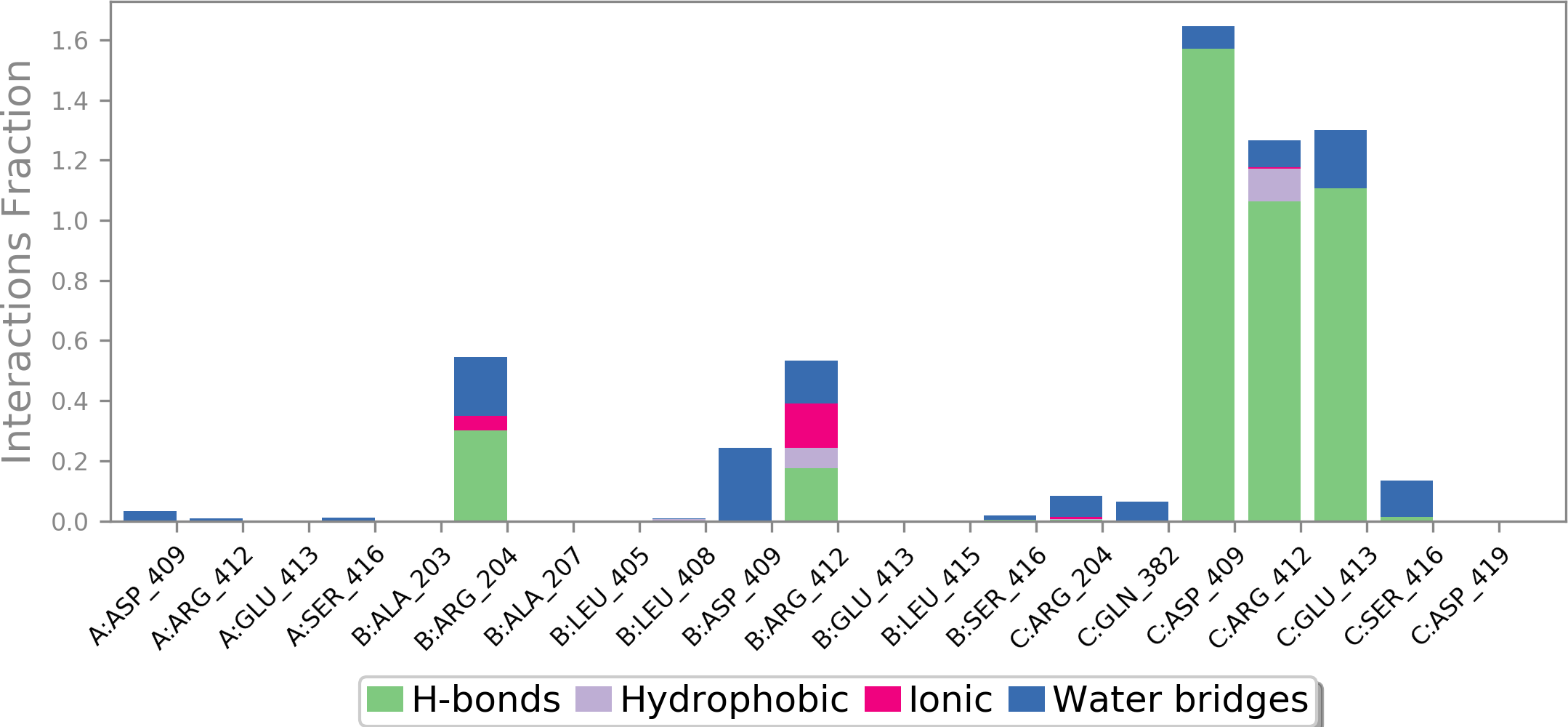 |
| --- |
| 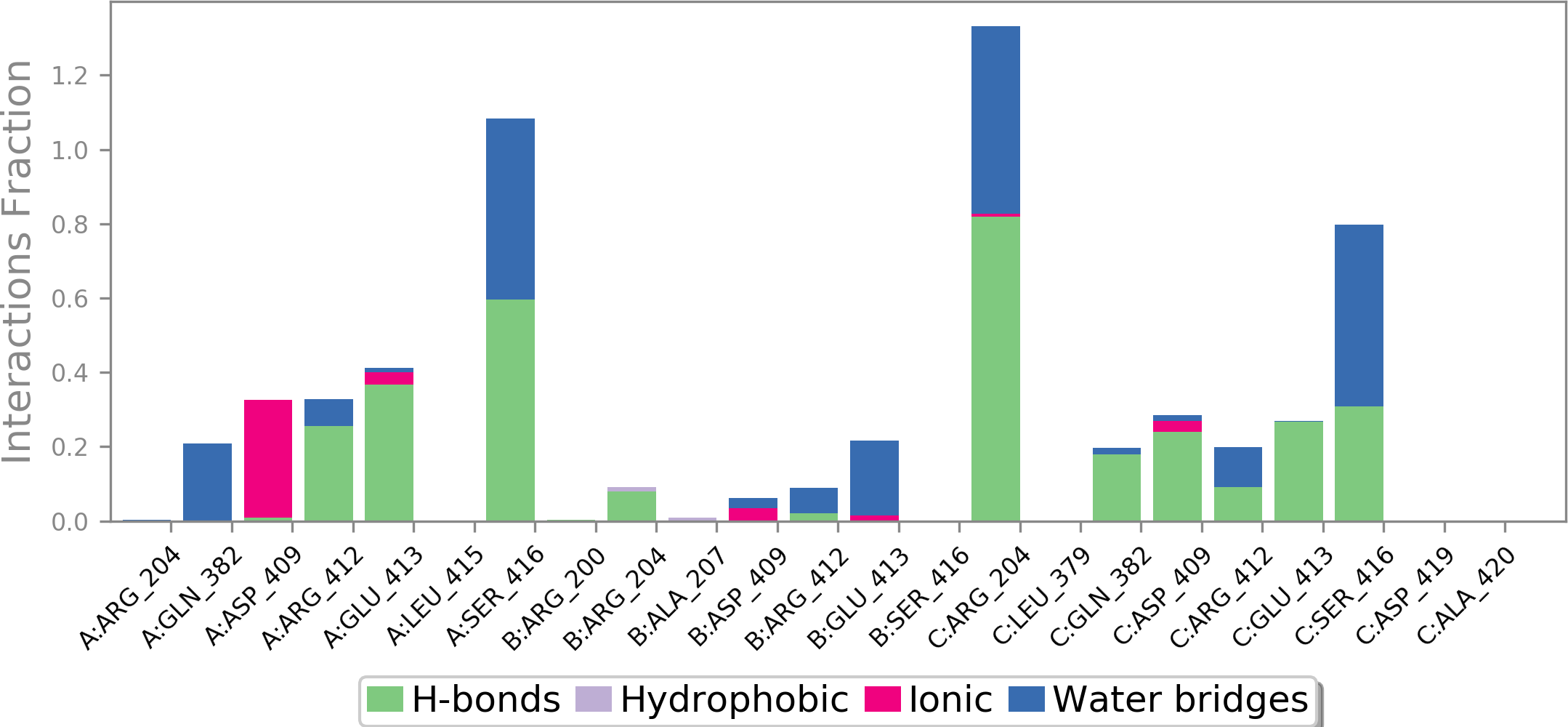 |
| 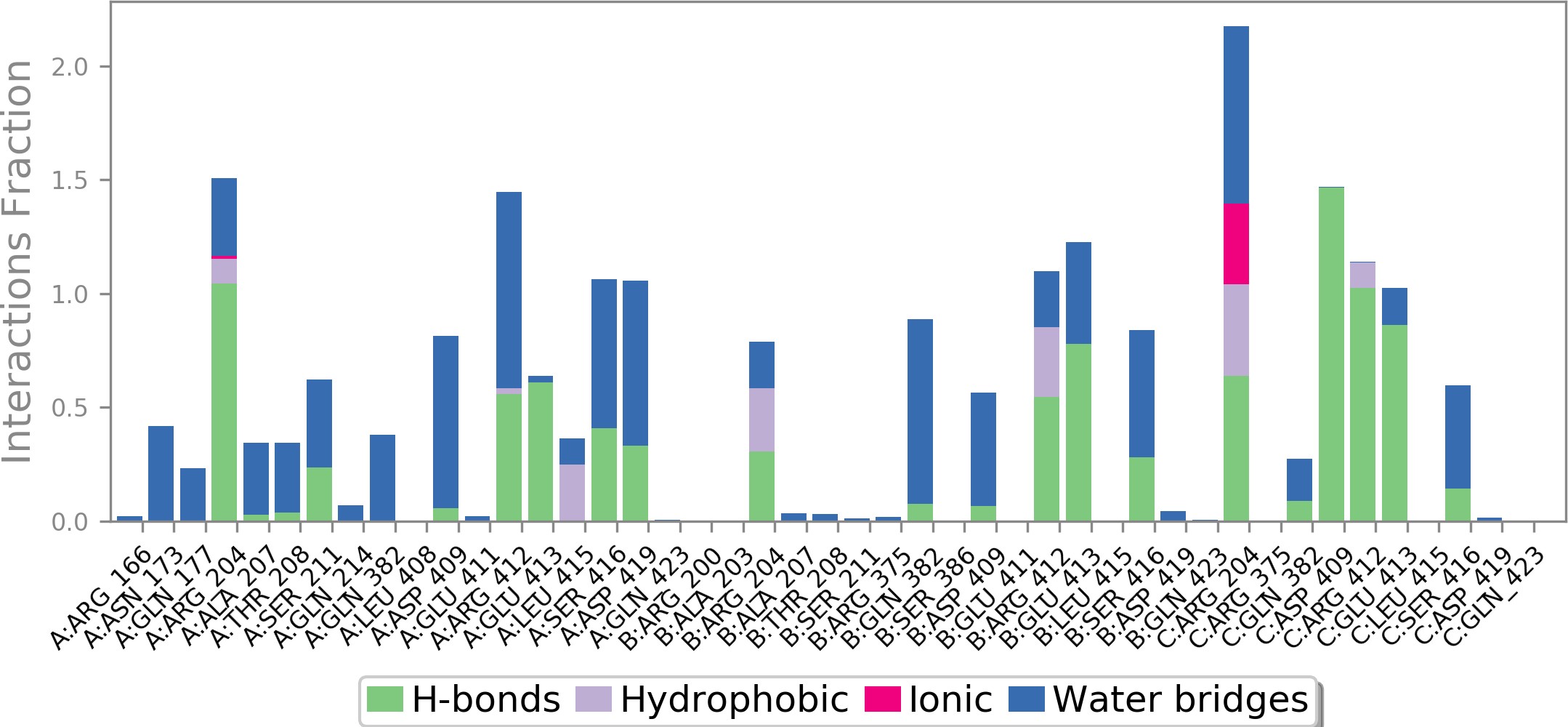 |

Figure S7. protein-ligand interaction of OprN-antibiotic.

| 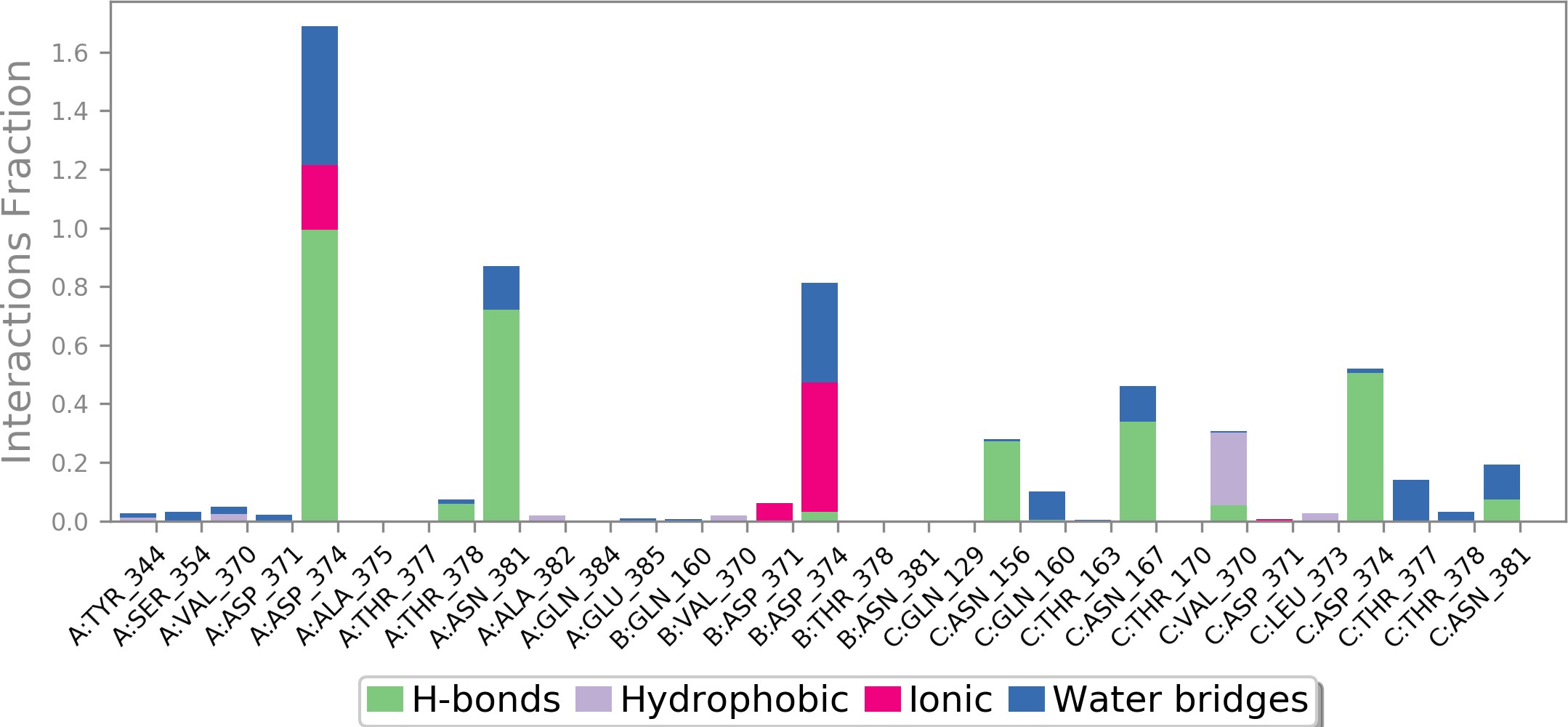 |
| --- |
| 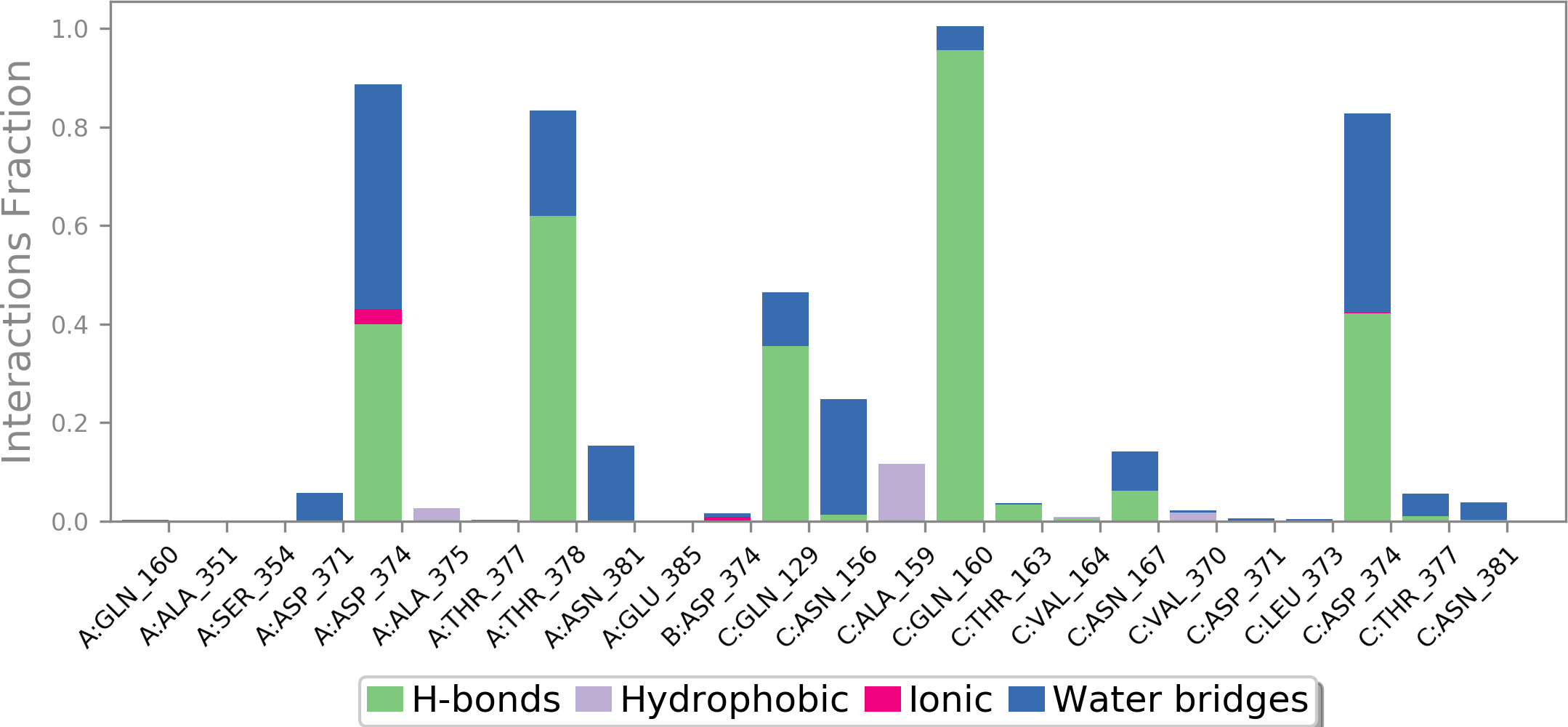 |
| 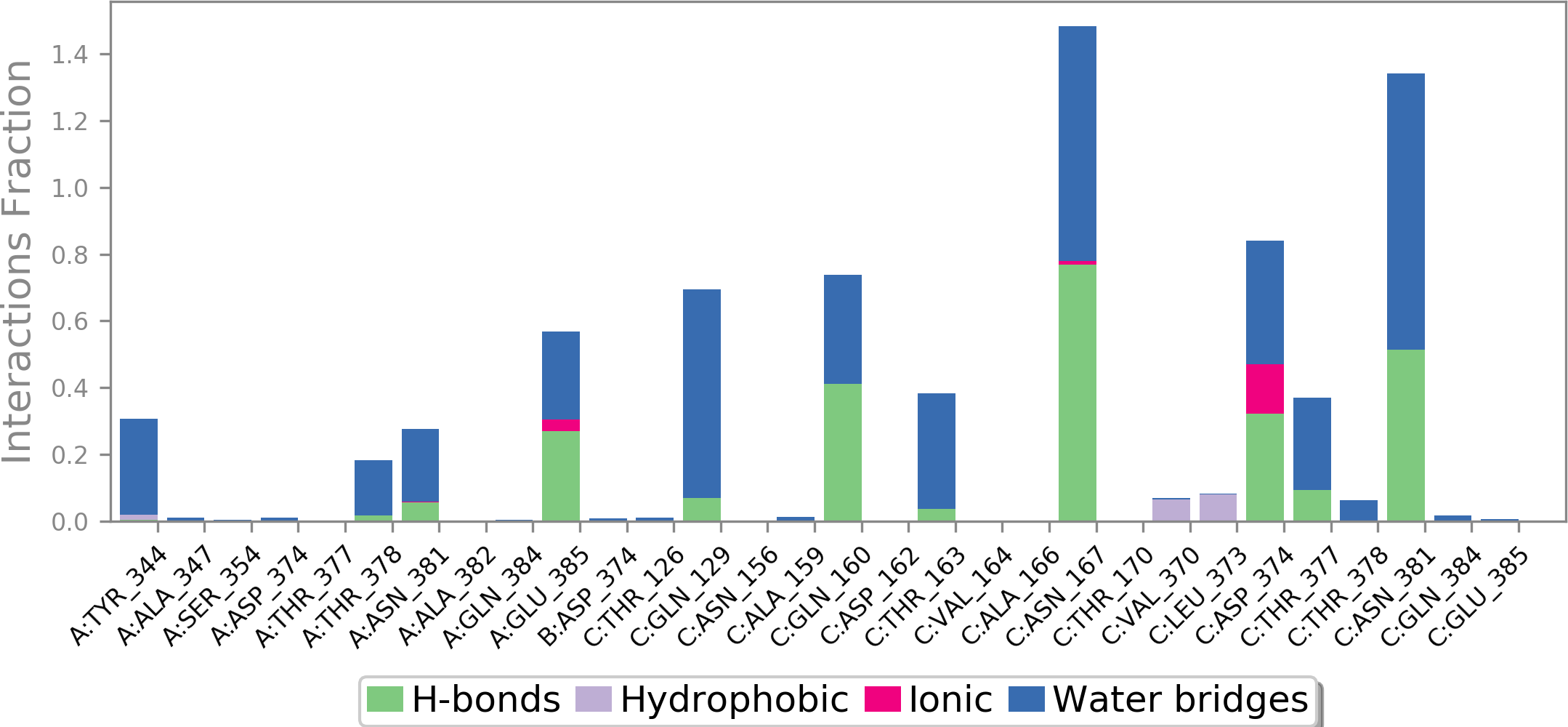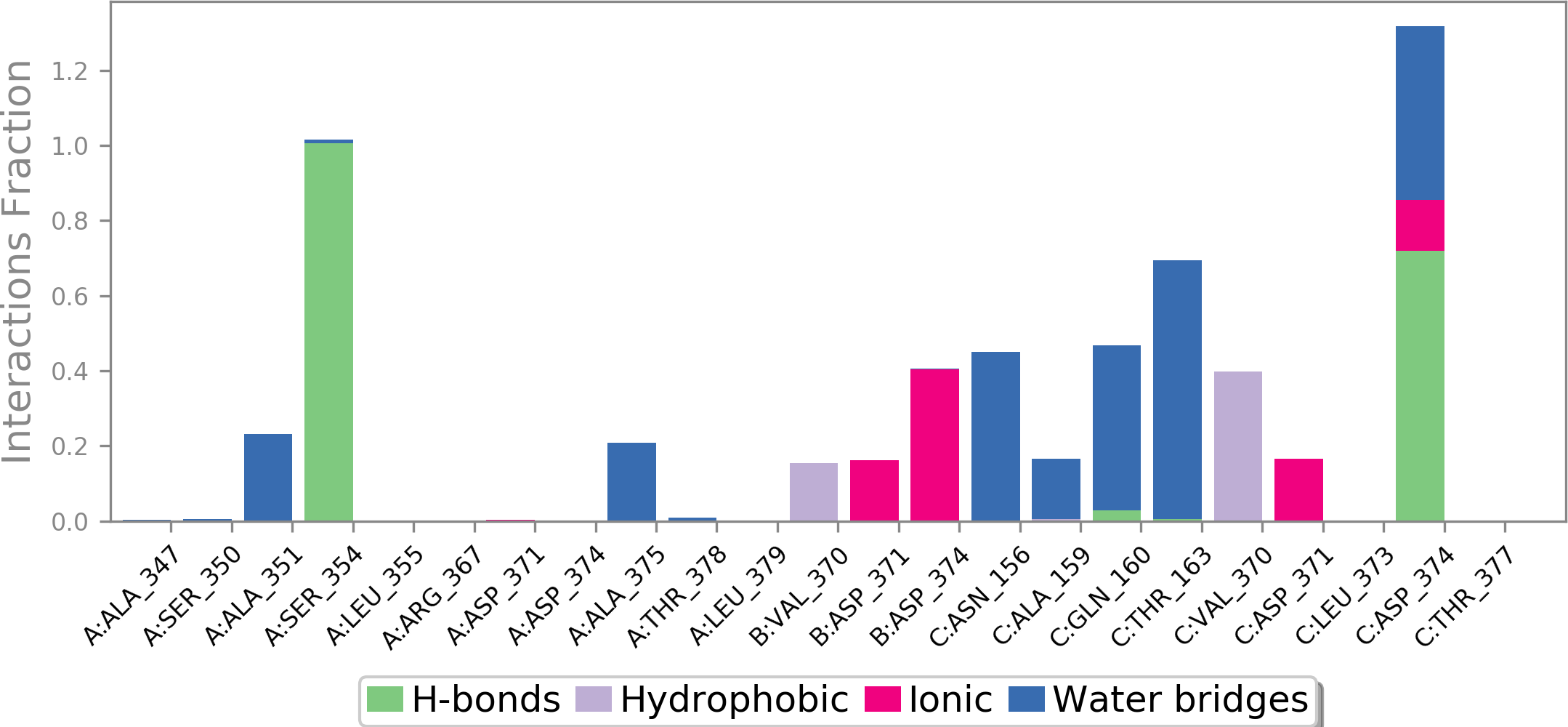 |
| 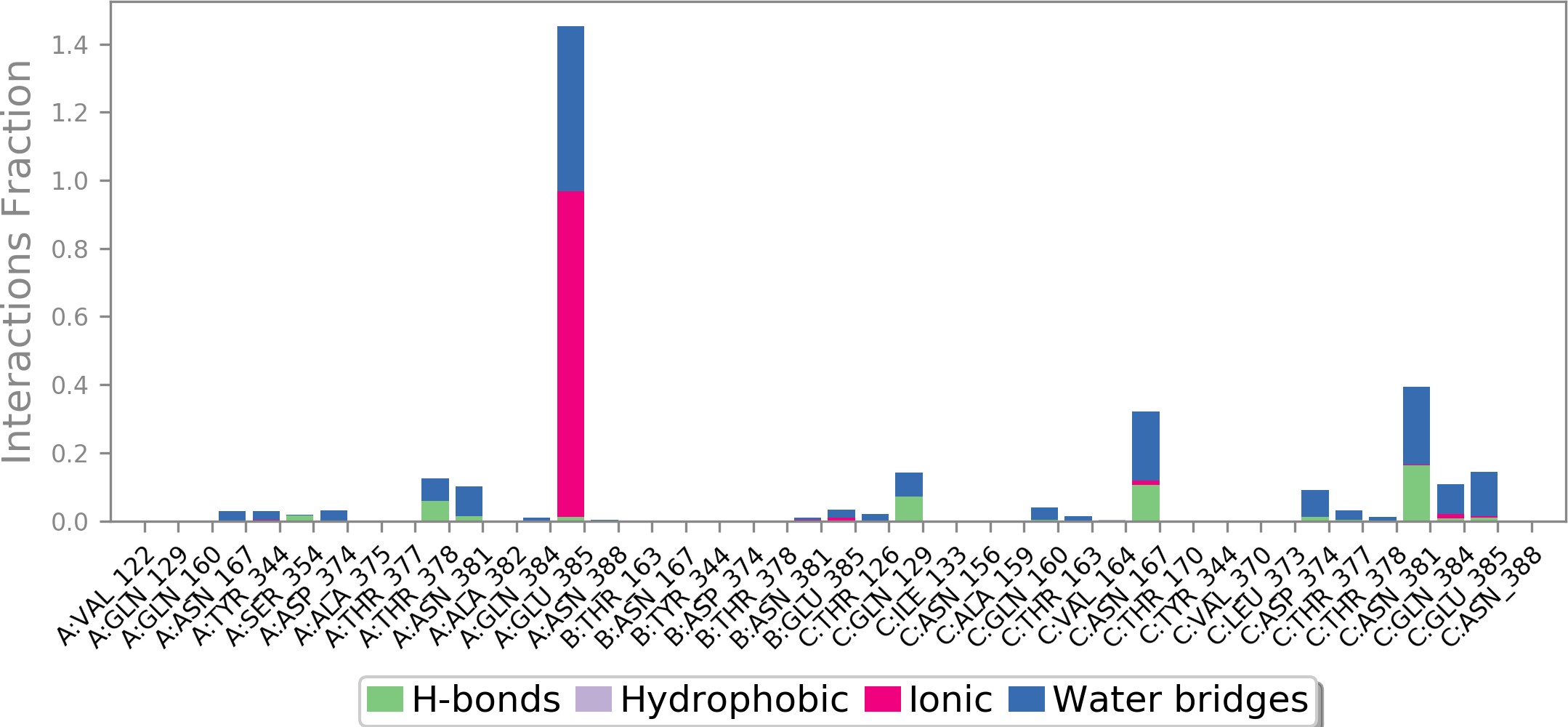 |
| 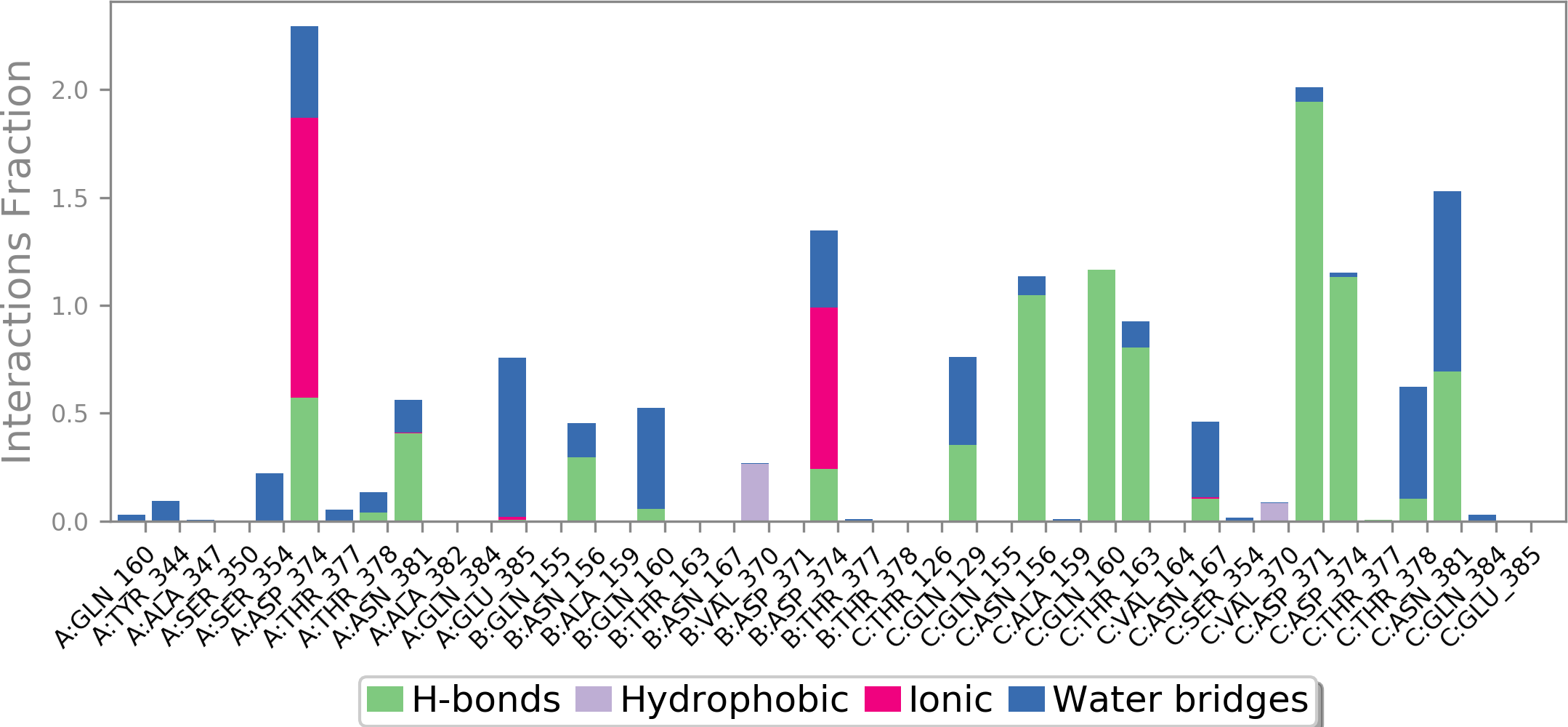 |

Figure S8. protein-ligand interaction of TolC-antibiotic.

|  |
| --- |
|  |
|  |
|  |
|  |

Figure S9. RMSD analyses of antibiotics and NPC100251 in complex with OMPs. (Only the first five digit of compounds name were mentioned).

|  |
| --- |
|  |
|  |
|  |
|  |

Figure S10. RMSF analyses of all complexes during the 20 ns of MD simulation.

Table S1. The average of proteins backbone RMSD during the last 10 ns of MD simulation (only the first five digit of compounds name were mentioned.

|  | azithro | chloramph | levoflo | merop | NPC100251 | PRO | tetra |
| --- | --- | --- | --- | --- | --- | --- | --- |
| OprA Back-bone RMSD (Å) | 2.65±0.07 | --- | 2.47±0.07 | 2.37±0.07 | 2.22±0.09 | 2.44±0.11 | 2.48±0.07 |
|  | **azithro** | **chloramph** | **levoflo** | **merop** | **NPC100251** | **PRO** | **tetra** |
| OprJ Back-bone RMSD (Å) | 1.7±0.05 | 2.04±0.09 | 1.83±0.07 | 2.25±0.08 | 1.53±0.1 | 2.003±0.1 | 1.99±0.1 |
|  | **azithro** | **chloramph** | **levoflo** | **merop** | **NPC100251** | **PRO** | **tetra** |
| OprM Back-bone RMSD (Å) | 2.05±0.08 | 1.84±0.07 | 2.22±0.1 | 1.97±0.07 | 1.75±0.11 | 2.27±0.1 | 2.07±0.08 |
|  | **azithro** | **chloramph** | **levoflo** | **merop** | **NPC100251** | **PRO** | **tetra** |
| OprN Back-bone RMSD (Å) | --- | 1.82±0.08 | 2.94±0.08 | --- | 1.67±0.07 | 2.16±0.1 | 2.25±0.11 |
|  | **azithro** | **chloramph** | **levoflo** | **merop** | **NPC100251** | **PRO** | **tetra** |
| TolC Back-bone RMSD (Å) | 3.09±0.12 | 2.06±0.11 | 1.94±0.1 | 2.14±0.12 | 1.71±0.1 | 2.46±0.12 | 2.17±0.08 |

| 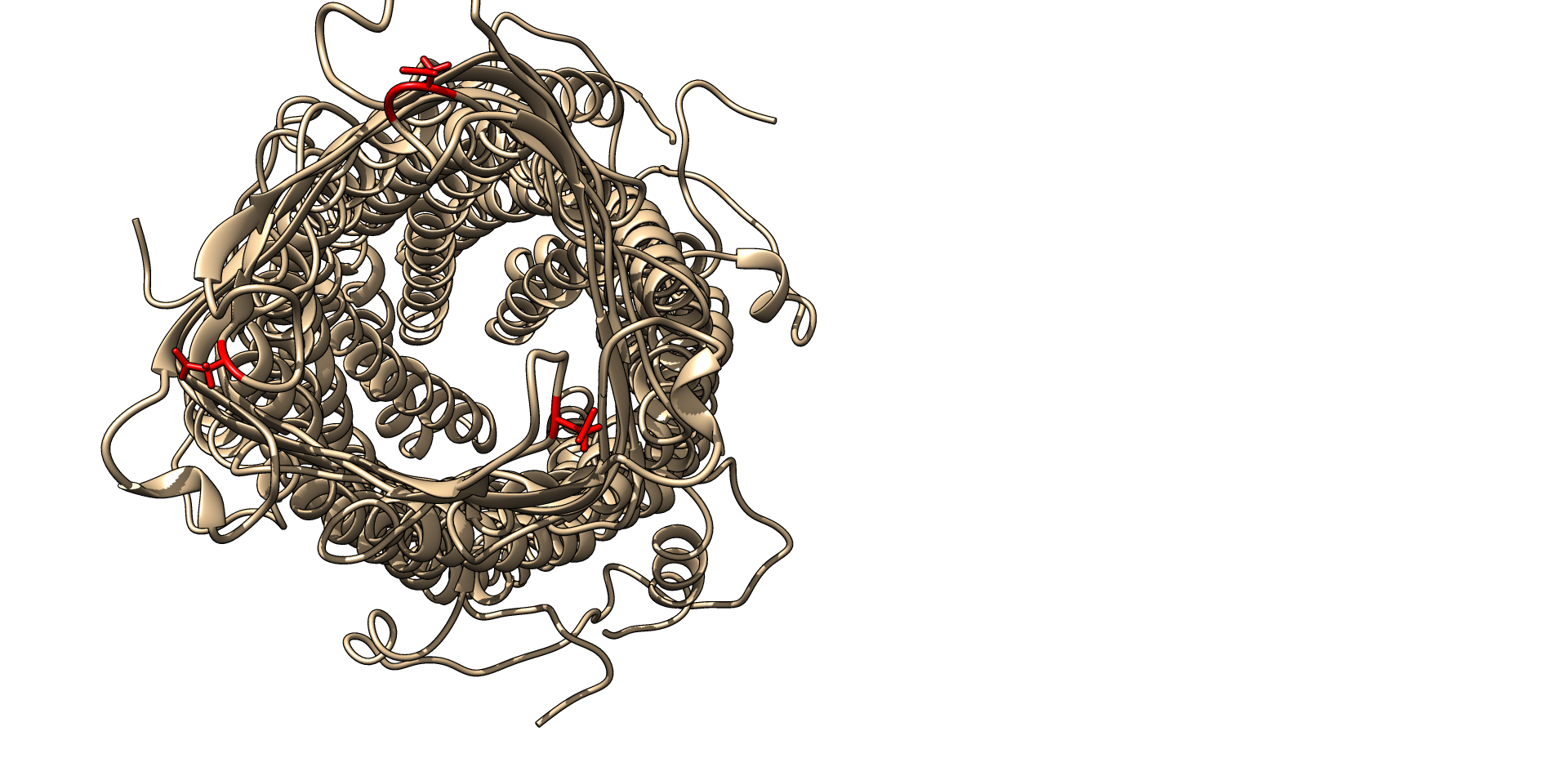 |
| --- |
| 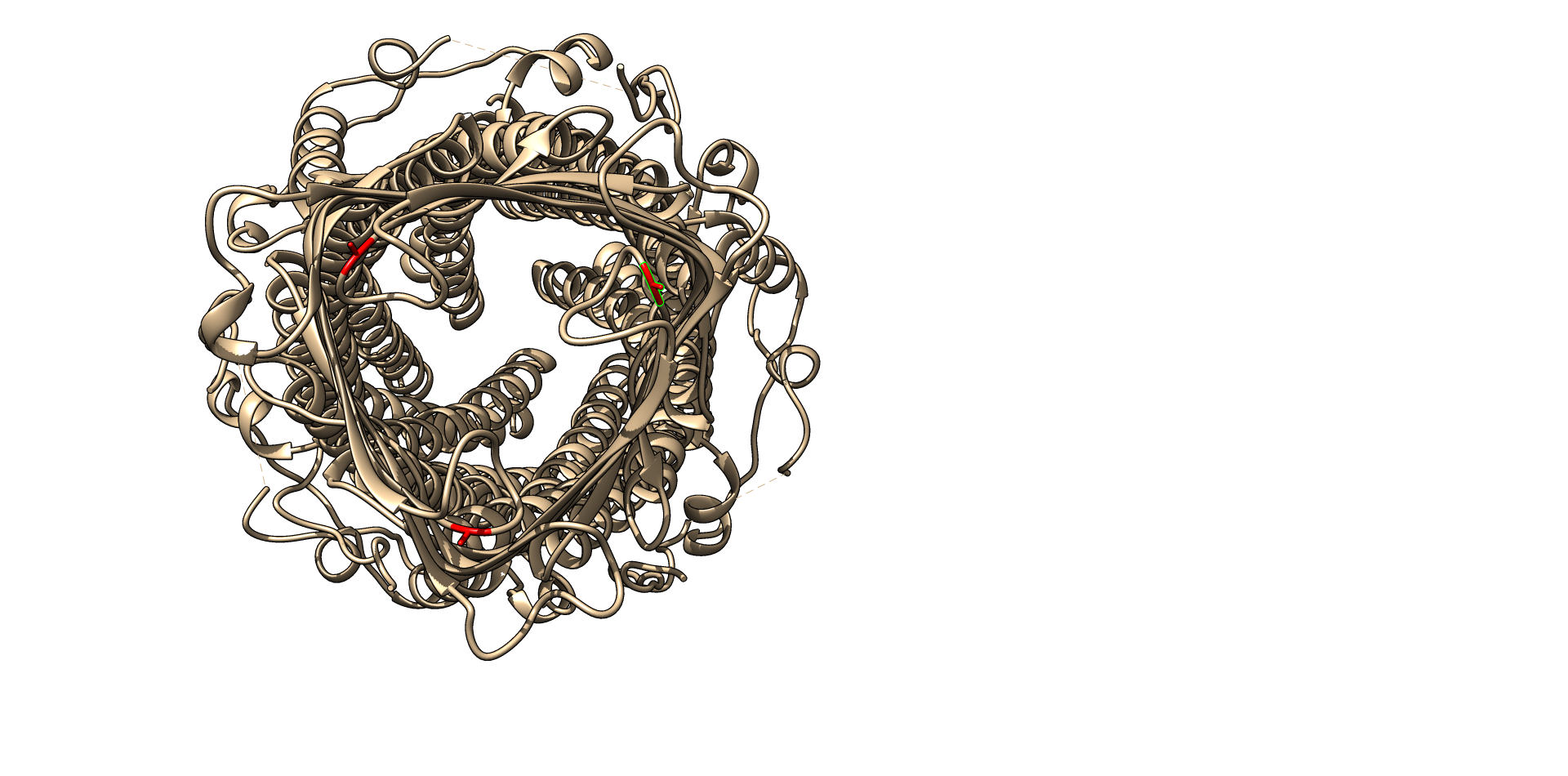 |
| 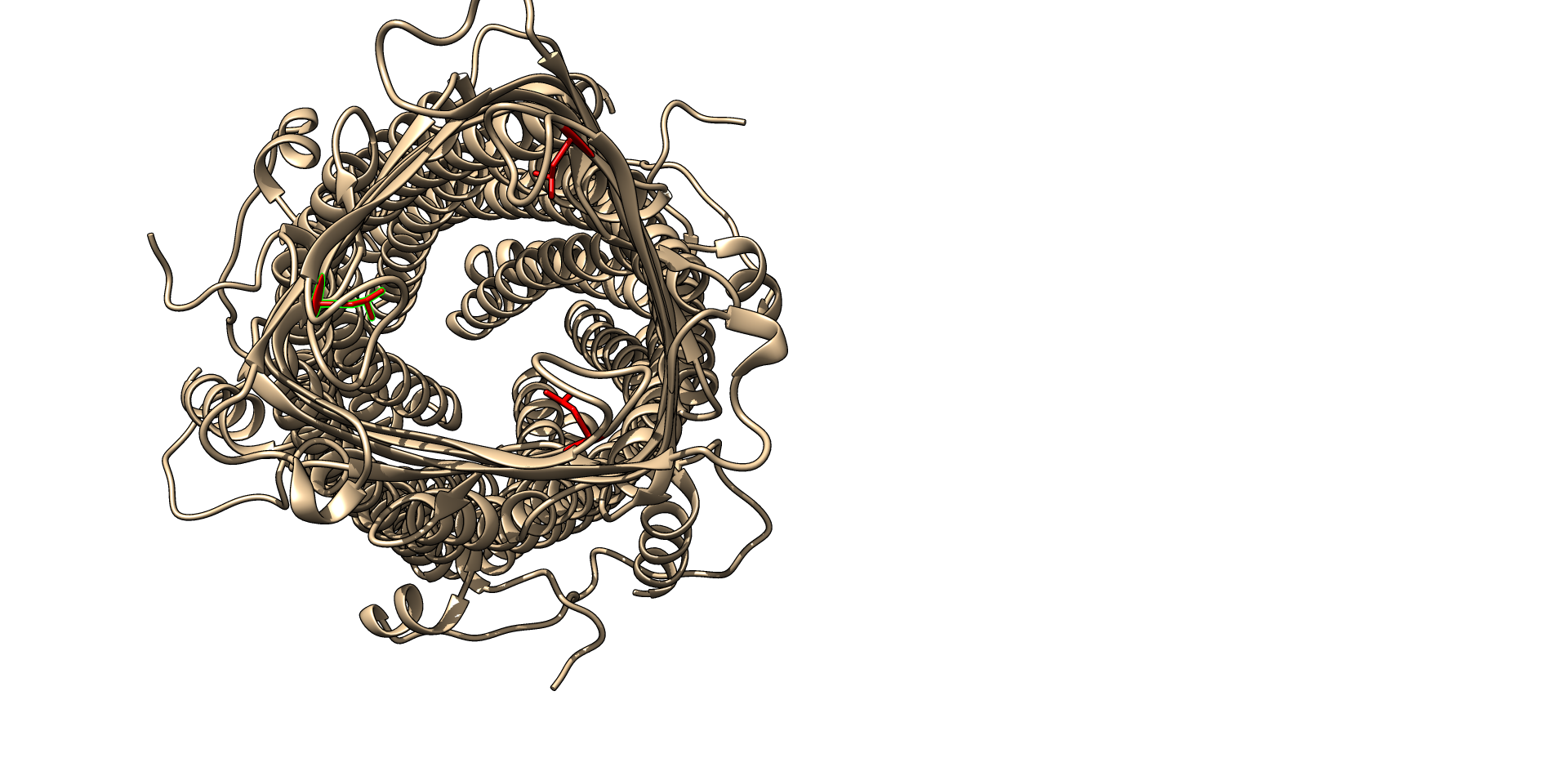 |
| 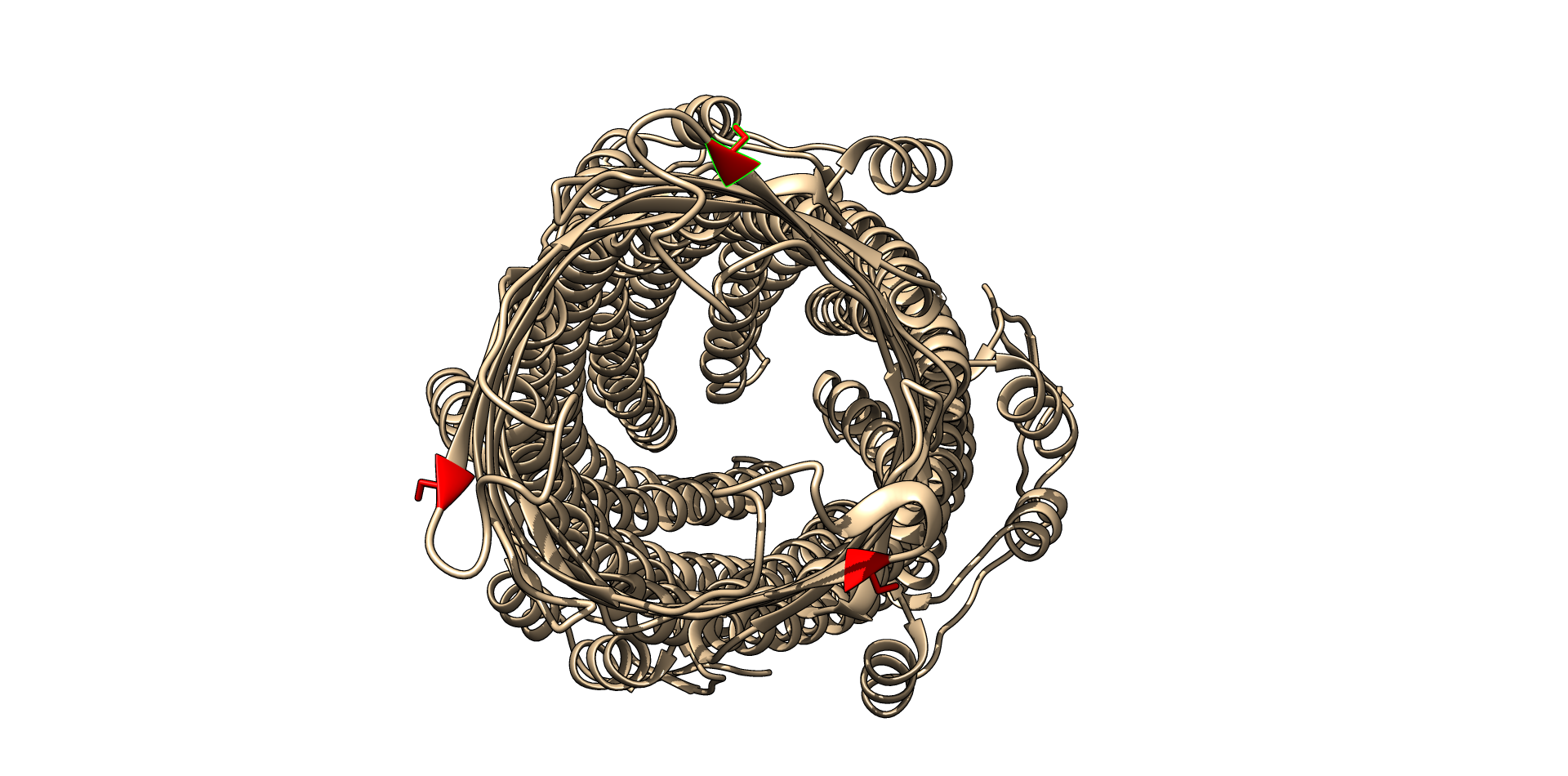 |

Figure S11. A; location Asp158 in OprA, B; position of the Ala96 in OprJ, C; location of the Lue99 in OprM, and D; position of the Ser262 in TolC. Residues were mark in red color.

| **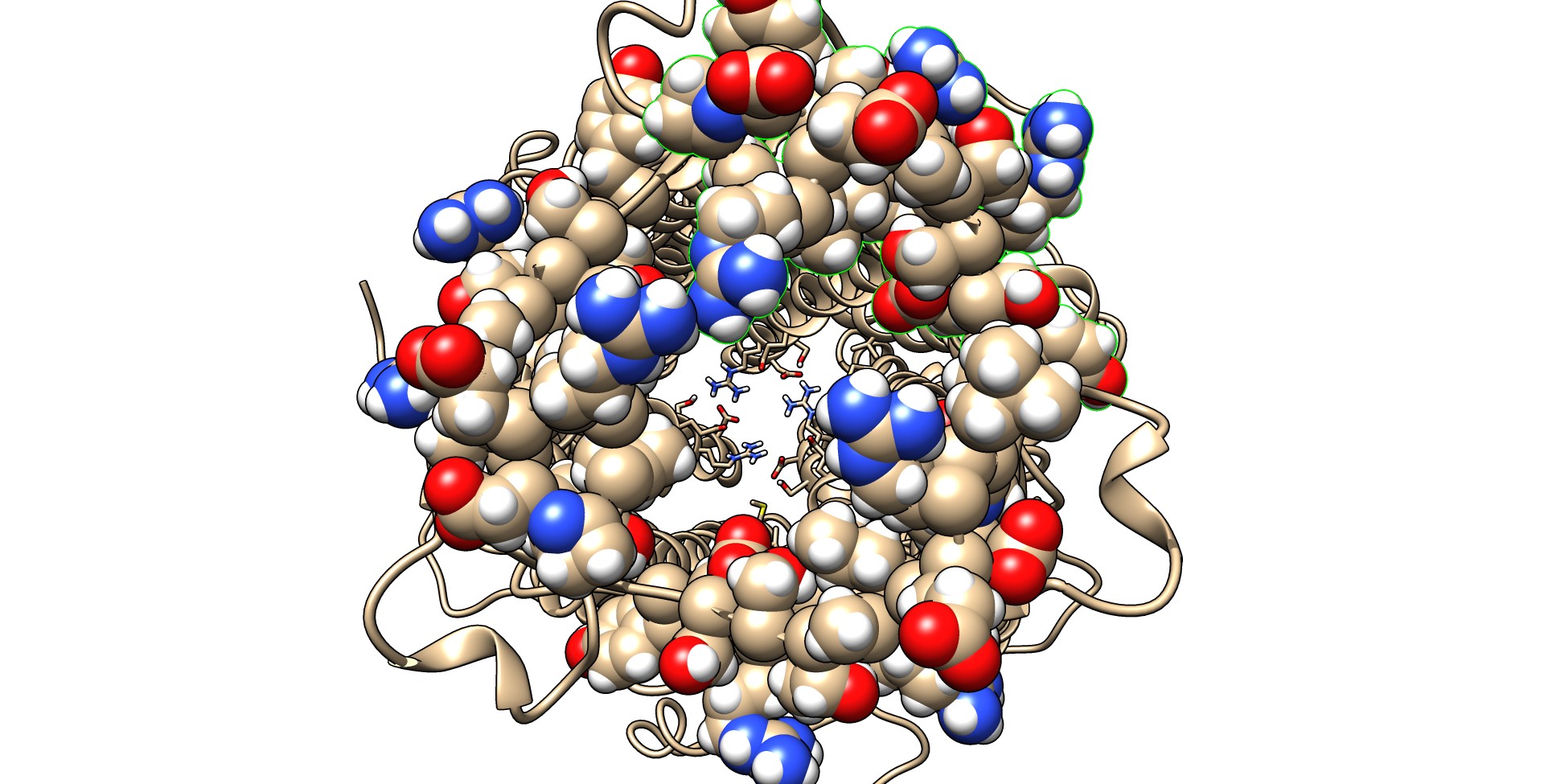** | **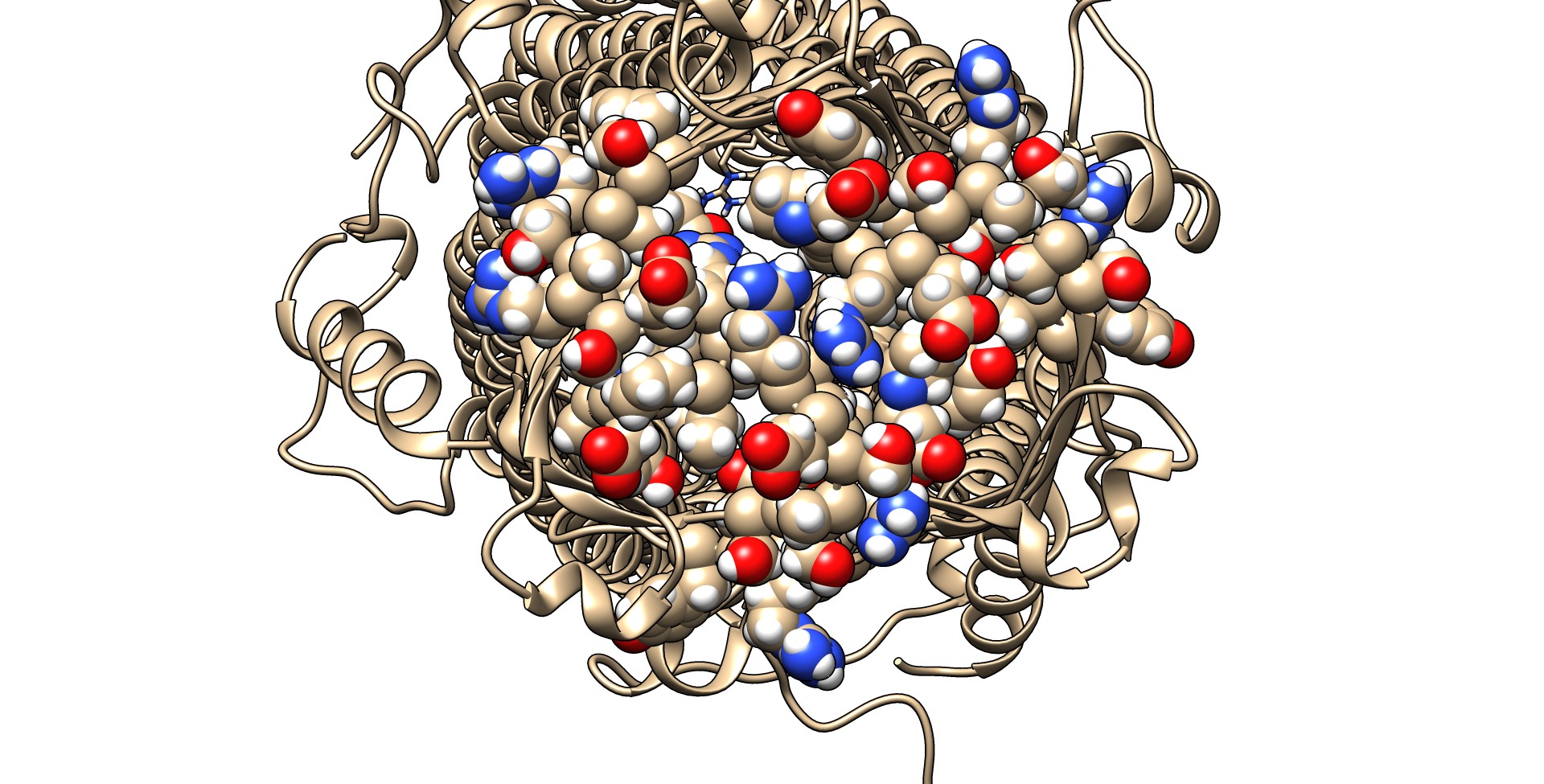** |
| --- | --- |
| **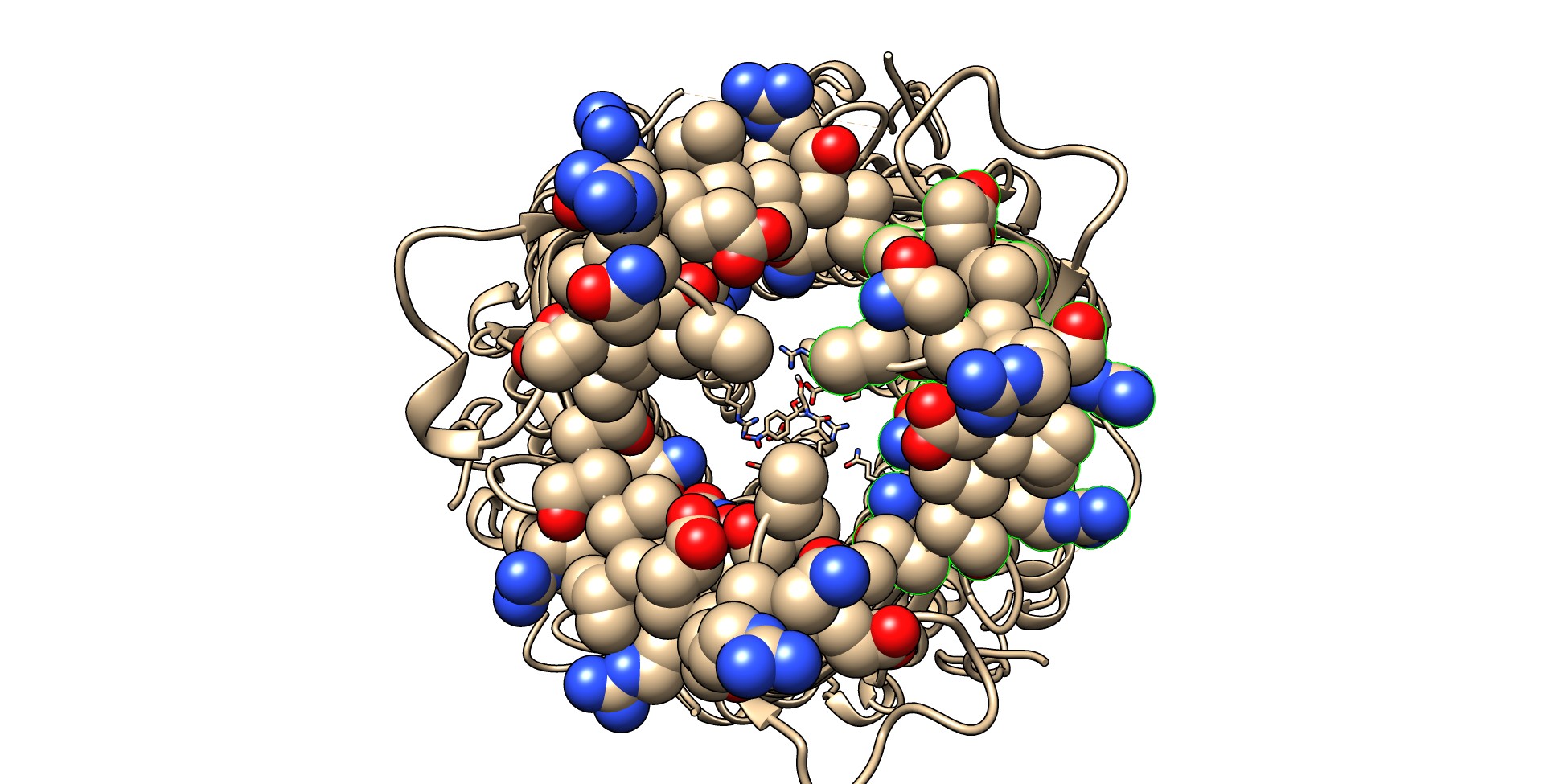** | *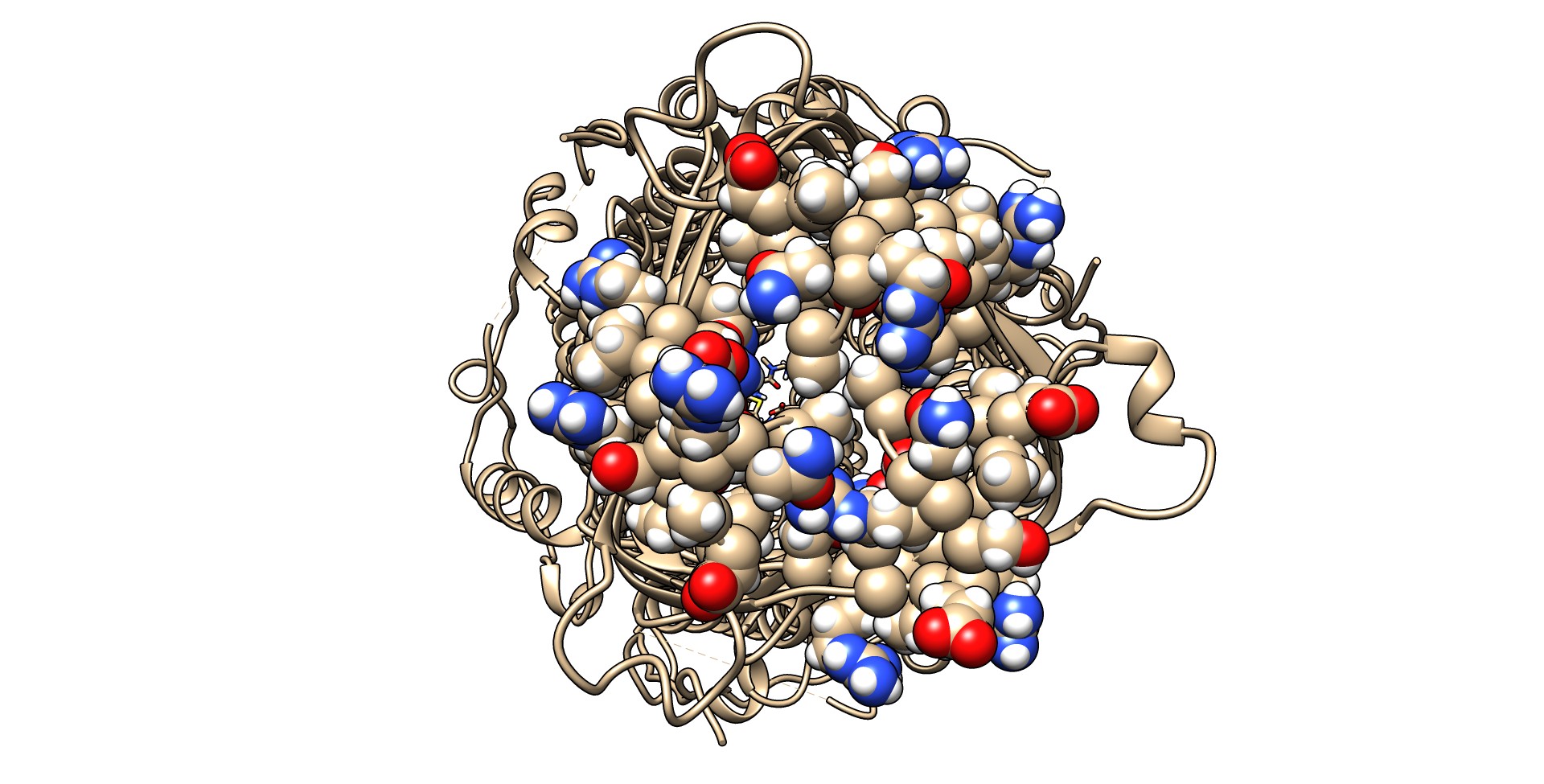* |
| *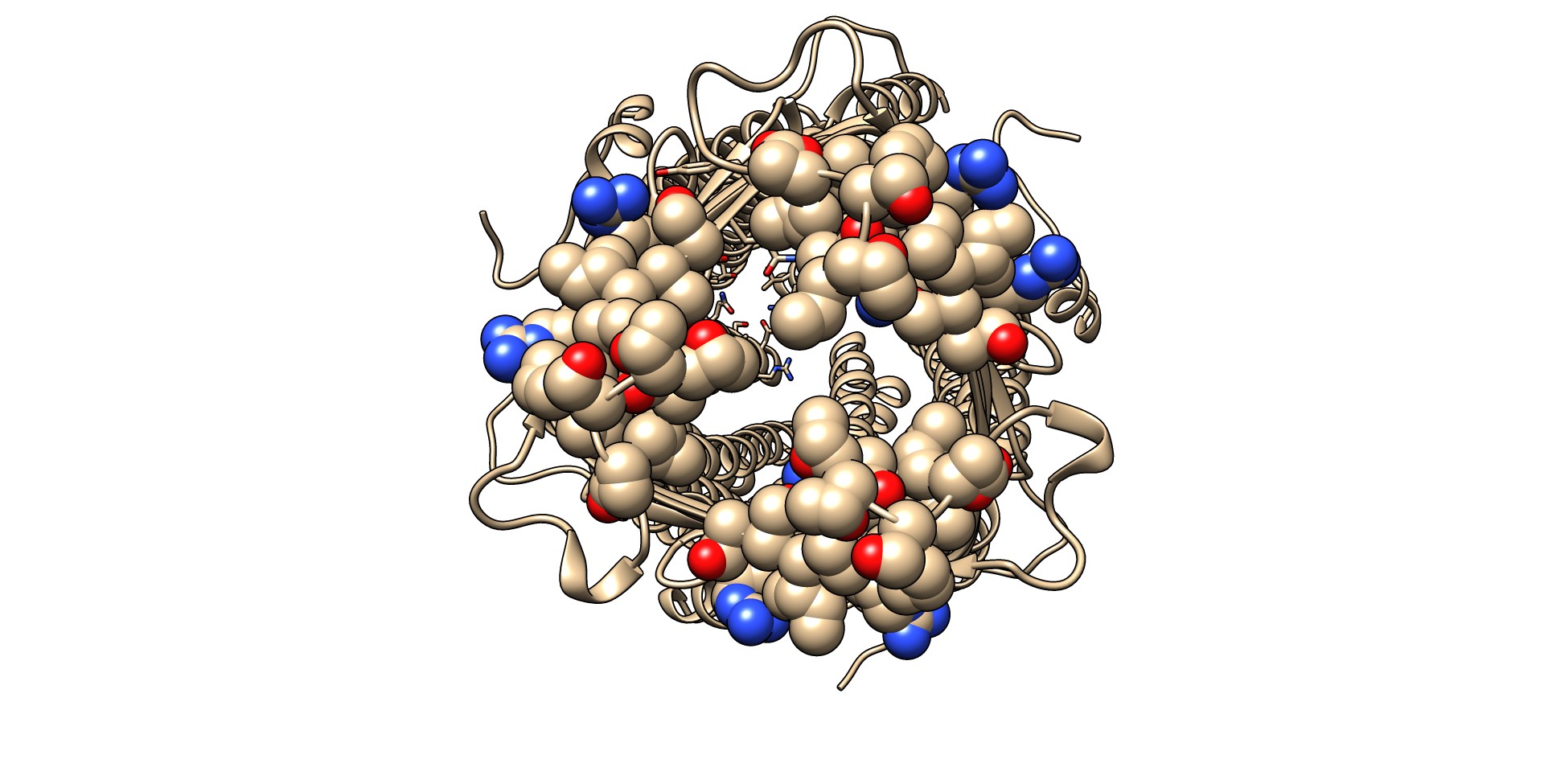* | *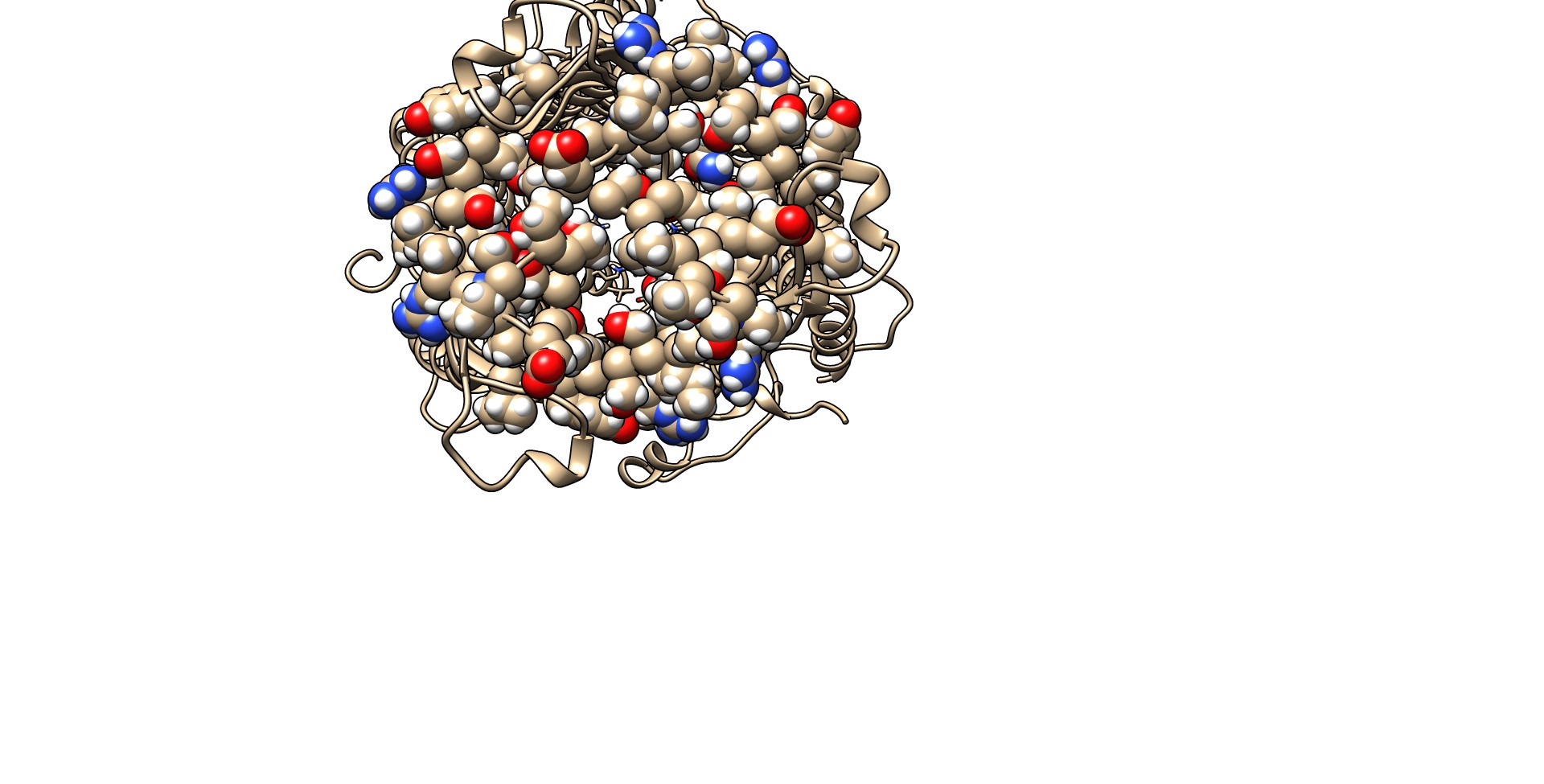* |
| *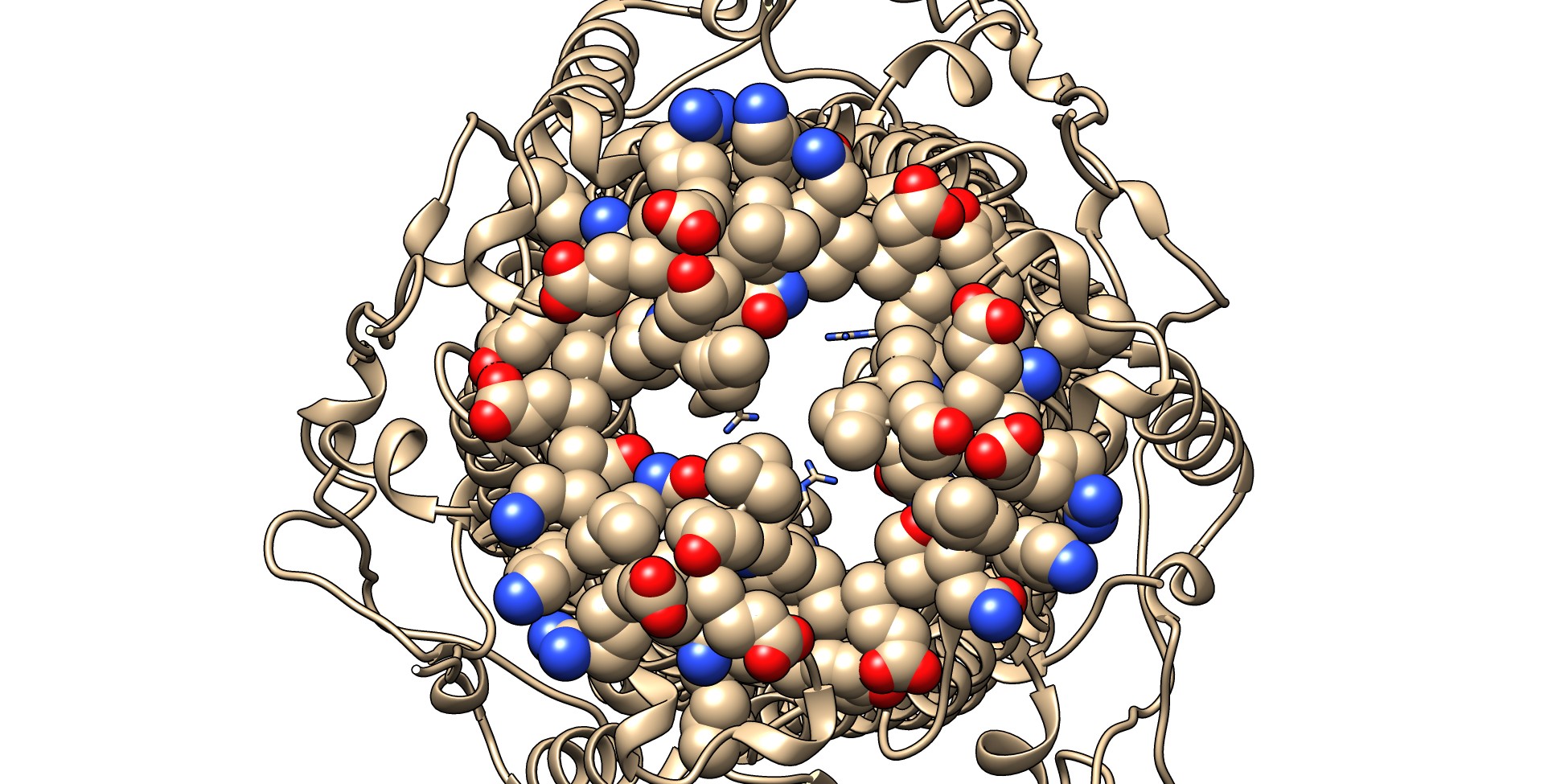* | *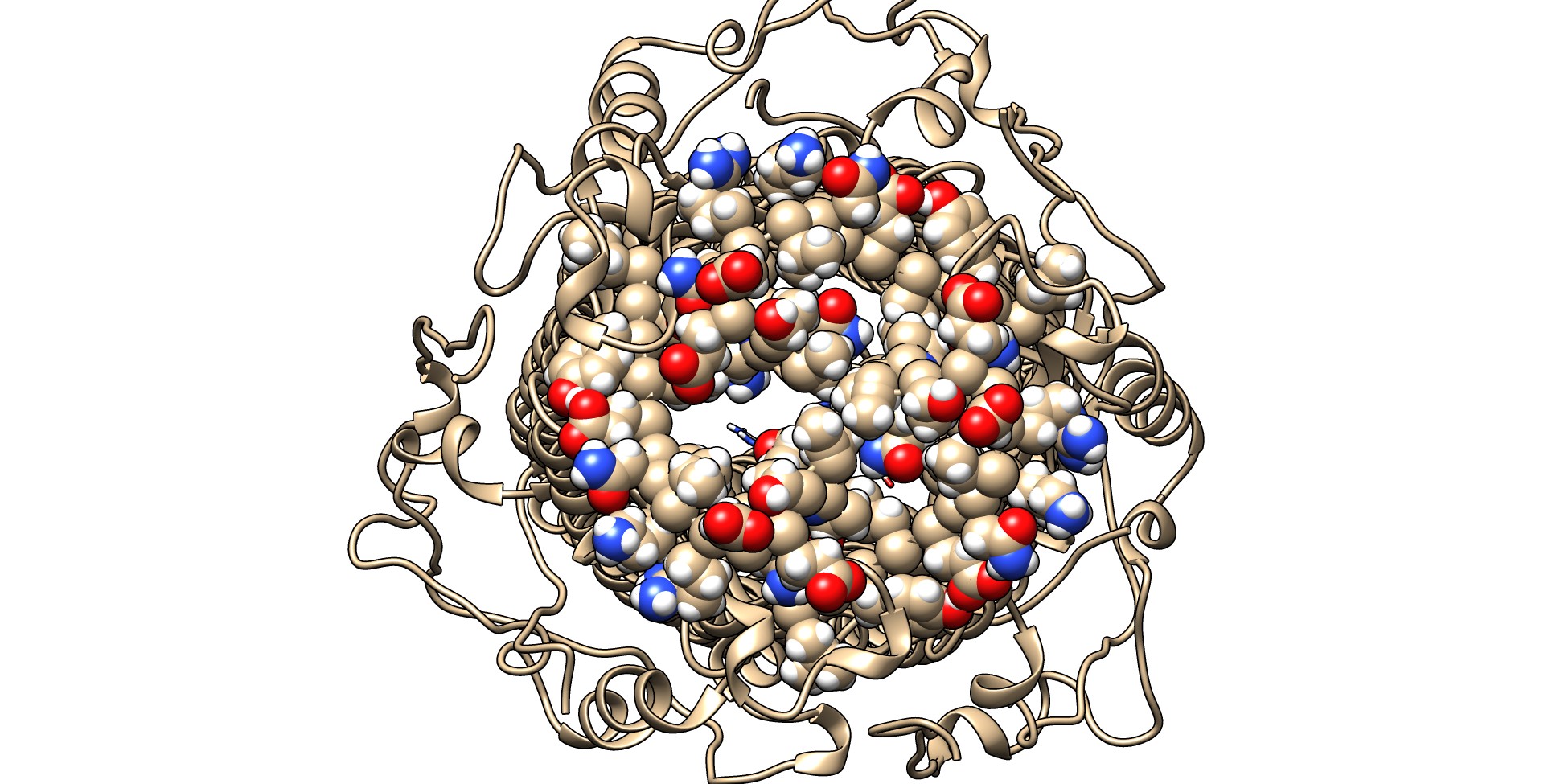* |
| **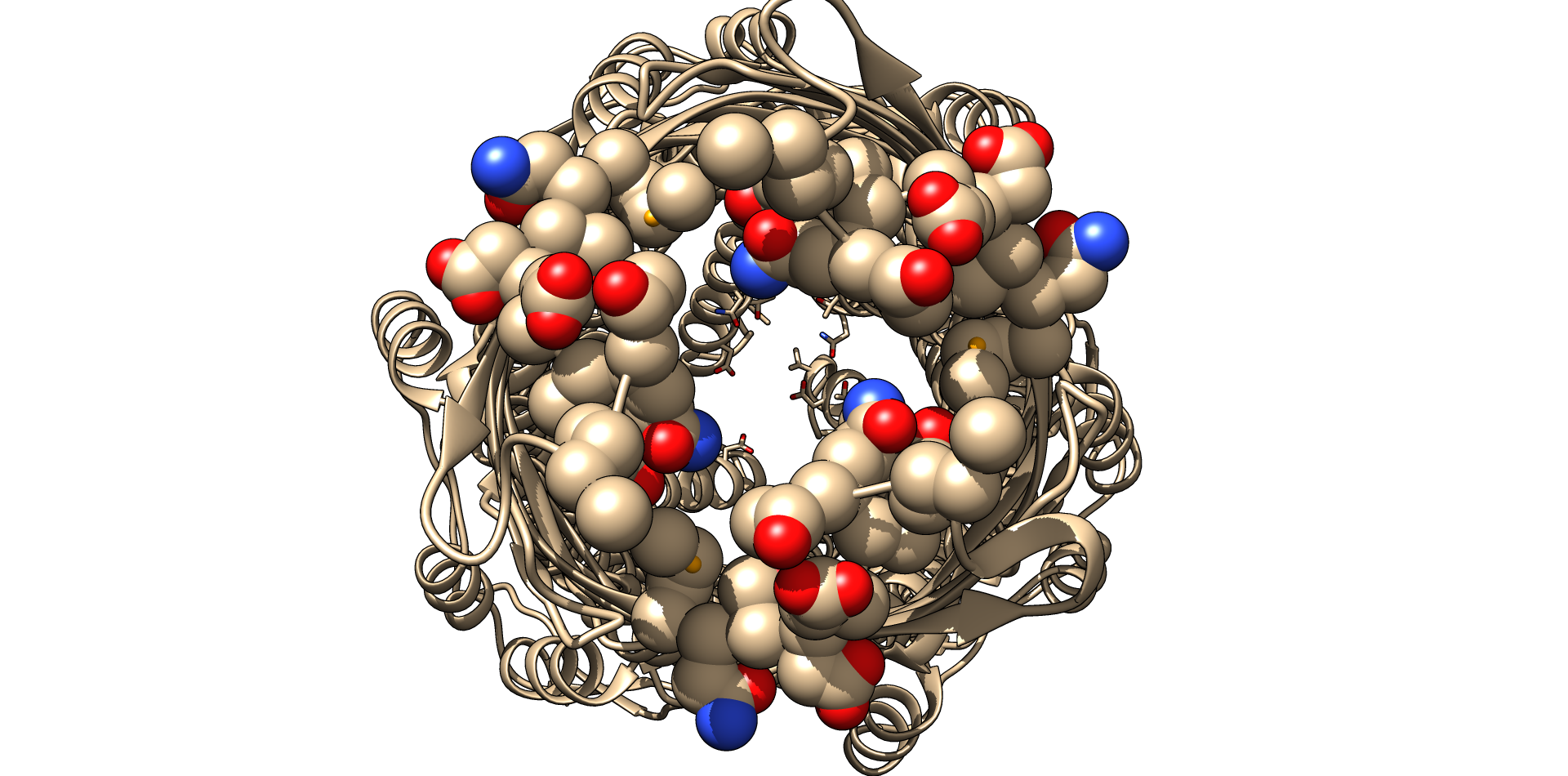** | **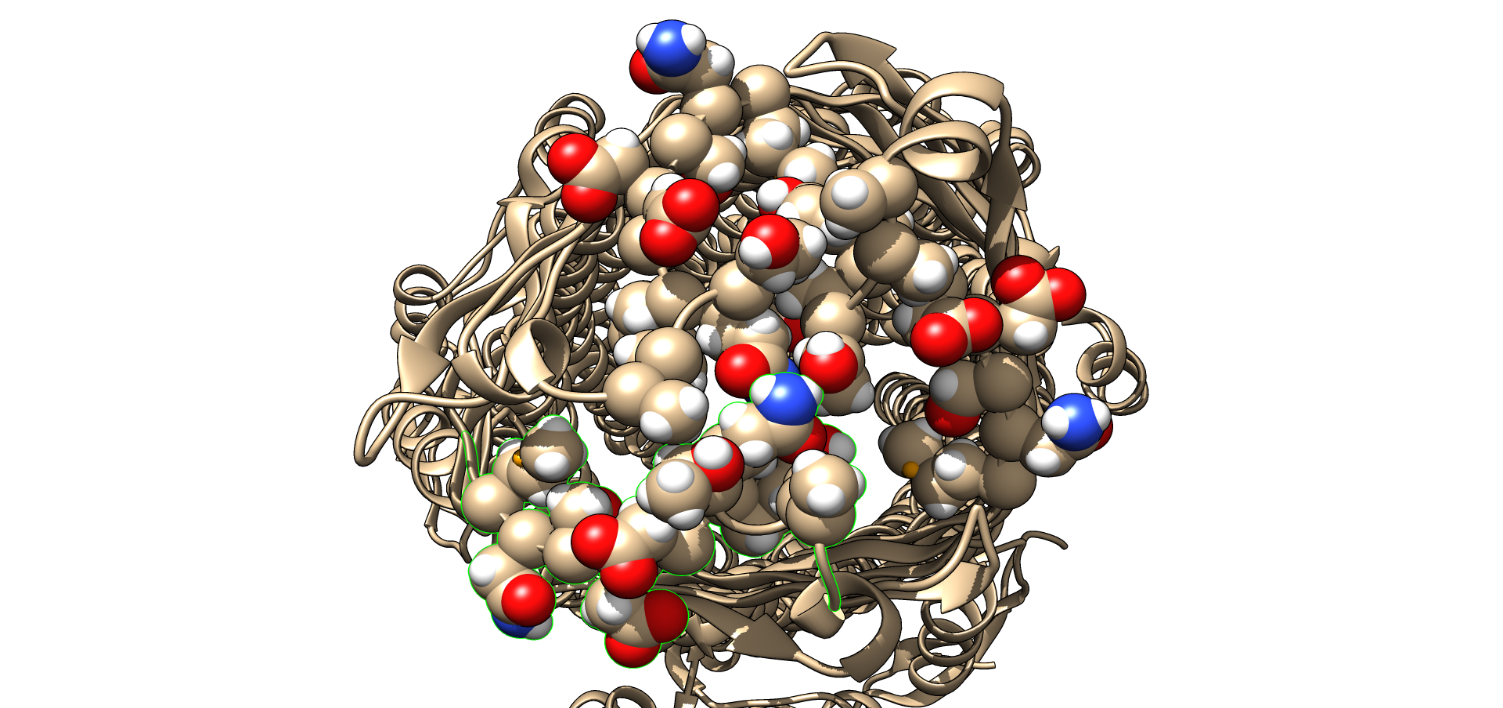** |

Figure *S*12. Swing motion exit gate loop of OMPs. The right-hand pictures the gates are open; the left-hand pictures the gate is closed by swing motion exit gate loop of OMPs.

Table S2. Solubility characteristic.

| Identifier | compound | S+logP | MW (g/mol) | Solubility | Diff. Coeff*(cm^2^/s×10^-5^) | Peff^⸸^  (cm/s×10^-4^) | pKa Microstates |
| --- | --- | --- | --- | --- | --- | --- | --- |
| NPC98583 | NC1 | 4.2 | 882.747 | 0.034 | 0.443 | 0.092 | 12.4 |
| NPC100251 | NC5 | 3.688 | 914.746 | 0.027 | 0.439 | 0.077 | 12.33 |
| NPC112380 | NC7 | 2.9 | 868.72 | 0.068 | 0.448 | 0.129 | 11.89 |
| NPC473010 | NC2 | 6.268 | 668.746 | 0.012 | 0.483 | 0.994 | 11.83 |

* Molecular diffusion coefficient

⸸ human jejunal permeability. Peff < [0.25,0.5] OR MDCK < [20,30] (low permeability)

Table S3. Fast and fed stat solubility.

|  | fasted state intestinal fluid (mg/mL) | fed state intestinal fluid (mg/mL) | fasted state gastric fluid (mg/mL) |
| --- | --- | --- | --- |
| NC1 | 0.004 | 0.064 | 0.00034 |
| NC2 | 0.004 | 0.043 | 0.00038 |
| NC3 | 0.019 | 0.145 | 0.00039 |
| NC4 | 0.002 | 0.145 | 0.00026 |

Table S4. Some other properties which had an impact on the solubility and permeability.

|  | MlogP* | S+logP^⸸^ | S+logD^#^ | logHLC^§^ (atm*m^3^/mol) |
| --- | --- | --- | --- | --- |
| NC1 | -3.658 | 4.2 | 3.553 | -18.786 |
| NC2 | -5.008 | 3.688 | 2.735 | -18.984 |
| NC3 | -3.887 | 2.9 | -1.146 | -18.801 |
| NC4 | 2.149 | 6.268 | 6.107 | -14.771 |

*MlogP Moriguchi model of octanol-water partition coefficient

⸸S+logP: octanol-water partition coefficient (log P)

#S+logD: octanol-water distribution coefficient (log D)

§LogHLC [atm*m3/mol]: logarithm of the air-water partition coefficient, a.k.a. Henry’s Law Constant (Simulations Plus model).

Table S5. Permeability to different membrane and cell.

|  | Perm_Skin* (cm/s×10^7^) | S+MDCK^#^ (cm/s×10^7^) | S+Peff^§^ (cm/s×10^4^) | Perm-Cornea^⸸^ (cm/s×10^7^) | BBB-Filter^¥^ | LogBB^€^ |
| --- | --- | --- | --- | --- | --- | --- |
| NC1 | 104.704 | 0.22 | 0.092 | 22.09 | Low | -0.716 |
| NC2 | 89.65 | 0.129 | 0.077 | 17.76 | Low | -0.688 |
| NC3 | 73.209 | 0.2 | 0.129 | 24.329 | Low | -0.992 |
| NC4 | 43.232 | 19.875 | 0.994 | 100.599 | Low (97%) | -0.183 |

*Perm_Skin [cm/s×107]: permeability through human skin.

#S+MDCK [cm/s×107]: apparent MDCK COS permeability.

§S+Peff [cm/s×104]: human effective jejunal permeability.

⸸Perm_Cornea [cm/s×107]: permeability through rabbit cornea.

¥BBB_Filter: qualitative likelihood (High/Low) of crossing the blood-brain barrier.

€LogBB: logarithm of the brain/blood partition coefficient.

Table S6. Possible metabolism of natural compounds by CYP.

| CYP types | Effects | NPC types |
| --- | --- | --- |
| CYP1A2 | substrate | NPC112380 |
|  | inhibitor | - |
| CYP2C8 | substrate | NPC473010 |
|  | inhibitor | - |
| CYP2C9 | substrate | - |
|  | inhibitor | NPC473010 |
| CYP2E1 | substrate | NPC98583, NPC100251 and NPC112380 |
|  | inhibitor | - |
| CYP2D6 | substrate | - |
|  | inhibitor | - |
| CYP2E1 | substrate | - |
|  | inhibitor | - |
| CYP3A4 | substrate | All compounds |
|  | inhibitor | NPC473010 |

Table S7. Mechanism of xenobiotic elimination by UGT (Uridine5′-diphosphate-glucuronosyltransferases family) enzymes

|  | UGT1A1 | UGT1A3 | UGT1A4 | UGT1A6 | UGT1A8 | UGT1A9 | UGT1A10 | UGT2B7 | UGT2B15 |
| --- | --- | --- | --- | --- | --- | --- | --- | --- | --- |
| NC1 | No | Yes | No | No | Yes | No | Yes | No | No |
| NC2 | No | Yes | No | No | Yes | Yes | Yes | No | No |
| NC3 | Yes | Yes | No | No | Yes | No | Yes | No | No |
| NC4 | Yes | Yes | No | No | Yes | No (61%) | (67%) | No | NO |

Table S8. Inhibitor and substrate for P-glycoprotein efflux.

|  | Pgp_Substr^1^ | Pgp_Inh^2^ | OATP1B1_Inh^3^ |
| --- | --- | --- | --- |
| NC1 | Yes (83%) | Yes | Yes (91%) |
| NC2 | Yes (83%) | Yes | Yes (91%) |
| NC3 | Yes (83%) | Yes | Yes (91%) |
| NC4 | Yes (83%) | Yes | Yes (91%) |

1. Pgp_Substr: likelihood of P-glycoprotein efflux substrate.

2. Pgp_Inh: likelihood of P-glycoprotein inhibition.

3. OATP1B1_Inh: inhibition of the hepatic OATP-1B1 transporter.

Table S9. Possible mechanism of clearance and clearance classification.

|  | S+CL_Metab^1^ | S+CL_Renal^2^ | S+CL_Uptake^3^ | S+CL_Mech^4^ | ECCS_Class^5^ |
| --- | --- | --- | --- | --- | --- |
| NC1 | Yes | No | Yes | HepUptake | Class_3B; High_MWt |
| NC2 | Yes | No | Yes | HepUptake | Class_3B; High_MWt |
| NC3 | Yes | No (92%) | Yes | HepUptake | Class_3B; High_MWt |
| NC4 | Yes | No (75%) | Yes | Metabolism | Class_4 |

1. S+CL_Metab: predicts whether or not metabolism will be critical to clearance.

2. S+CL_Renal: predicts whether or not renal elimination will be critical to

clearance.

3. S+CL_Uptake: predicts whether or not hepatic uptake will be critical to

clearance.

4. S+CL_Mech: predicts whether metabolism, renal elimination or hepatic

uptake will limit clearance.

5. ECCS_Class: Extended Clearance Classification System (ECCS) assignment.
